# Frameworks and methods for estimating the causal effect of clinical treatment in orthopaedics: a scoping review

**DOI:** 10.1530/EOR-2025-0266

**Published:** 2026-07-01

**Authors:** Bastian Widmer, Michael Ketzer, Michael Simon, Giusi Moffa, Elke Viehweger, Morgan Sangeux

**Affiliations:** ^1^ Department Mathematics and Computer Science, University of Basel, Basel, Switzerland; ^2^ Institute of Nursing Science, Department Public Health, University of Basel, Basel, Switzerland; ^3^ Directorate of Nursing, Therapeutic Services, and Social Work, University Psychiatric Clinics Basel, Basel, Switzerland; ^4^ Centre for Motion Analysis, University Children’s Hospital Basel (UKBB), Basel, Switzerland; ^5^ Department of Orthopaedics, University Children’s Hospital Basel (UKBB), Basel, Switzerland; ^6^ Department of Biomedical Engineering, University of Basel, Basel, Switzerland

**Keywords:** causal inference, propensity score, target trial emulation, directed acyclic graph, orthopaedic procedures, scoping review

## Abstract

- The gold standard for establishing the causal effect of a clinical treatment is a randomized controlled trial, which is challenging to apply in orthopaedics, mainly due to ethical reasons. Therefore, retrospective observational studies were conducted to elucidate potential treatment effects. However, clinicians rarely receive guidance on how to apply modern causal inference methods to strengthen conclusions drawn from such data and support evidence-based decision-making. The objective of this scoping review was to facilitate a better understanding of the use of causal inference methods in orthopaedics.
- The scoping review adheres to the Joanna Briggs Institute methodology and the five-stage framework of Arksey and O’Malley. Two themes, ‘orthopaedic interventions’ and ‘causal inference’, guided the search strategy development for this scoping review. A literature search of observational studies was conducted using the PubMed, Cochrane Central Register of Controlled Trials, EMBASE, and SCOPUS databases. Studies describing the use of causal inference frameworks in clinical treatment in orthopaedics were included.
- Of 6,244 records, 85 studies were included in the study. Propensity score matching and inverse probability of treatment weighting were the most common methods. Multicentric studies predominated, while the use of formal causal inference frameworks, such as target trial emulation and directed acyclic graphs, was rare.
- Causal inference is increasingly used in orthopaedics, although formal frameworks, such as target trial emulation and causal diagrams, remain underutilized. We recommend using prospective, publicly available protocols that incorporate these tools to enhance transparency and validity.

## Introduction

The gold standard for establishing the causal effect of a clinical treatment is a randomized controlled trial (RCT), which can be challenging to implement in orthopaedics due to practical and ethical considerations. Malavolta *et al.* described several difficulties in conducting RCTs in the field of orthopaedics (1); fundamental steps, such as patient selection, randomization, and blinding, raise challenges when surgical procedures are involved. First, recruitment can be challenging because patients often have a strong preference for surgical treatment. New and experimental surgical procedures can cause patients to be anxious and apprehensive to a greater extent compared to new medications, knowing that surgery is often permanent and there is no washout period, which allows the treatment to be eliminated from the patient’s system, as can be found with the application of medications. Second, randomization is particularly difficult because the surgical techniques must be randomized. Any limitation to carrying out one of the surgeries, such as only on certain days or with a specific surgeon, can compromise this principle. For example, complex emergency or trauma surgery may be subject to the availability of surgeons with a particular skill set, which would impair randomization. Other difficulties may arise in studies comparing different implants. In an ideal situation, assignment to treatment groups should be made at the time of surgery; however, preparation for the procedure often requires planning, and the team needs to be prepared, which can lead to blinding loss. Another problem is that inclusion for surgery can only be defined after intraoperative evaluation, for example, suturing a meniscal tear. In such cases, intraoperative randomization is necessary. In studies involving nonsurgical groups, blinding of the surgeon is not possible. Furthermore, when required by the RCT, the use of placebo surgery raises serious ethical concerns. In studies comparing two surgical interventions, if the operative techniques differ, blinding of the surgeon is impossible, and bias could be introduced if the surgeon favours one technique and makes greater efforts towards it. Blinding of patients is compromised when different access routes are used. If different treatments used different implants, the patient could also notice differences between implants if they had access to radiographic examinations. Blinding of independent assessors, typically physicians or physiotherapists, may be compromised when different techniques employ different access routes, resulting in distinct scars. Blinding may also be lost when different surgical interventions require different types of rehabilitation. In summary, many factors make conducting RCTs in orthopaedics impractical. In the absence of RCTs, observational studies can provide valuable evidence for comparing treatments. When using observational data to estimate the effects of individual interventions, it is beneficial to frame studies in a formal causal inference framework to enhance transparency and quality (2).

Causal inference is the process of determining cause-and-effect relationships between variables based on evidence, observations, and reasoning (3). Target trial emulation (TTE) and causal diagrams in the form of directed acyclic graphs (DAGs) have gained momentum as tools for implementing a formal causal inference framework (4), while enhancing communication clarity in terms of estimands and assumptions. Common statistical methods to adjust for confounding include inverse probability of treatment weighting (IPTW), propensity score matching (PSM), and instrumental variable (IV) analysis (5, 6).

In this context, high-quality observational data sources become essential for estimating treatment effects. While some orthopaedic subfields rely on large registries or administrative data sets, others, such as neuro-orthopaedics, benefit from highly structured, detailed functional assessments. Clinical gait analysis (CGA) is one such example, providing a very rich data set, including kinematics and kinetics of the lower limb joints during walking, to document the patient’s motor function pre- and post-treatment (7). There are well-established outcome measures based on CGA to evaluate the effectiveness of interventions aimed at improving walking function. For example, the gait profile score provides an accurate assessment of deviations from normal gait, including their extent and location (8, 9), while the gait standard deviation (10) measures the variability in the gait pattern, which varies based on primary motor disorders. Therefore, many major neuro-orthopaedic centres routinely collect gait analysis data to guide surgical decisions and evaluate treatment outcomes.

While causal inference has demonstrated promise in evaluating the impact of treatment by showing consistency with results from RCTs targeting the same estimand (11, 12), its use, methodological quality, and integration into a formal framework remain unclear. This scoping review maps the landscape of causal inference in orthopaedics, identifying current practices, gaps, and opportunities for methodological advancement. Furthermore, we will review if and how rich data sets provided by CGA may facilitate causal inference methods in orthopaedics.

## Methods

The review process was guided by the Joanna Briggs Institute (JBI) methodology for scoping reviews (13). This scoping review drew inspiration from Arksey and O’Malley’s five-stage approach (14). We discuss each stage briefly below. For more details, a registered protocol is available (15).

### Stage 1: identifying the research question

This study assessed the current state of the art for estimating the causal effects of clinical treatments in orthopaedics from observational studies. The primary objective focuses on mapping practices in terms of frameworks and methods informing the causal inference of clinical treatments in orthopaedics. The following key questions guided the scoping review:

- What causal inference frameworks have been adopted to assess the effect of clinical treatment in orthopaedics?
- What was the origin and volume of the data used for the assessment?
  - Research or clinical data.
  - Monocentric or multicentric settings.
  - Number of observations.
- Which interventions and outcomes were investigated?

### Stage 2: identifying relevant studies

We used the population, concept, and context (PCC) framework to determine eligibility for the scoping review. A literature search was conducted across the PubMed, Cochrane Central Register of Controlled Trials, EMBASE, and SCOPUS databases. The initial database used for the search was PubMed, and the search string was subsequently adapted for other databases using the Polyglot Search Translator (16). Finally, references were added through personal libraries.

The search strings were run concurrently on 21 May 2025. The results were imported into Rayyan (Rayyan Systems Inc., USA), a web application for systematic reviews (17). Duplicates were automatically identified and manually removed.

### Stage 3: study selection

The study selection process consisted of two steps: first, the titles and abstracts were screened, followed by a full-text review. Each step was conducted independently by two separate reviewers. It was determined whether studies assessed the effect or association of orthopaedic clinical treatments using causal inference by evaluating whether their stated objective and methodology explicitly aimed to estimate causal treatment effects. Eligible studies employed approaches such as PSM, IPTW, IV, or TTE with the aim of reducing bias. Importantly, studies that separated populations only by medical condition, rather than by receipt of orthopaedic treatment, were excluded because they did not estimate treatment effects. Additional inclusion criteria required that studies be full-text and observational in nature. Disagreements emerging among the reviewers were resolved by consensus in regular sessions between the two reviewers.

Following title and abstract screening, a large number of eligible studies were identified. In particular, 804 studies reported the use of PSM. Given the large number of candidates for full-text screening, it was not feasible to perform detailed full-text extraction for all records within the scope of this scoping review. Therefore, we purposively selected a sample that maximized diversity of the causal inference methods used. Selection focused on studies providing indications of an explicit causal approach. A causal approach was defined as the explicit conceptualization of the relationship between exposure and outcome in terms of causation, including the identification of confounders and consideration of the underlying data-generating process. Indications of such reasoning were identified using observable markers of causal inference practice, including the use of DAGs, publicly available protocols, TTE, rationale for variable selection, and statements of causal intention (see Supplementary material Table E (see section on Supplementary materials given at the end of the article)). This approach is consistent with qualitative and methodological evidence syntheses, in which analysis may focus on a subset of studies to provide a broad overview of a field rather than statistical representativeness (18). Consequently, the subset of 85 studies should not be interpreted as representative of all eligible studies.

### Stage 4: charting the data

Two reviewers independently extracted the data, and disagreements were resolved by consensus. Key information from the source was collected using a charting table adapted from the Joanna Briggs Institute’s data extraction template (13). Extracted study characteristics included citation details (e.g. author/s, year of publication, and title), inclusion or exclusion criteria (e.g. population, concept, and context), evidence source details (e.g. aims, participants, and intervention type), and details extracted from source evidence (e.g. causal inference methods used, use of target trial, and use of causal graphs).

Two reviewers independently assessed the risk of bias of the study using the Cochrane’s Risk of Bias in Non-randomized Studies – of Interventions (ROBINS-I) tool (19). Critical appraisal was conducted to provide a transparent evaluation of the methodological robustness of the included evidence. Given that the application of causal inference methods in orthopaedics is relatively new, it was essential to determine how rigorously these methods were implemented and reported. For visualizing risk-of-bias assessments, we used the RobVis web application (20).

### Stage 5: collating, summarizing, and reporting the results

A descriptive analysis was performed using the included literature. An overview of the causal inference methods used to evaluate the effectiveness of clinical treatment in orthopaedics is provided. The methods are put in relation to the use of target trials, centre type, the use of causal graphs, and the year of publication. Finally, a summary of the findings is described through narrative synthesis.

## Results

**Figure 1:**
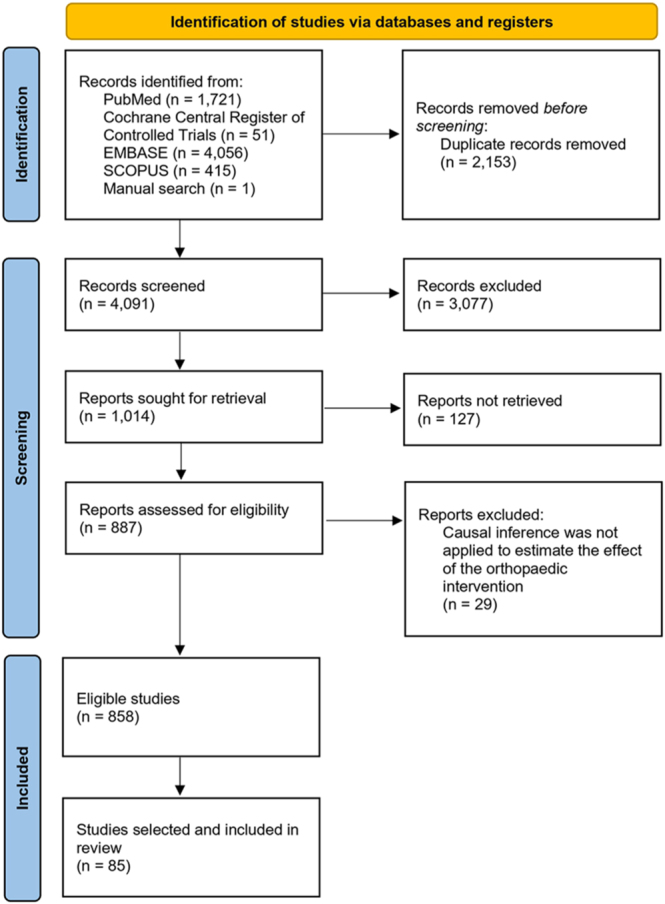
PRISMA flow diagram.

A total of 6,244 records were retrieved from the databases: PubMed (*n* = 1,721), Cochrane Central Register of Controlled Trials (*n* = 51), EMBASE (*n* = 4,056), SCOPUS (*n* = 415), and one manually identified record (a relevant PubMed-indexed publication not captured by the search string). Removing duplicates resulted in 4,091 records. After removing titles and abstracts that did not meet the inclusion criteria, 1,014 records remained, from which we retrieved 887 full-text articles. While screening the abstracts on Rayyan, we labelled the studies according to the causal inference method used. Of the resulting 858 eligible studies for full-text screening, 804 reported the use of PSM. Given the large number of candidates for full-text screening, we selected a subset of studies that used PSM. To be prioritized for full-text screening, these studies also had to show indications of causal intention, identified through specific keywords. Ultimately, 31 PSM studies and 54 studies employing other causal inference approaches met all criteria, resulting in a total of 85 studies included in the final review. Figure 1 illustrates the selection process. Refer to the Supplementary material for a reference list of all included studies.

### Study characteristics

The main characteristics of the included studies are summarized in Table 1, and all study-level details are provided in Supplementary Tables G, H, I, J, and K. The selected articles included original observational studies published between 2013 and 2025. Most of the selected studies were conducted in the USA (*n* = 34), Japan (*n* = 12), or Australia (*n* = 6). Overall, studies were conducted in 19 different countries. The data sources included hospital or institutional clinical data (*n* = 37), registries (*n* = 30), claims databases (*n* = 17), and previous studies (*n* = 3).

**Table 1:** Characteristics of included studies (*n* = 85). Data are presented as *n* (%). Note that studies may have more than one data source type or primary outcome.

Characteristics	Values
Country	
USA	34 (40%)
Japan	12 (14.1%)
Australia	6 (7.1%)
Others*	33 (38.8%)
Source of data	
Unicentric clinical data	24 (28.2%)
Multicentric clinical data	13 (15.3%)
Claims databases	17 (20%)
Registries	30 (35.3%)
*Post hoc* data from RCTs or previous studies	3 (3.5%)
Number of observations	
0–100	10 (11.8%)
101–500	25 (29.4%)
501–1,000	8 (9.4%)
1,001–5,000	14 (16.5%)
>5,000	28 (32.9%)
Treatments	
Joint replacement	30 (35.3%)
Spinal surgery	24 (28.2%)
Soft tissue/tendon/ligament	18 (21.2%)
Bony deformity correction	11 (12.9%)
Physical therapy	2 (2.4%)
Primary outcomes	
Complications	22 (25.9%)
Functional outcomes	8 (9.4%)
Hospitalization outcomes	4 (4.7%)
Mortality	8 (9.4%)
PROMs	29 (34.1%)
Radiographic outcomes	7 (8.2%)
Revision/reoperation	30 (35.3%)
Others^†^	9 (10.6%)
Protocol	
Not publicly available	80 (94.1%)
Publicly available	5 (5.9%)

* ‘Others’ include Netherlands (*n* = 5), South Korea (*n* = 4), Sweden (*n* = 3), UK (*n* = 3), Denmark (*n* = 2), France (*n* = 2), Germany (*n* = 2), Italy (*n* = 2), Switzerland (*n* = 2), Taiwan (*n* = 2), Austria (*n* = 1), Belgium (*n* = 1), China (*n* = 1), Israel (*n* = 1), Portugal (*n* = 1), and Thailand (*n* = 1).

^†^ ‘Others’ include operative time (*n* = 4), blood loss (*n* = 4), tourniquet time (*n* = 1), drainage (*n* = 1), haemoglobin level (*n* = 1), analgesic use (*n* = 3), posttraumatic osteoarthritis (*n* = 1), and range of motion deficit (*n* = 2).

**Figure 2:**
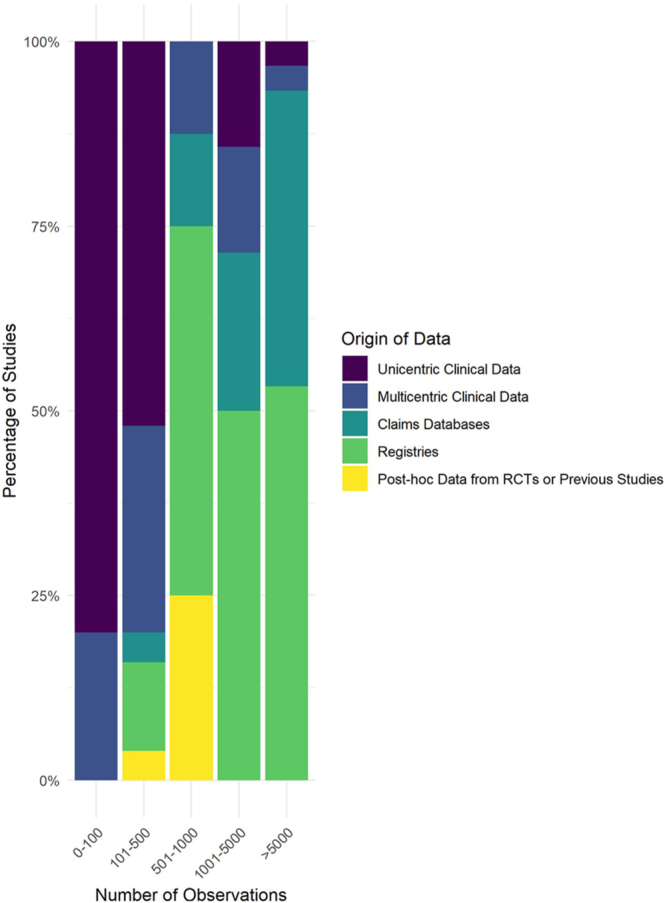
Relative use of different data sources across study sample sizes.

Small data sets with fewer than 100 observations have been reported only in studies using clinical data sources. Multicentric clinical data sets did not necessarily have more observations than unicentric clinical data sets. On average, multicentre clinical studies had 683 observations, while single-centre studies had 893. Bigger data sets with more than 5,000 observations mainly originate from registries or claims databases (see Fig. 2).

The effect of joint replacement (*n* = 30), spinal surgery (*n* = 24), soft tissue/tendon/ligament surgery (*n* = 18), bony deformity correction (*n* = 11), or physical therapy (*n* = 2) was estimated in the selected studies. The outcome measures included functional outcomes (*n* = 8), revision-related outcomes (*n* = 30), patient-reported outcome measures (PROMs) (*n* = 29), radiographic outcomes (*n* = 8), complications (*n* = 22), hospitalization outcomes (*n* = 4), mortality (*n* = 8), and other outcomes (*n* = 9). Note that the primary outcomes were not necessarily unique in each study. Of the included studies, five had publicly available protocols, while the protocols of 80 studies were not publicly accessible.

Two of the included studies used data provided by CGA. Both focused on children with cerebral palsy undergoing orthopaedic surgery and examined gait-related outcomes. Schwartz *et al.* investigated rectus femoris transfer and applied PSM to compare observational data with results from an RCT. The study found close agreement between the two approaches, supporting the validity of causal inference methods, particularly PSM, for estimating treatment effects on outcomes related to stiff knee gait using CGA data (11). Steele *et al.* evaluated the impact of single-event multi-level orthopaedic surgery on changes in the Gait Deviation Index by constructing a DAG that included the assumed causal relationships among treatment, outcome, and baseline covariates. With the help of the DAG, they could identify an adjustment set and use Bayesian additive regression trees to evaluate the impact of treatment on outcome (21). Together, these studies illustrate two distinct applications of causal inference in neuro-orthopaedics. Despite these contributions, the evidence base remains limited, underscoring the need for further studies that leverage CGA data to estimate causal effects in orthopaedic interventions.

### Causal inference methods

Among the 858 studies included after title and abstract screening, 804 reported the use of PSM, confirming that PSM is the most widely used method of causal inference in the field. However, because of the large number of eligible studies, detailed reporting in this review was limited to a subset of PSM studies that also demonstrated indications of causal intention (see the section titled Methods). The results presented in this section, therefore, reflect this subset, alongside other included studies that employ causal inference approaches.

Propensity score-based causal inference was the most common method across the selected studies, with 38 studies describing the use of IPTW, 31 reporting PSM, 3 employing PS stratification, and 1 utilizing PS adjustment. IV analysis was used in seven studies. At the same time, single uses of other methods, such as potential outcomes simulation, targeted maximum likelihood estimation, causal mediation analysis, or Bayesian additive regression tree, were also observed in the reviewed studies. In some cases, the included studies have used multiple causal inference methods to estimate the effect of the treatment, to check for the robustness of the findings (22, 23, 24).

Among the studies that used IPTW in their main analysis, four applied stabilized weights and none mentioned weight trimming or truncation. PSM was applied 24 times with a one-to-one ratio and 7 times with a one-to-many matching ratio.

Table 2 summarizes the covariates included in propensity score models across the 73 studies estimating a propensity score. Most studies adjusted for patient risk factors, particularly demographics, comorbidities, and baseline function (*n* = 72). In contrast, variables related to surgeon factors were less frequently incorporated (*n* = 4). Among the 47 multicentric studies, 19 included hospital/centre-level factors in their propensity score models. Disease/injury and treatment-related factors were moderately represented, whereas timing variables were included in a minority of studies.

**Table 2:** Covariates used in the propensity score model. Only methods using a propensity score are shown: PSM, IPTW, PS stratification, and PS adjustment. Columns represent covariate categories: patient risk factors (demographics, comorbidities, baseline function, and treatment history), disease/injury factors (diagnosis severity, fracture classification, and revision status), treatment/procedure factors (surgical approach, implant, and concomitant procedures), surgeon factors (surgeon experience and surgeon volume), hospital/centre factors (region and centre volume), and timing factors (year, time to surgery, and urgent vs elective).

	*n*	Patient risk factors	Disease/injury factors	Treatment/procedure factors	Surgeon factors	Hospital/centre factors	Timing factors
Total	73	72	34	23	4	19	17
Centre type							
Multicentric	47	47	22	17	4	19	11
Unicentric	26	25	12	6	0	0	6
TTE							
Yes	3	3	1	2	0	1	1
No	70	69	33	21	4	18	16
Rationale for covariate selection							
DAG	5	5	3	3	0	2	2
Expertise/literature	13	12	6	6	1	2	3
Multivariable analysis	2	2	2	1	0	0	0
Not mentioned	53	53	23	13	3	15	12

The use of TTE remains scarce, with only four studies using this formal approach (see Table 3). Twenty-seven studies provided a rationale for covariate selection in their statistical models, mentioning literature or expertise (*n* = 18), multivariable analysis (*n* = 2), and causal graphs (*n* = 7). No studies described the concurrent use of TTE and causal graph.

**Table 3:** Causal inference methods in relation to the use of centre type, target trials, and rationale for covariate selection. Only the main causal inference method used in each study is reported in this table. For a table including methods used to assess robustness of the findings, see Supplementary Table F.

	Total	PSM	IPTW	PS strat	PS adj	IV	Others
*n*	85	31*	38	3	1	7	5
Centre type							
Multicentric	58	19	24	3	1	7	4
Unicentric	27	12	14	0	0	0	1
TTE							
Yes	4	2	1	0	0	0	1
No	81	29	37	3	1	7	4
Rationale for covariate selection							
DAG	7	3	2	0	0	0	2
Expertise/literature	18	5	8	0	0	5	0
Multivariable analysis	2		1	0	0	0	0
Not mentioned	58	22	27	3	1	2	3

Strat, stratification; adj, adjustment.

* Only a subset of PSM studies (31 out of 804 eligible studies) meeting the review’s inclusion criteria is represented.

**Figure 3:**
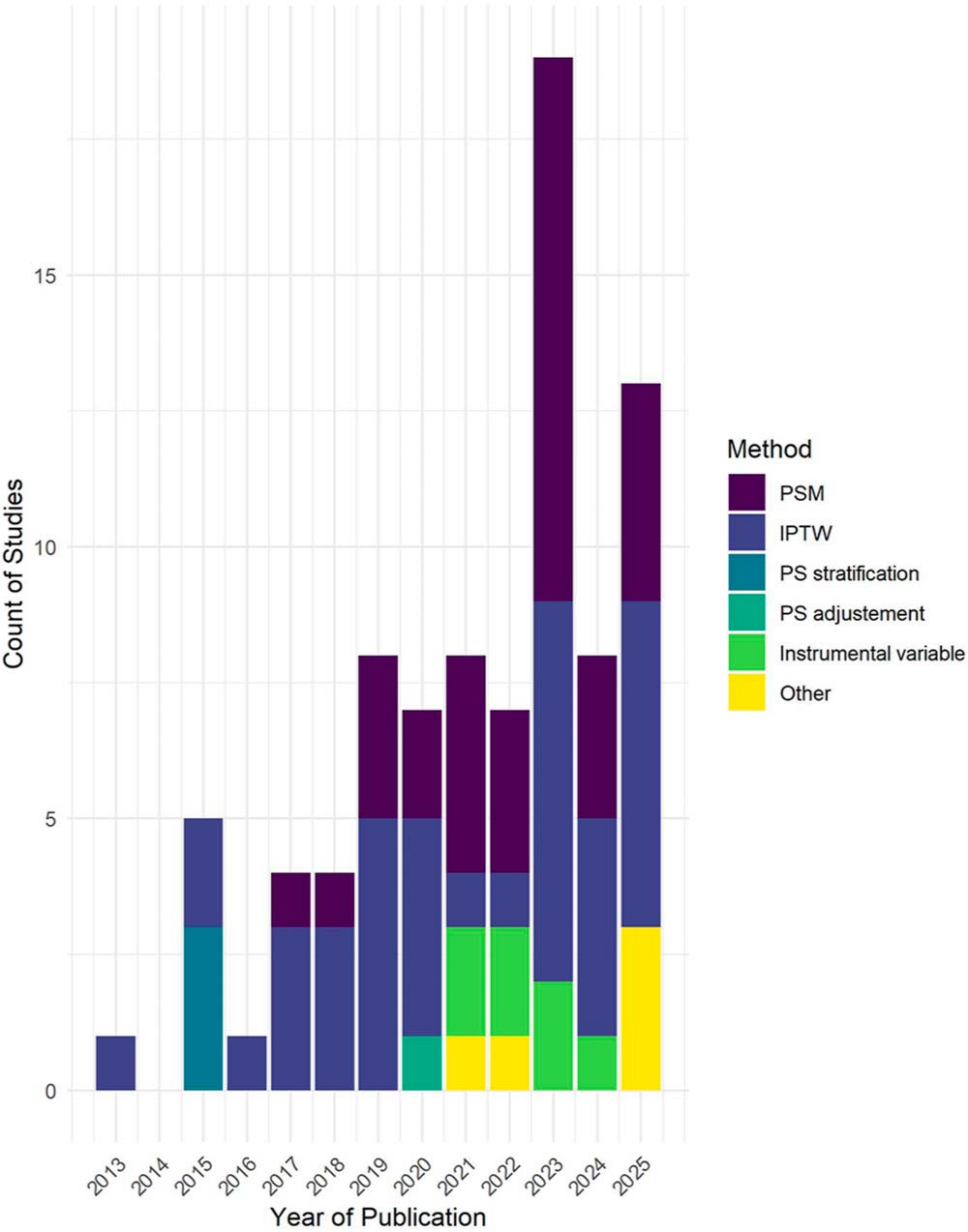
Histogram of causal inference methods by year of publication. Data are shown up to 21 May 2025. Please note that only a subset of PSM studies (31 out of 804 eligible studies) meeting the review’s inclusion criteria is represented. For a figure including methods used to assess robustness of the findings, see Supplementary Fig. A.

The first study included in this review was published in 2013. Since then, the interest in causal inference has grown rapidly in orthopaedics. Between 2023 and 2025, 40 publications met the review’s inclusion criteria (see Fig. 3).

### Assessment of risk of bias

**Figure 4:**
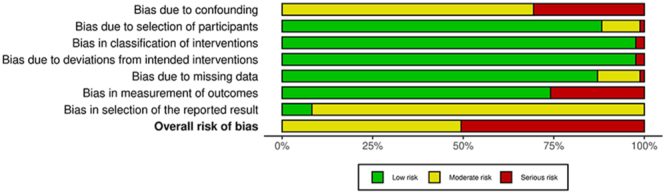
Overall risk of bias from the included studies.

Out of 85 included studies, 42 scored a moderate overall risk score and 43 scored a serious overall risk score (see Supplementary material Tables L and M). Most of the domains showed low risk of bias. Bias due to the confounding domain was at least moderate because confounding was expected in every selected study. Few studies suffered from bias in the selection of participants into the study, because participation in the study may have been related to the intervention and outcome. Most orthopaedic interventions were well defined and based solely on information collected at the time of the intervention. In most cases, deviations from the intended intervention reflected usual practice; therefore, no significant deviations from the planned intervention were reported. The data in the selected studies were, in most cases, complete, or the analyses implemented strategies to deal with missing data. Some critical bias in the measurement of outcomes was observed due to the extensive use of PROMs in orthopaedics. Bias in the selection of the reported results was almost always moderate due to the limited availability of study protocols. The overall risk of bias from the included studies is summarized in Fig. 4.

## Discussion

Our scoping review of 85 studies investigated the use of causal inference methods in orthopaedics. This review shows a marked increase in the use of causal inference in orthopaedics, mainly driven by methods such as PSM and IPTW. These methods enable a more rigorous and innovative use of observational data, helping address clinical questions that cannot be studied through traditional randomized trials. Despite this growth, adoption of more recent formal frameworks, such as TTE and DAGs, remains minimal, limiting transparency and reproducibility.

PSM, utilized in 804 eligible studies to estimate treatment effects, is the most popular causal inference method in orthopaedics. Most of the included PSM studies used a 1:1 matching ratio. The one-to-one ratio tends to minimize bias, and one-to-many ratios are not necessarily superior (25, 26). Inverse probability of the treatment weighting is also relatively frequently adopted in orthopaedic studies. Only 10.5% of the IPTW studies have used stabilized weights, and none have used weight trimming or truncation. Stabilized weights should be preferred over unstabilized weights, as they tend to reduce the variance of the effect estimate (5, 27). In addition, weight stabilization, trimming, and truncation can be used to deal with extreme weights (28). Extreme weights are PS close to 0 or 1, which can occur in studies with large differences in characteristics between the groups.

The fact that multicentric clinical data sets were not necessarily larger than unicentric ones might be surprising. However, in some cases, multicentric studies use small numbers of observations at each investigator’s institution because of the rarity of an event (29) or a condition (30). Even in multicentric settings, the conclusion holds that, in some cases, larger sample sizes are needed for more robust evidence (29, 30). In contrast, some single-centre studies do not need to merge their data with other institutions due to their already large sample size (31, 32). Despite the advantages of multicentre settings, which increase the number of observations and improve the generalizability of the findings, some challenges come with this study design, such as confounding of associations by centre due to differences in exposure prevalence or population demographics across centres (33). IV analysis was only conducted in multicentre settings. Its use was mainly focused on adjusting for the surgeons’ treatment preference (34, 35) or the hospitals’ treatment preferences (36, 37). A recent increase in interest in preference-based IV analysis has also been observed in other fields, such as public health and economics (38).

The scarcity of publicly accessible protocols in medicine is a well-recognized issue in medical research and is not unique to orthopaedics. In contrast to RCTs, there is no strict regulation for observational studies and they can be conducted in minimal time (39, 40). However, prospective publication of protocols for observational studies is encouraged to ensure transparency and avoid changes to the trial after it commenced, such as eligibility criteria or outcomes (41).

A small minority of included studies have used a TTE as a framework for their causal analysis. In addition to confounding bias, observational studies may be subject to bias arising from the misuse of observational data. For example, selection bias refers to an association resulting from the process by which individuals are selected into the analysis. Immortal time bias occurs when follow-up begins before treatment assignment, creating a period during which patients in the treatment group cannot experience the outcome. The objective of TTE is to avoid making fundamental errors that can result in erroneous causal conclusions by emulating, as best as possible, the design of a hypothetical RCT that would answer the same causal question. By mimicking an RCT, TTE helps give rigour to the observational study. The recommendation is to emulate the target trial by carefully addressing each of its protocol components, including eligibility criteria, treatment strategies, treatment assignment, outcomes, the causal estimand, the follow-up period, and the statistical analysis plan (4, 42, 43).

While expert knowledge and the literature can identify variables likely to confound, drawing a DAG provides the added benefit of visually representing the causal relationships between treatment and outcome and informing the statistical analysis. In fact, causal DAGs help us identify a minimal set of variables that, when adjusted for in a statistical analysis, yield valid causal effects. It is standard practice to incorporate a comprehensive set of covariates to prevent the omission of confounding factors. Importantly, the goal of a propensity score model is not to achieve the best prediction of treatment assignment, but rather to balance confounders between treatment groups. Variables that are known or strongly suspected to influence solely the assignment of treatment should be excluded from propensity score models. The inclusion of such variables may diminish the precision of estimates and increase bias if other pertinent variables are disregarded (44, 45, 46). DAGs are a visual tool that helps visualize causal relationships between variables, enabling clear communication among researchers. By providing a detailed DAG, investigators make the assumptions of their analysis more explicit and transparent (4, 47, 48).

Explicit identification of variables by clinical expertise, prior published literature, or the drawing of a causal DAG is essential to ensuring that an analysis can be considered causal. The proportion of studies providing a rationale for confounder selection, or instrument selection in the case of IV analyses, appears similar across studies using PSM, IPTW, or IV methods in the included sample. Nevertheless, because of the way the subset of studies was constructed, it is likely that PSM studies not specifically selected for this analysis have a lower proportion of studies with a clear rationale for confounder selection, and possibly none that employ a DAG. This pattern may also reflect the widespread use of PSM in orthopaedic research, where its popularity as an accessible tool for addressing confounding sometimes leads to its application without a rigorous causal framework or an explicit variable selection strategy.

Propensity score methods have several limitations. First, they rely on the assumption of no unmeasured confounding, as they can only adjust for observed covariates. Second, matching or weighting approaches may alter the target population by restricting analyses to individuals within the region of common support, thereby often estimating the average treatment effect among the treated, rather than the effect in the overall population. PSM has additional practical limitations. It may still be affected by residual confounding, even when no unmeasured confounding is assumed, and typically discards unmatched observations, thereby reducing statistical efficiency. Moreover, PSM may require large sample sizes to achieve adequate matching (4, 46). IV methods offer an alternative approach that can handle unmeasured confounding without relying on the assumption of no unmeasured confounding. However, this comes at the cost of other strong assumptions. In addition, IV methods typically estimate a local average treatment effect in a specific subpopulation, which may limit interpretability and generalizability to a broader population (4, 49).

### Limitations

There are limitations in our study. First, we have deviations from the protocol. We were unable to full-text screen all 858 eligible studies. The use of PSM was overwhelming, and we decided to focus on a subset of studies. We identified only two studies using CGA data, making it difficult to draw conclusions about the use of causal inference methods with CGA. Second, the results from the ROBINS-I tool should be treated cautiously. The tool requires reviewers to assess how well each study controlled for confounding. To judge this properly, reviewers need expertise in identifying the relevant confounders for each type of intervention. Because this scoping review encompassed a wide variety of orthopaedic interventions, it was not feasible to have expert knowledge for each treatment area. As a result, we could only rely on the authors’ judgement regarding confounding adjustment. This led us to penalize authors for identifying potential confounders that might be missing from their models. In many cases, we were unable to assess bias in outcome measurement due to the lack of publicly available protocols.

## Conclusion

Causal inference enables causal estimation when randomization is challenging and supports innovative clinical trial approaches in orthopaedic surgery. Causal inference methods, particularly PSM and IPTW, are increasingly applied to orthopaedic research, but formal frameworks, such as TTE or DAGs, remain underutilized. The study type is multicentric in most cases. Prospective protocols incorporating TTE and DAGs are recommended, along with strategies to address multicentric confounding, to strengthen causal claims and research transparency.

## Supplementary materials



## ICMJE Statement of Interest

The authors declare that there is no conflict of interest that could be perceived as prejudicing the impartiality of the work reported.

## Funding Statement

This work was supported by the Ralf-Loddenkemper Foundation (CH-270.7.002.704-3) and the Swiss Personalized Health Network (SPHN, EVIGAITCP demonstrator project). The funders played no role in the preparation of the manuscript or in the decision to publish.

## Author contribution statement

BW contributed to conceptualization, methodology, data curation, software, investigation, writing of the original draft, and visualization. MK contributed to conceptualization, methodology, validation, data curation, and review and editing. MSi contributed to conceptualization, methodology, and review and editing. GM contributed to conceptualization, methodology, and review and editing. EV contributed to conceptualization, methodology, review and editing, and funding acquisition. MSa contributed to conceptualization, methodology, review and editing, supervision, and funding acquisition.
