# Supplementary material for: Frameworks and methods for estimating the causal effect of clinical treatment in orthopaedics: a scoping review
Source: EFORT Open Rev. 2026 Jul 1;11(7):748–57. doi: 10.1530/EOR-2025-0266 (PMC13326879; doi:10.1530/EOR-2025-0266)
Supplement: Supplementary file 1 [file supplementary_materials.pdf]

Supplementary Material

Table A
PRISMA Checklist

SECTION	ITEM	PRISMA-ScR CHECKLIST ITEM	REPORTED ON PAGE #
**TITLE**			
Title	1	Identify the report as a scoping review.	1
**ABSTRACT**			
Structured summary	2	Provide a structured summary that includes (as applicable): background, objectives, eligibility criteria, sources of evidence, charting methods, results, and conclusions that relate to the review questions and objectives.	1-2
**INTRODUCTION**			
Rationale	3	Describe the rationale for the review in the context of what is already known. Explain why the review questions/objectives lend themselves to a scoping review approach.	2-3
Objectives	4	Provide an explicit statement of the questions and objectives being addressed with reference to their key elements (e.g., population or participants, concepts, and context) or other relevant key elements used to conceptualize the review questions and/or objectives.	3
**METHODS**			
Protocol and registration	5	Indicate whether a review protocol exists; state if and where it can be accessed (e.g., a Web address); and if available, provide registration information, including the registration number.	4
Eligibility criteria	6	Specify characteristics of the sources of evidence used as eligibility criteria (e.g., years considered, language, and publication status), and provide a rationale.	4-5,<br>Supplementary Table B
Information sources*	7	Describe all information sources in the search (e.g., databases with dates of coverage and contact with authors to identify additional sources), as well as the date the most recent search was executed.	4
Search	8	Present the full electronic search strategy for at least 1 database, including any limits used, such that it could be repeated.	Supplementary Table D
Selection of sources of evidence†	9	State the process for selecting sources of evidence (i.e., screening and eligibility) included in the scoping review.	4-5
Data charting process‡	10	Describe the methods of charting data from the included sources of evidence (e.g., calibrated forms or forms that have been tested by the team before their use, and whether data charting was done independently or in duplicate) and any processes for obtaining and confirming data from investigators.	5
Data items	11	List and define all variables for which data were sought and any assumptions and simplifications made.	Supplementary Table C
Critical appraisal of individual sources of evidence§	12	If done, provide a rationale for conducting a critical appraisal of included sources of evidence; describe the methods used and how this information was used in any data synthesis (if appropriate).	5
Synthesis of results	13	Describe the methods of handling and summarizing the data that were charted.	5-6
**RESULTS**			
Selection of sources of evidence	14	Give numbers of sources of evidence screened, assessed for eligibility, and included in the review, with reasons for exclusions at each stage, ideally using a flow diagram.	6-7
Characteristics of sources of evidence	15	For each source of evidence, present characteristics for which data were charted and provide the citations.	Supplementary Table G,<br>Supplementary references
Critical appraisal within sources of evidence	16	If done, present data on critical appraisal of included sources of evidence (see item 12).	12-13
Results of individual sources of evidence	17	For each included source of evidence, present the relevant data that were charted that relate to the review questions and objectives.	7-12,<br>Supplementary Table G
Synthesis of results	18	Summarize and/or present the charting results as they relate to the review questions and objectives.	7-13
**DISCUSSION**			
Summary of evidence	19	Summarize the main results (including an overview of concepts, themes, and types of evidence available), link to the review questions and objectives, and consider the relevance to key groups.	13-15
Limitations	20	Discuss the limitations of the scoping review process.	15-16
Conclusions	21	Provide a general interpretation of the results with respect to the review questions and objectives, as well as potential implications and/or next steps.	16
**FUNDING**			

Table A
PRISMA Checklist

SECTION	ITEM	PRISMA-ScR CHECKLIST ITEM	REPORTED ON PAGE #
Funding	22	Describe sources of funding for the included sources of evidence, as well as sources of funding for the scoping review. Describe the role of the funders of the scoping review.	16-17

Table B

Population, concept, context (PCC) framework, and types of evidence for identifying the main concepts within the research question.

PCC Element	Inclusion	Exclusion
Population	Human patients subject to an orthopaedic clinical treatment, which can be orthopaedic surgery, conservative treatment with orthotics, physiotherapy, or occupational therapy.	Animals
Concept	Studies that describe the use of a causal inference framework to assess effects of an orthopaedic treatment.	Studies estimating a causal effect using RCT only
Context	Any geographical location or setting of any nature	None
Types of evidence	Observational studies (eg, cohort studies and case-control studies in which intervention groups are allocated during the course of usual treatment decisions, and quasi-randomised studies in which the method of allocation falls short of full randomisation), full-text articles	Reviews (eg, systematic reviews, narrative reviews)

Table C

Charting table following the Joanna Briggs Institute's template for data extraction.

Dimensions	Details
Citation details	Author/s, Year of publication, Title, Country
Inclusion/Exclusion Criteria	Population, Concept, Context, Types of evidence source
Evidence source Details and Characteristics	Aims/purpose, Participants (details e.g. age/sex and number), Intervention type, Comparator and details of these (e.g. duration of the intervention), Outcomes and details of these (e.g. how measured)
Details/Results extracted from source of evidence	Causal inference methods used, Use of target trial, Use of causal graphs, Centre type (unicentric or multicentric), Rationale for confounders selection, Published protocol, Clinical gait analysis

Table D

Pubmed search string

	Stream (Pubmed)
A	("orthopedic procedures"[MeSH Terms]) OR (("orthopaedic*"[Title/Abstract] OR "orthopedic*"[Title/Abstract]) AND ("surger*"[Title/Abstract] OR "surgical*"[Title/Abstract] OR "intervention*"[Title/Abstract] OR "procedure*"[Title/Abstract]))
B	("causal effect*"[Title/Abstract] OR "causal inference*"[Title/Abstract] OR "Propensity Score"[MeSH Terms] OR "propensity score*"[Title/Abstract] OR "instrumental var*"[Title/Abstract] OR "directed acyclic graph*"[Title/Abstract] OR "inverse probability weighting*"[Title/Abstract] OR "trial emulation*"[Title/Abstract] OR "g method*"[Title/Abstract] OR "g formula*"[Title/Abstract] OR "g computation*"[Title/Abstract])
Combination	(("orthopedic procedures"[MeSH Terms]) OR (("orthopaedic*"[Title/Abstract] OR "orthopedic*"[Title/Abstract])

	AND ("surger*"[Title/Abstract] OR "surgical*"[Title/Abstract] OR "intervention*"[Title/Abstract] OR "procedure*"[Title/Abstract]))) AND ("Causal Effect*"[Title/Abstract] OR "Causal Inference*"[Title/Abstract] OR "Propensity Score"[MeSH Terms] OR "propensity score*"[Title/Abstract] OR "instrumental var*"[Title/Abstract] OR "directed acyclic graph*"[Title/Abstract] OR "inverse probability weighting*"[Title/Abstract] OR "trial emulation*"[Title/Abstract] OR "g-method*"[Title/Abstract] OR "g-formula*"[Title/Abstract] OR "g-computation*"[Title/Abstract])

Table E

List of keywords used to retrieve a subset of full-text PSM studies.

Topic	Keywords
DAG	DAG, directed acyclic graph, causal graph, causal diagram
Protocol	study protocol, publicly available at, github
Target trial emulation	target trial, emulation
Variable selection	confounder selection, variable selection
Causal intention	effect, causal inference

Table F

Causal inference methods, including approaches used to assess robustness of findings, in relation to the use of target trials, centre type, and use of causal graphs.

	Total (n=85)	PSM (n=32[a])	IPTW (n=48)	PS stratification (n=5)	PS adjustment (n=2)	IV (n=9)	Other (n=5)
Centre type							
Multicentric	58	20	32	5	2	9	4
Unicentric	27	12	16	0	0	0	2
TTE							
Yes	4	2	2	1	1	1	0
No	81	30	46	4	1	8	5
Rationale for covariate selection							
DAG	7	3	3	1	0	0	2
Expertise/Literature	18	5	9	0	0	5	1
Multivariable analysis	2	1	2	0	0	0	0
Not mentioned	58	23	34	4	2	4	2

Note. The sum of the methods used is not equal to the total column. Some studies employed multiple techniques, e.g., for sensitivity analysis.
[a]Please note that only a subset of PSM studies (31 out of 804 eligible studies) meeting the review's inclusion criteria is represented.

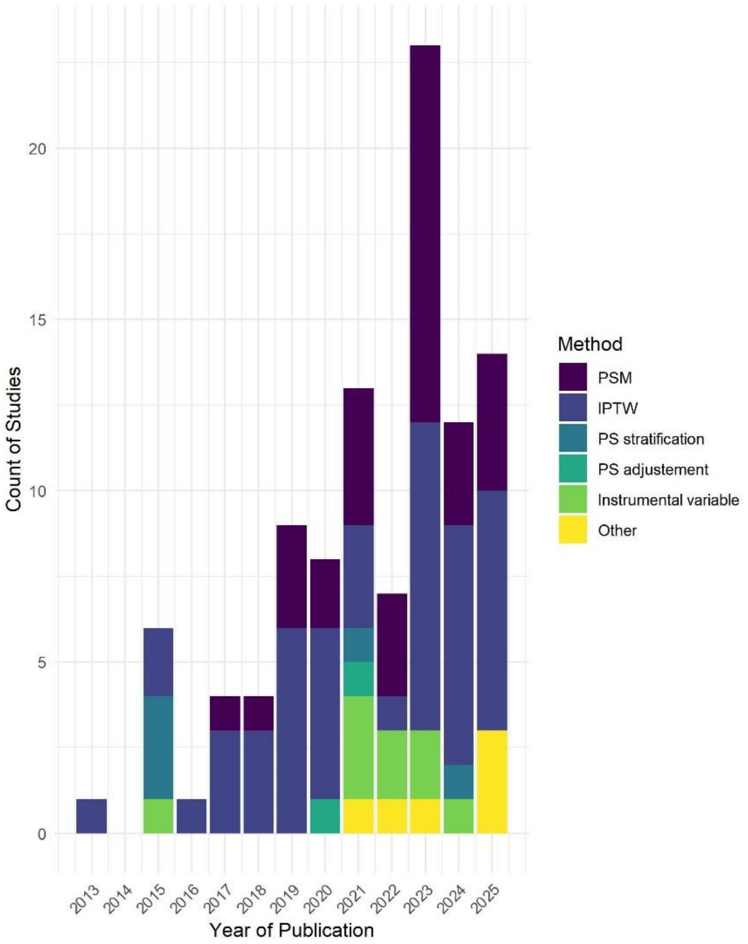

Fig. A. Histogram of causal inference methods by year of publication, including approaches used to assess robustness of findings. Data are shown up to May 21, 2025. Please note that only a subset of PSM studies (31 out of 804 eligible studies) meeting the review's inclusion criteria are represented

Table G
Citation details charting table

ID	Authors	Year of Publication	Title	Country
1	Schwartz MH, Ries AJ.	2021	Rectus femoris transfer in children with cerebral palsy: comparing a propensity score-matched observational study to a randomized controlled trial	USA
2	Steele KM, Schwartz MH.	2022	Causal Effects of Motor Control on Gait Kinematics After Orthopedic Surgery in Cerebral Palsy: A Machine-Learning Approach	USA
3	Edelstein AI, Dillingham TR, McGinley EL, Pezzin LE	2023	Hemiarthroplasty Versus Total Hip Arthroplasty for Femoral Neck Fracture in Elderly Patients: Twelve-Month Risk of Revision and Dislocation in an Instrumental Variable Analysis of Medicare Data	USA
4	Yoon JR, Ko SN, Jung KY, Lee Y, Park JO, Shin YS.	2019	Risk of Revision Following Total Knee Arthroplasty or High Tibial Osteotomy: A Nationwide Propensity-Score-Matched Study	South Korea
5	Sawaguchi A, Momosaki R, Hasebe K, Chono M, Kasuga S, Abo M	2018	Effectiveness of preoperative physical therapy for older patients with hip fracture	Japan
6	Nugent M, Campbell DG, Lewis PL, Cuthbert AR, Solomon LB	2021	Acetabular screws do not improve early revision rates in primary total hip arthroplasty. An instrumented registry analysis	Australia
7	Latijnhouwers DAJM, Laas N, Verdegaal SHM, Nelissen RGHH, Vliet Vlieland TPM, Kaptijn HH, Gademan MGJ; Longitudinal Leiden Orthopaedics Outcomes of Osteoarthritis Study (LOAS) Group	2022	Activities and participation after primary total hip arthroplasty; posterolateral versus direct anterior approach in 860 patients	Netherlands
8	Inacio MC, Cafri G, Paxton EW, Kurtz SM, Namba RS	2013	Alternative bearings in total knee arthroplasty: risk of early revision compared to traditional bearings: an analysis of 62,177 primary cases	USA
9	Egbert RC, Chan PH, Chan DP, Prentice HA, McElvany MD, Yian EH	2023	Antibiotic-loaded bone cement vs. plain cement as an infection prophylaxis in primary elective shoulder arthroplasty	USA
10	Masuda S, Fujibayashi S, Takemoto M, Ota M, Onishi E, Odate S, Tsutumi R, Izeki M, Kimura H, Tanida S, Otsuki B, Murata K, Shimizu T, Matsuda S	2023	Association of Two-Staged Surgery with Systemic Perioperative Complications in Lateral Lumbar Interbody Fusion for Adult Spinal Deformity: A Propensity Score-Weighted Study.	Japan
11	Farey JE, Cuthbert AR, Adie S, Harris IA	2022	Bipolar Hemiarthroplasty Does Not Result in a Higher Risk of Revision Compared with Total Hip Arthroplasty for Displaced Femoral Neck Fractures: An Instrumental Variable Analysis of 36,118 Procedures from the Australian Orthopaedic Association National Joint Replacement Registry.	Australia
12	Takenaka S, Mukai Y, Tateishi K, Hosono N, Fuji T, Kaito T	2017	Clinical Outcomes After Posterior Lumbar Interbody Fusion: Comparison of Cortical Bone Trajectory and Conventional Pedicle Screw Insertion.	Japan
13	Zhou C, Selles RW, Slijper HP, Feitz R, van Kooij Y, Moojen TM, Hovius SER	2016	Comparative Effectiveness of Percutaneous Needle Aponeurotomy and Limited Fasciectomy for Dupuytren's Contracture: A Multicenter Observational Study.	Netherlands
14	Sugiura T, Okuda S, Takenaka S, Nagamoto Y, Matsumoto T, Takahashi Y, Iwasaki M	2021	Comparing Investigation Between Bilateral Partial Laminectomy and Posterior Lumbar Interbody Fusion for Mild Degenerative Spondylolisthesis.	Japan
15	Trisolino G, Stilli S, Gallone G, Santos Leite P, Pignatti G	2018	Comparison between modified Dunn procedure and in situ fixation for severe stable slipped capital femoral epiphysis	Italy
16	Huang KT, Hazzard M, Thomas S, Chagoya G, Berg RW, Adogwa O, Bagley CA, Isaacs R, Gottfried ON, Lad SP	2015	Differences in the outcomes of anterior versus posterior interbody fusion surgery of the lumbar spine: a propensity score-controlled cohort analysis of 10,941 patients.	USA
17	Takashima K, Sakai T, Amano S, Hamada H, Ando W, Takao M, Hamasaki T, Nakamura N, Sugano N	2020	Does a computed tomography-based navigation system reduce the risk of dislocation after total hip arthroplasty in patients with osteonecrosis of the femoral head? A propensity score analysis.	Japan
18	Häberli J, Bieri KS, Aghayev E, Eggli S, Henle P	2019	Dynamic intraligamentary stabilization of anterior cruciate ligament repair: hardware removal has no effect on knee laxity at 2-year follow-up.	Switzerland
19	Lecoq FA, Parienti JJ, Murison J, Ruiz N, Bouacida K, Besse J,	2018	Graft Choice and the Incidence of Osteoarthritis After Anterior Cruciate Ligament Reconstruction: A Causal	France

	Morin V, Padiolleau G, Cucurulo T, Graveleau N, Hulet C		Analysis From a Cohort of 541 Patients.	
20	Leitner L, Bratschitsch G, Kostwein A, Sadoghi P, Smolle M, Leithner A, Posch F	2023	More help than harm: surgery for metastatic spinal cord compression is associated with more favorable overall survival within a propensity score analysis	Austria
21	Major Extremity Trauma Research Consortium (METRC)	2021	Outcomes Following Severe Distal Tibial, Ankle, and/or Mid/Hindfoot Trauma: Comparison of Limb Salvage and Transtibial Amputation (OUTLET).	USA
22	Munting E, Röder C, Sobottke R, Dietrich D, Aghayev E	2015	Patient outcomes after laminotomy, hemilaminectomy, laminectomy and laminectomy with instrumented fusion for spinal canal stenosis: a propensity score-based study from the Spine Tango registry.	Belgium
23	Lin WY, Lee CC, Hsu CW, Huang KY, Lyu SR	2015	Patients with knee osteoarthritis undergoing total knee arthroplasty have a lower risk of subsequent severe cardiovascular events: propensity score and instrumental variable analysis.	Taiwan
24	Katz JN, Collins JE, Brophy RH, Cole BJ, Cox CL, Guermazi A, Jones MH, Levy BA, MacFarlane LA, Mandl LA, Marx RG, Selzer F, Spindler KP, Wright RW, Losina E, Chang Y	2023	Radiographic Changes Five Years After Treatment of Meniscal Tear and Osteoarthritic Changes	USA
25	Farey JE, Cuthbert AR, Adie S, Harris IA	2021	Revision Risk After Unipolar or Bipolar Hemiarthroplasty for Femoral Neck Fractures: An Instrumental Variable Analysis of 62,875 Procedures from the Australian Orthopaedic Association National Joint Replacement Registry.	Australia
26	Yoo JD, Huh MH, Shin YS	2023	Risk of revision in UKA versus HTO: a nationwide propensity score-matched study.	South Korea
27	Bendich I, Vigdorchik JM, Sharma AK, Mayman DJ, Sculco PK, Anderson C, Della Valle AG, Su EP, Jerabek SA	2022	Robotic Assistance for Posterior Approach Total Hip Arthroplasty Is Associated With Lower Risk of Revision for Dislocation When Compared to Manual Techniques	USA
28	Reingrittha P, Benjawongsathien K, Visuthisakchai S	2023	The Efficacy of Posterior Fasciotomy Versus Inserted Vacuum Drainage in Reducing Postoperative Surgical Site Infection in Open Achilles Tendon Repair: A Prospective Cohort Study With Inverse Probability Treatment Weight Propensity Score Analysis	Thailand
29	Ramsay N, Close JCT, Harris IA, Harvey LA	2023	The impact of fixation type for intertrochanteric femoral fracture on patient survival.	Australia
30	Prats-Uribe A, Kolovos S, Berencsi K, Carr A, Judge A, Silman A, Arden N, Petersen I, Douglas IJ, Wilkinson JM, Murray D, Valderas JM, Beard DJ, Lamb SE, Ali MS, Pinedo-Villanueva R, Strauss VY, Prieto-Alhambra D	2021	Unicompartmental compared with total knee replacement for patients with multimorbidities: a cohort study using propensity score stratification and inverse probability weighting.	UK
31	Jain NB, Ayers GD, Fan R, Kuhn JE, Warner JJP, Baumgarten KM, Matzkin E, Higgins LD	2019	Comparative Effectiveness of Operative Versus Nonoperative Treatment for Rotator Cuff Tears: A Propensity Score Analysis From the ROW Cohort.	USA
32	Paxton EW, Inacio MC, Kurtz S, Love R, Cafri G, Namba RS	2015	Is there a difference in total knee arthroplasty risk of revision in highly crosslinked versus conventional polyethylene?	USA
33	Paxton EW, Inacio MC, Namba RS, Love R, Kurtz SM	2015	Metal-on-conventional polyethylene total hip arthroplasty bearing surfaces have a higher risk of revision than metal-on-highly crosslinked polyethylene: results from a US registry.	USA
34	Millett PJ, Espinoza C, Horan MP, Ho CP, Warth RJ, Dornan GJ, Katthagen JC	2017	Predictors of outcomes after arthroscopic transosseous equivalent rotator cuff repair in 155 cases: a propensity score weighted analysis of knotted and knotless self-reinforcing repair techniques at a minimum of 2 years.	USA
35	El-Galaly A, Nielsen PT, Kappel A, Jensen SL	2020	Reduced survival of total knee arthroplasty after previous unicompartmental knee arthroplasty compared with previous high tibial osteotomy: a propensity-score weighted mid-term cohort study based on 2,133 observations from the Danish Knee Arthroplasty Registry.	Denmark
36	Baumbach SF, Urresti-Gundlach M, Braunstein M, Borgmann L, Böcker W, Vosseller JT, Polzer H	2021	Propensity Score-Matched Analysis of Arthroscopically Assisted Ankle Facture Treatment Versus Conventional Treatment	Germany
37	Rupp MC, Lindner F, Winkler PW, Muench LN, Mehl J, Imhoff AB, Siebenlist S, Feucht MJ	2023	Clinical Effect of Isolated Lateral Closing Wedge Distal Femoral Osteotomy Compared to Medial Opening Wedge High Tibial Osteotomy for the	Germany

			Correction of Varus Malalignment: A Propensity Score-Matched Analysis	
38	Yang P, He R, Lei K, Liu L, Yang L, Guo L	2023	Clinical evaluation of the first semi-active total knee arthroplasty assisting robot made in China: a retrospective propensity score-matched cohort study.	China
39	Rongen JJ, Rovers MM, van Tienen TG, Buma P, Hannink G	2017	Increased risk for knee replacement surgery after arthroscopic surgery for degenerative meniscal tears: a multi-center longitudinal observational study using data from the osteoarthritis initiative.	Netherlands
40	Han SB, Song SY, Shim JH, Shin YS	2021	Risk of a complete exchange or failure in total knee arthroplasty and unicompartmental knee arthroplasty: a nationwide population-based cohort study from South Korea.	South Korea
41	MacDowall A, Skeppholm M, Lindhagen L, Robinson Y, Löfgren H, Michaëlsson K, Olerud C	2018	Artificial disc replacement versus fusion in patients with cervical degenerative disc disease with radiculopathy: 5-year outcomes from the National Swedish Spine Register	Sweden
42	Jevotovsky DS, Thirukumaran CP, Rubery PT	2019	Creating value in spine surgery: using patient reported outcomes to compare the short-term impact of different orthopedic surgical procedures.	USA
43	Zelenty WD, Paek S, Dodo Y, Sarin M, Shue J, Soffin E, Lebl DR, Cammisa FP, Girardi FP, Sokunbi G, Sama AA, Hughes AP	2023	Utilization Trends of Intraoperative Neuromonitoring for Anterior Cervical Discectomy and Fusion in New York State	USA
44	Hahn HM, Cook KH, Lee IJ, Park DH, Park MC	2017	Use of Acellular Dermal Matrix in Treatment of Congenital Muscular Torticollis in Patients Over Eight Years of Age.	South Korea
45	Kudo Y, Okano I, Toyone T, Matsuoka A, Maruyama H, Yamamura R, Ishikawa K, Hayakawa C, Tani S, Sekimizu M, Hoshino Y, Ozawa T, Shirahata T, Fujita M, Oshita Y, Emori H, Omata H, Inagaki K	2020	Lateral lumbar interbody fusion in revision surgery for restenosis after posterior decompression.	Japan
46	Gonzalez GA, Corso K, Kothari P, Franco D, Porto G, Miao J, Wainwright JV, O'Leary M, Hines K, Mahtabfar A, Vanderkarr M, Thalheimer S, Sharan A, Jallo J, Harrop J	2023	Lumbar Synovial Cysts-Should You Fuse or Not?	USA
47	Ioannidis I, Mohammad Ismail A, Forssten MP, Ahl R, Cao Y, Borg T, Mohseni S	2022	Surgical management of displaced femoral neck fractures in patients with dementia: a comparison in mortality between hemiarthroplasty and pins/screws.	Sweden
48	Cloney MB, Hopkins B, Dhillon E, El Tecle N, Swong K, Koski TR, Dahdaleh NS	2022	Anterior approach lumbar fusions cause a marked increase in thromboembolic events: Causal inferences from a propensity-matched analysis of 1147 patients	USA
49	Namba RS, Prentice HA, Paxton EW, Hinman AD, Kelly MP	2020	Commercially Prepared Antibiotic-Loaded Bone Cement and Infection Risk Following Cemented Primary Total Knee Arthroplasty	USA
50	Hernandez NM, Hart A, Taunton MJ, Osmon DR, Mabry TM, Abdel MP, Perry KI	2019	Use of Povidone-Iodine Irrigation Prior to Wound Closure in Primary Total Hip and Knee Arthroplasty: An Analysis of 11,738 Cases	USA
51	Honda A, Iizuka Y, Michihata N, Morita K, Mieda T, Takasawa E, Ishiwata S, Kakuta Y, Tomomatsu Y, Ito S, Inomata K, Matsui H, Fushimi K, Yasunaga H, Chikuda H	2023	Risk factors for early mortality in elderly patients with unstable isolated C2 odontoid fracture treated with halo-vest or surgery	Japan
52	Masuda S, Fukasawa T, Takeuchi M, Fujibayashi S, Otsuki B, Murata K, Shimizu T, Matsuda S, Kawakami K	2023	Reoperation Rates of Microendoscopic Discectomy Compared With Conventional Open Lumbar Discectomy: A Large-database Study.	Japan
53	Hoepelman RJ, Beeres FJP, Beks RB, Sweet AAR, Ijpma FF, Lansink KWW, van Wageningen B, Tromp TN, Link BC, van Veelen NM, Hoogendoorn JM, de Jong MB, van Baal MCP, Leenen LPH, Groenwold RHH, Houwert RM	2023	Non-operative vs. operative treatment for multiple rib fractures after blunt thoracic trauma: a multicenter prospective cohort study	Netherlands
54	Silva PS, Jardim A, Pereira J, Sousa R, Vaz R, Pereira P	2023	Minimally invasive fusion surgery for patients with degenerative spondylolisthesis and severe lumbar spinal stenosis: a comparative study between MIDLIF and TLIF	Portugal
55	Ramme AJ, Robbins CB, Patel KA,	2019	Surgical Versus Nonsurgical Management of Rotator	USA

	Carpenter JE, Bedi A, Gagnier JJ, Miller BS		Cuff Tears: A Matched-Pair Analysis	
56	Mohanty S, Barchick S, Kadiyala M, Lad M, Rouhi AD, Vadali C, Albayar A, Ozturk AK, Khalsa A, Saifi C, Casper DS	2023	Should patients with lumbar stenosis and grade I spondylolisthesis be treated differently based on spinopelvic alignment? A retrospective, two-year, propensity matched, comparison of patient-reported outcome measures and clinical outcomes from multiple sites within a single health system	USA
57	Chan, R.W.-Y., Chiang, Y.-H., Lin, H.-C., Chang, C.-Y., Tsou, Y.-S.	2023	Postoperative 30-Day Comparative Complications of Multilevel Anterior Cervical Discectomy and Fusion and Laminoplasty for Cervical Spondylotic Myelopathy: An Evidence in Reaching Consensus	Taiwan
58	Mori, Y., Takegami, Y., Tokutake, K., Oka, Y., Imagama, S.	2023	Retrospective Comparative Study of Clinical Outcomes and Cost-Effectiveness with Bone Substitutes on Volar Locking Plate Fixation of Unstable Distal Radial Fractures in the Elderly	Japan
59	Wernecke, G.C., Jin, X., Lin, J.-L., Harris, I.A.	2024	The Impact of Surgical Approach on 90-Day Prosthetic Joint Infection After Total Hip Replacement — A Population-Based, Propensity Score-Matched Cohort Study	Australia
60	Hardwick-Morris, M., Carlton, S., Twiggs, J., Miles, B., Liu, D.	2022	Pre- and postoperative physiotherapy using a digital application decreases length of stay without reducing patient outcomes following total knee arthroplasty	Australia
61	Ogawa, T., Yoshii, T., Moriwaki, M., Morishita, S., Oh, Y., Miyatake, K., Nazarian, A., Shiba, K., Okawa, A., Fushimi, K., Fujiwara, T.	2020	Association between Hemiarthroplasty vs. Total Hip Arthroplasty and Major Surgical Complications among Patients with Femoral Neck Fracture	Japan
62	Catalino, M.P., Pate, V., Stürmer, T., Bhowmick, D.A.	2020	Comparative Propensity-Weighted Mortality After Isolated Acute Traumatic Axis Fractures in Older Adults	USA
63	MacDowall, A., Heary, R.F., Holy, M., Lindhagen, L., Olerud, C.	2020	Posterior foraminotomy versus anterior decompression and fusion in patients with cervical degenerative disc disease with radiculopathy: Up to 5 years of outcome from the national Swedish Spine Register	Sweden
64	Burn, E., Weaver, J., Morales, D., Prats-Uribe, A., Delmestri, A., Strauss, V.Y., He, Y., Robinson, D.E., Pinedo-Villanueva, R., Kolovos, S., Duarte-Salles, T., Sproviero, W., Yu, D., Van Speybroeck, M., Williams, R., John, L.H., Hughes, N., Sena, A.G., Costello, R., Birlie, B., Culliford, D., O'Leary, C., Morgan, H., Burkard, T., Prieto-Alhambra, D., Ryan, P.	2019	Opioid use, postoperative complications, and implant survival after unicompartmental versus total knee replacement: a population-based network study	USA (UK)
65	Canouï, E., Zarrouk, V., Canouï-Poitrine, F., Desmoulin, U., Leflon, V., Allaham, W., de Lastours, V., Guigui, P., Fantin, B.	2019	Surgery is safe and effective when indicated in the acute phase of hematogenous pyogenic vertebral osteomyelitis	France
66	Mahamid, A., Laver, L., Maman, D., Abu Elhija, A., Haj Yahya, M., Haverkamp, D., Berkovich, Y., Behrbalk, E.	2025	Comparing Early Outcomes and Complications Between Total Ankle Arthroplasty and Ankle Arthrodesis in Patients with Ankle Osteoarthritis: Big Data Analysis	Israel
67	Farhan-Alanie, M.M., Gallacher, D., Craig, P., Griffin, J., Kozdryk, J., Mason, J., Wall, P.D.H., Wilkinson, J.M., Metcalfe, A., Foguet, P.	2025	The Effects of Computer Navigation and Patient-Specific Instrumentation on Risk of Revision, PROMs, and Mortality Following Primary TKR: An Analysis of National Joint Registry Data	UK
68	Chundi, G., Dawar, A., Ahn, D.B., Chopra, A.A., Joshi, T., Lin, S.S., Jones, T.	2025	Use of Sustained Compression to Mitigate Nonunion in Tibiotalocalcaneal Arthrodesis: A Propensity Score-Matched Nationwide Readmissions Database Analysis	USA
69	Lu, Y., Jurgensmeier, K., Lamba, A., Yang, L., Hevesi, M., Camp, C.L., Krych, A.J., Stuart, M.J.	2025	Posttraumatic Arthritis After Anterior Cruciate Ligament Injury: Machine Learning Comparison Between Surgery and Nonoperative Management	USA
70	Bendich, I., Chiu, Y.-F., Sarpong, N., Gonzalez Della Valle, A., Su, E., McLawhorn, A.	2025	Robotic-assistance and computer-navigation have similar rates of intraoperative fracture and return to the operating room within 1 year to fluoroscopy-only direct anterior total hip arthroplasty	USA
71	Mohanty, S., Stephan, S.R., Mikhail, C., Platt, A., Bakhsheshian, J., Hassan, F.M.,	2025	Maintaining stability at the lumbosacral-pelvic region in adult spinal deformity surgery without sacroiliac joint fusion: are 4 pelvic screws superior to 2 pelvic	USA

	Lewerenz, E., Lombardi, J.M., Sardar, Z.M., Lehman, R.A., Lenke, L.G.		screws?	
72	Sheean, A.J., Jin, Y., Amendola, A., Huston, L.J., Brophy, R.H., Cox, C.L., Jones, M.H., Kaeding, C.C., Kattan, M.W., Magnussen, R.A., Marx, R.G., Matava, M.J., McCarty, E.C., Parker, R.D., Reinke, E., Wolcott, M.L., Wolf, B.R., Wright, R.W., Spindler, K.P.	2025	Successful Medial Meniscal Repair Reduces Knee Pain 10 Years After Anterior Cruciate Ligament Reconstruction: Exploring the Consequences of Subsequent Surgery With Causal Mediation Analysis in the MOON Cohort	USA
73	Biz, C., Bragazzi, N.L., Di Rita, A., Pozzuoli, A., Belluzzi, E., Rodà, M.G., Ruggieri, P	2025	Comparative analysis between Reverdin-Isham Osteotomy (RIO) and minimally invasive intramedullary nail device (MIIND) in association with AKIN osteotomy for Hallux valgus correction	Italy
74	Mirzayan, R., Chang, R.N., Royse, K.E., Reyes, C.E., Prentice, H.A., Maletis, G.B.	2025	Is There a Hamstring Autograft Diameter Threshold for Anterior Cruciate Ligament Reconstruction?	USA
75	Ozdag, Y., Koshinski, J.L., Hayes, D.S., Cornwell, D., Garcia, V.C., Klena, J.C., Grandizio, L.C.	2025	Early Rates of Revision Surgery in Endoscopic and Open Carpal Tunnel Release	USA
76	Klaassen, A.D., Jorritsma, W., Willigenburg, N.W., Gerritsma, C.L.E., Have, B.L.E.F.T., Moojen, D.J.F., Gademan, M.G.J., Groenwold, R.H.H., Poolman, R.W.	2025	Effectiveness of total hip arthroplasty versus non-surgery on patient-reported hip function at 3 months: a target trial emulation study of patients with osteoarthritis	Netherlands
77	Chen, F., Chang, R.N., Prentice, H.A., Fasig, B.H., Paxton, E.W., Hug, K.T., Kelly, M.P.	2025	What Is the Survivorship of TKA With a Twin-peg or Spikes-and-keel Cementless Implant Compared With Cemented? A Registry-based Cohort Study	USA
78	Hinman, A.D., Chang, R.N., Kelly, M.P., Fasig, B.H., Paxton, E.W., Royse, K.E.	2025	Impact of Metaphyseal Sleeves and Porous Cones on Risk Reduction for Revision Total Knee Arthroplasty: Aseptic Re-Revision in a United States Integrated Health Care System	USA
79	Unterfrauner, I., Muñoz Laguna, J., Serra-Burriel, M., Burgstaller, J.M., Uçkay, I., Farshad, M., Hincapié, C.A.	2024	Fusion versus decompression alone for lumbar degenerative spondylolisthesis and spinal stenosis: a target trial emulation with index trial benchmarking	Switzerland
80	Dornan, G.J., Ruzbarsky, J.J., Comfort, S.M., Ernat, J.J., Martin, M.D., Briggs, K.K., Philippon, M.J.	2024	Two-Year Outcomes of Primary Arthroscopic Surgery in Patients with Femoroacetabular Impingement A Comparative Study of Labral Repair and Labral Reconstruction	USA
81	Hatano, M., Sasabuchi, Y., Isogai, T., Yasunaga, H., Ishikura, H., Tanaka, T., Tanaka, S.	2024	Increased early complications after total hip arthroplasty compared with hemiarthroplasty in older adults with a femoral neck fracture A NATIONWIDE RETROSPECTIVE COHORT STUDY	Japan
82	Subramanian, A., Adejuyigbe, B., Niknam, K., Gomez-Alvarado, F., Morshed, S., Shearer, D.	2024	Retrospective cohort study analyzing outcomes of the SIGN Fin Nail in adult femoral fractures using the retrograde approach	USA
83	Yamato, Y., Nagata, K., Kawamura, N., Higashikawa, A., Takeshita, Y., Tozawa, K., Fukushima, M., Urayama, D., Ono, T., Hara, N., Okamoto, N., Azuma, S., Iwai, H., Sugita, S., Yoshida, Y., Hirai, S., Masuda, K., Jim, Y., Ohtomo, N., Nakamoto, H., Kato, S., Taniguchi, Y., Tanaka, S., Oshima, Y.	2024	Comparative Analysis of Microendoscopic and Open Laminectomy for Single-Level Lumbar Spinal Stenosis at L1–L2 or L2–L3	Japan
84	Friis Pedersen, C., Eiskjær, S., Østerheden Andersen, M., Yacat Carreon, L., Doering, P.	2024	A propensity-matched study of patients with symptomatic lumbar spinal stenosis opting for surgery versus not	Denmark
85	Valsamis, E.M., Prats-Uribe, A., Koblbauer, I., Cole, S., Sayers, A., Whitehouse, M.R., Coward, G., Collins, G.S., Pinedo-Villanueva, R., Prieto-Alhambra, D., Rees, J.L.	2024	Reverse total shoulder replacement versus anatomical total shoulder replacement for osteoarthritis: population based cohort study using data from the National Joint Registry and Hospital Episode Statistics for England	UK

Table H
Inclusion/exclusion criteria charting table

ID	Population	Concept	Context	Types of Evidence Source
1	Patients who had a primary diagnosis of bilateral CP, underwent SEMLS, and had pre- and postoperative gait analysis evaluations within a 2.5-year time span were included.	Use propensity score matching to estimate the causal treatment effects of rectus femoris transfer (RFT) as part of a single-event multilevel surgery (SEMLS)	Children	Observational study; Author's Institution
2	Children with bilateral CP who were between 6 and 18 years old at the time of baseline gait analyses and scheduled for SEMLS.	Construct Directed Acyclic Graph (DAG) to evaluate the impact of SEMLS on change in Gait Deviation Index (GDI)	Children	Observational study; Author's Institution
3	Elderly (66 to 93 years of age) Fee-for-Service (FFS) Medicare beneficiaries who underwent arthroplasty for a femoral neck fracture during 2017 to 2018 who (1) were enrolled in fee-for-service Medicare at least 12 months prior to their index surgery and (2) were alive and continuously enrolled during the 12-month follow-up period after the index arthroplasty.	Estimate the relationship between surgery type (THA versus HA) and outcomes (revision/ conversion and dislocation) at 12 months postoperatively, controlling for potential confounders using 2-stage instrumental variable technique	Adults	Observational study; Medicare beneficiaries (claims data)
4	Patients older than 40 who had undergone TKA or HTO as the primary surgical procedure without a history of having undergone either procedure during the preceding 2 years.	Inverse probability of treatment weighting (IPTW) weights individual patients on the basis of the inverse of the probability of their treatment allocation to create a pseudo-data set in which the distribution of potentially confounding variables is balanced between the treatment and control groups.	Adults	Observational study; South Korean National Health Insurance (NHI) claims database
5	Patients with a diagnosis of hip fracture who underwent physical therapy. The inclusion criteria were admission within the day after injury and surgery within 10 days of admission.	IPW was used to choose weights based on propensity scores in order to create a synthetic sample for which the distribution of measured covariates is independent of treatment assignment. Marginal structural model: (i) estimation of the propensity score; (ii) weighting; (iii) balance checking; and (iv) estimation of impact.	Adults	Observational study; Japan Rehabilitation Database
6	Conventional primary total hip replacement procedures performed for a primary diagnosis of osteoarthritis (OA) using an uncemented acetabular component and a modern bearing surface (highly cross-linked polyethylene or ceramic) were included.	As some surgeons use acetabular screws routinely and others only when concerned about cup stability, an instrumental variable (IV) analysis was used to adjust for this confounding (i.e. greater use of screws in more complex procedures). This instrumental variable identifies patients who would have received a THA with screws from some surgeons and no screws from other surgeons, based on surgeon preference rather than patient characteristics.	Adults	Observational study; Australian Orthopaedic Association National Joint Replacement Registry
7	Scheduled for primary THA due to OA, ≥ 18 years old, and mentally and physically capable of completing the Dutch questionnaires.	As all surgeons at LangeLand Hospital exclusively used PLA, and all surgeons at Alrijne hospital exclusively performed DAA, hospital acted as an instrumental variable. Mixed-effect models (including subject-specific intercepts) were fitted (linear) models (if continuous) with corresponding effect estimates, or logistic models (if binary) with corresponding odds ratios (OR) to estimate the effect of THA approach on postoperative “activity and participation.”	Adults	Observational study; Longitudinal Leiden Orthopaedic Outcome of Osteo-Arthritis Study
8	All elective primary TKAs for any diagnosis	Confounding was addressed using propensity score weights. Marginal Cox-regression models adjusting for surgeon clustering were used.	Adults	Observational study; Total Joint Replacement Registry (TJRR)
9	Patients who underwent primary elective anatomical total SAs (TSAs) for osteoarthritis (OA) and patients who underwent primary reverse total shoulder arthroplasties (RTSAs) for rotator cuff arthropathy (RCA).	Cox proportional hazard regression weighted with the inverse propensity score to reduce selection bias. A cluster term was also included in the propensity score-weighted regression model to adjust for surgeon differences. Analyses were stratified by procedure type because preliminary findings showed a heterogenous effect between TSA and RTSA	Adults	Observational study; healthcare registry
10	Patients who underwent long spinal fusion surgery with LLIF for ASD, age ≥ 18 years, ≥ four fused levels and at least one level using LLIF, and presence of at least one spinal	A propensity score (PS) for receiving two-staged surgery was estimated using a logistic regression model. Stabilized inverse probability treatment weighting (SIPTW) was used to balance	Adults	Observational study; Author's Institution

	deformity marker: scoliosis Cobb angle ≥ 20°, sagittal vertical axis ≥ 5 cm, pelvic tilt ≥ 25°, pelvic incidence minus lumbar lordosis angle ≥ 10°, and/or thoracic kyphosis ≥ 60°.	differences in baseline characteristics and surgical factors between the two groups. Stabilized propensity score weighted logistic regression models were used to investigate associations between two-stage and primary and secondary outcomes.		
11	Inclusion criteria were age between 50 and 79. Patients who underwent primary arthroplasty for displaced femoral neck fractures.	Instrumental variable analysis was performed with use of the methods outlined by Columbo et al. The instrument utilized in the present analysis was institutional preference, defined as the tendency of a hospital or department to treat displaced femoral neck fractures with either HA or THA.	Adults	Observational study; Australian Orthopaedic Association National Joint Replacement Registry
12	157 consecutive patients (64 men, 93 women) aged 65.7 ± 11.3 years (mean ± SD; range, 28–87 y) without a prior lumbar surgical history were treated using single-level PLIF without concomitant laminectomy at other levels	To adjust for potential confounders, we used the inverse probability of treatment weighting (IPTW) method of propensity scores. Propensity scores were estimated by a multiple logistic regression analysis that included all of the preoperative variables.	Adults	Observational study; Author's Institution
13	All patients with Dupuytren's Contracture who underwent percutaneous needle aponeurotomy or limited fasciectomy were identified.	Propensity score–based inverse probability weighting was used as the primary method to account for the between-group differences.To calculate the propensity score, we used multivariate logistic regression modeling with the pretreatment factors as independent vari-ables and treatment technique as the dependent variable. To minimize the risk of further bias,we also included possible confounders of the relation between treatment and outcomes, including sex, diabetes, smoking status, bilateral disease, and familial history of the disease.	Adults	Observational study; hand surgery practice sites (registry)
14	Patients with degenerative spondylolisthesis with slip-page at L3 or L4 of>3% who underwent spine surgery. We enrolled patients with slippage between 5% and 16.8%.	To adjust forpotential confounders, we used the inverse probability oftreatment weighting (IPTW) method of propensityscores. Propensity scores were estimated using a multiple logistic regression analysis that included sex, age at surgery, number of operation level, preoperative percent of slippage, follow-up period until final patient assessment,and preoperative JOA score.	Adults	Observational study; Author's Institution
15	Patients with severe stable SCFE (posterior sloping angle (PSA) > 50°), undergoing D, were included in the study. During the same period a cohort of patients undergoing S to address severe SCFE, according to surgeons' preference and experience, was also included into the study.	For each patient, we estimated propensity scores (PS) for receiving D or S, using a binary logistic model that included baseline variables. We included in the PS model only baseline variables hypothetically related to the outcome. The balance of the PS was checked observing the overlap in the range of propensity scores across the two treatments and comparing the quintiles. Propensity scores were then used to derive inverse probability of treatment weights (IPTW). Then, the IPTW was used to adjust the RR for early revision surgery in the two groups.	Children	Observational study; Author's Institution
16	Patients were included if they were over 18 years old and had undergone a lumbar interbody fusion using either an anterior or posterior approach.	Inverse probability weighted propensity scores (IPWPS) wereconstructed to control for known imbalances between groups.Weights were computed by estimating the probability of receivingan anterior over a posterior fusion with a multivariate logisticregression model. The inverse propensity scores were then usedto weight regression models that tested the association of surgicalapproach with outcomes for postoperative complications, healthresource utilization and reoperations	Adults	Observational study; MarketScan database
17	192 consecutive patients who underwent primary THA for osteonecrosis of the femoral head (ONFH) with a follow-up period of longer than 2 years (mean 10 years; range 2–26 years) were included in this study.	To identify whether the navigation system was useful to prevent dislocation, the inverse probability of treatment-weighted (IPTW) Cox regression analysis using a propensity score. The propensity score for receiving navigation was calculated using multivariable logistic regression, and included sex, age at surgery, BMI, and femoral head size as variables.<br>In addition, the Cox regression analysis including the propensity score as a covariate was also conducted to confirm robustness of the result from the IPTW Cox regression analysis.	Adults	Observational study; Author's Institution
18	Rupture of the anterior cruciate	To adjust for case mix, inverse probability of	Adults	Observational study;

	ligament (ACL) treated with dynamic intraligamentary stabilization (DIS) who were followed-up for at least 2 years after surgery, were eligible for inclusion in the study.	treatment weighting (IPTW) using the propensity score was applied to balance the two patient groups for patient and treatment characteristics. The propensity score was estimated without regard to outcome variables using multiple logistic regression. Meniscus surgery during index surgery and the patient characteristics of age, sex, and BMI at baseline, and Δ-Lachman, Tegner activity level, and Lysholm and IKDC score values at 6-month follow-up were included in the propensity score.		Author's Institution
19	Patients who underwent ACL reconstruction were consecutively included. Adults aged over 18 years with a first isolated ACL rupture who were surgically treated with the PT or HS graft technique were prospectively included by 1 of the 30 participating surgeons.	Graft type may have been motivated by the preferences of the surgeon or the center and by patients' characteristics, individuals in the 2 groups likely differed. To control for such differences, propensity score matching and inverse probability weighting treatment (IPWT) were performed to limit "indication bias."	Adults	Observational study; SoFCOT Symposium database on ACL reconstruction
20	All referrals to a single orthopedic surgery department for evaluation of radiologically confirmed spinal metastases were retrospectively included in this study.<br>Patients with symptomatic MSCC with neurologic impairment and the time of onset of symptoms were identified.	Propensity score analyses were performed with inverse-probability-of-treatment-weights (IPTW), and balance diagnostics were performed. The propensity score model was developed on multiply imputed data including all ten available variables of the dataset, whereas other analyses were done according to the complete-case principle.	Adults	Observational study; Author's Institution
21	Patients 18 to 60 years of age in whom a Type-III pilon or IIIB or C ankle fracture, a Type-III talar or calcaneal fracture, or an open or closed blast/crush foot injury had been treated with limb salvage or amputation and followed for 18 months.	For each outcome, the estimation procedure involved (1) simulating the outcomes that salvage-treated patients would have had if they had undergone amputation and (2) comparing the distribution of the observed outcomes of patients treated with salvage with the distribution of their simulated amputation outcomes. Conceptually, the simulation of the amputation outcome for a salvage-treated patient with covariates x involves 2 steps. First, the percentile (p) of the salvage-treated patient's outcome is calculated based on the distribution of salvage outcomes among salvage-treated patients who share covariates x. Second, the salvage-treated patient's potential outcome had an amputation been performed is simulated by calculating the p-th quantile of the distribution of the outcomes for amputees who share covariates x.	Adults	Observational study; post-hoc analysis of data from two previous studies (OUTLET and TAOS)
22	Inclusion criteria were LSS with a posteriordecompression and pre- and postoperative COMI assessment between 3 and 24 months	The inverse probability of treatment weighting (IPTW)<br>using the propensity score was applied to balance the<br>treatment groups for their baseline characteristics. IPTW<br>using the propensity score uses weights based on the pro-<br>pensity score to create a synthetic sample in which the<br>distribution of measured baseline covariates is independent<br>of treatment assignment. The propensity score was esti-<br>mated without regard to outcome variables, using multiple<br>logistic regression analysis.	Adults	Observational study; international spine registry (SpineTango)
23	Our study cohort comprised patients with knee OA, and who had received treatment of knee OA for 4 or more times. Patients who receivedTKA were included.	Propensity score: Propensity score stratification was applied to replace the wide host of observable confounding factors that may be present in an observational study with a variable of these factors. To derive the propensity score in this study, patient characteristics were entered into a logistic regression model predicting selection for TKA therapy.<br>Instrumental variable analysis: Instrumental variable analysis was used to account for both measured and unmeasured confounding factors. The instrumental variable was estimated by first calculating the proportion of knee OA patients	Adults	Observational study; Taiwan's National Health Insurance Research Database

		who received TKA in each hospital.		
24	At least 45 years old with meniscal tear and osteoarthriticchanges documented on MRI	To reduce potential bias, we used the propensity score approach to balance the APM and PT groups with respect to prognostically important variables. The variables included in the propensity score model (modeling propensity to be in the APM group) included baseline demographic variables (age, sex, race, body mass index), pain severity, and KL grade. We then compared changes from baseline to 60 months in KL grade (changes had a normal appearing distribution) and in the OARSI score between the two treatment groups using two-sample t-tests, both unadjusted and with the inverse probability weights.	Adults	Observational study; post-hoc analysis of data from an RCT
25	Femoral neck fracture with use of either a modular unipolar or bipolar prosthesis. Data from September 1, 1999, to December31, 2018, were included.	We conducted an instrumental variable analysis based onhospital preference for the use of bipolar or unipolar pros-theses. Our instrument was the proportion of bipolar proce-dures among the total number of hemiarthroplasty procedures(modular unipolar and bipolar) performed at each hospital inthe year prior to the index operation, according to a previouslydescribed method designed for time-to-event data.	Adults	Observational study; Australian Orthopaedic Association National Joint Replacement Registry
26	The study population included patients who had undergone UKA or HTO as documented by primary diagnosis and first additional diagnosis	Propensity score (PS)-based analyses were used to simultaneously control for a large number of covariates. Theses analyses provide more robust, less biased estimates when the number of outcome events is low relative to the number of confounders. We fit the data to a logistic regression model to estimate the probability of treatment with UKA versus HTO and performed one-to-one nearest neighbor matching based on the estimated PS after adjusting for all covariates including age category, sex, comorbidities, and co-medications. Inverse probability of treatment weighting (IPTW) was calculated as 1/PS for those who were treated with UKA and 1/(1−PS) for those who were treated with HTO.	Adults	Observational study; Korean National Health Insurance claims
27	Primary, unilateral, or staged bilateral, Posterior Approach Total Hip Arthroplasty (PA THA)	To adjust for treatment/group selection bias and to balance the cohorts, a logistic regression was used to calculate propensity scores for each patient based on age, gender, body mass index, femoral cementation, history of spine fusion, and Charlson Co-morbidity Index. The inverse probability of treatment weight (IPTW) was then calculated as the inverse of the propensity score.	Adults	Observational study; Author's Institution
28	The inclusion criteria were age > 18 years, an acute spontaneous Achilles tendon rupture as a single leg injury, a ruptured area at only the midsubstance with a gap measuring less than 2 cm, follow-up for at least 1 year after primary operation, and provision of written informed consent by the patient.	Inverse-probability-treatment-weighted (IPTW) propensity score analyses of SSI rate and ATRS were performed. Sampling weights for each patient are created by a propensity score, the probability of being assigned one of the interventions, usually estimated by binary logistic regression (i.e., a treatment model) with the study's intervention assignments as the dependent variable and the covariates to be balanced as the independent variables. For SSI rate and ATRS, treatment models were fitted using binary logistic regression and including prognostic factors from subject matter knowledge in the literature. For SSI rate, factors included age, sex, DM, obesity defined by the World Health Organization definition for Asians (body mass index ≥ 25), current tobacco use, and current steroid use. For ATRS, factors included age, sex, current steroid use, DM, time from the day of the injury to the day of the operation, and tendon gap length.	Adults	Observational study; Author's Institution
29	The study cohort comprised people aged 50 years and older admitted to an Australian hospital with an intertrochanteric femoral fracture and treated with surgical fixation (short IM nail, long IM nail or SHS)	The primary analysis used adjusted multilevel logistic regression to test the association (expressed as an Odds Ratio and 95% confidence interval) between types of surgical fixation and 30-day mortality, adjusted for covariates and hospital-level clustering. Given the known institutional variation, an instrumental variable analysis was performed using hospital preference for type of surgical fixation as the instrument and	Adults	Observational study; registry data Australian Hip Fracture Registry (AHFR) and the Australian Institute of Health and Welfare (AIHW) National Death Index (NDI)

		individual 30-day mortality as the outcome. Instrumental variable analysis does not rely on the assumption of no unmeasured confounding and allows casual inference from observational data. To further explore the association within the 30-day period, Cox proportional hazard modelling was used (expressed as a Hazard Ratio and 95% confidence interval), adjusted for covariates.		
30	Stage 1 – people undergoing unicompartmental knee replacement surgery or total knee replacement surgery who met the TOPKAT eligibility criteria. Stage 2 – participants with an American Society of Anesthesiologists grade of ≥ 3	In stage 1, four propensity score-based approaches and inverse probability weighting were used to account for measured confounding: (1) propensity score matching (1 : 5), (2) stratification based on the distribution of the propensity score in the whole cohort, (3) stratification based on the unicompartmental knee replacement cohort and (4) propensity score adjustment (linear and non-linear models). For each outcome, a logistic regression model was used to calculate the propensity score for unicompartmental knee replacement using patient-level characteristics, including demographics, preoperative patient-reported outcome measures, comorbidities and procedures recorded within the 3 years before surgery. Missing body mass index data and preoperative patient-reported outcome measures were imputed using multiple imputation by chained equations. Covariate balance was assessed using absolute standardised mean difference, with a predefined cut-off point of 0.1. We also explored four potential instrumental variables: surgeon preference, hospital preference, geographical location and calendar time. When certain assumptions are fulfilled, instrumental variable analyses can account for measured and unmeasured confounders. Key instrumental variable assumptions were checked with F - statistics, odds ratios (strength of the instrument) and absolute standardised mean differences (lack of an association between the instrument and the confounders)	Adults	Observational study; Registry
31	We recruited a cohort of patients with symptomatic rotator cuff tears in a multi-center longitudinal study termed as Rotator Cuff Outcomes Workgroup (ROW). Patients aged 45 and older.	A propensity score was estimated for each patient in each imputation data set as the probability of surgery using a multivariable logistic regression. The final propensity score for each patient was the average of 20 propensity scores calculated from imputed complete datasets. Matching weights were calculated for both surgery and non-operative groups. The standardized mean difference (SMD) was calculated to estimate the improved balance among covariates achieved by propensity score weight matching. In addition to the fixed effects mentioned above, individual participant ID was included as the random effect and inverse probability weighting (IPW) was used to adjust estimates for the propensity of treatment group membership.	Adults	Observational study; Sports/shoulder clinics in 3 academic and 1 community settings
32	Elective primary total knee arthroplasty (TKAs), osteoarthritis as the primary diagnosis, patients aged 18 years or older at the time of their procedure, CoCr alloy on HXLPE (CoCr-HXLPE), or CoCr-conventional polyethylene knee bearings.	Because tibial insert material was not randomly assigned, we addressed possible confounding using a weighted propensity score approach. (1) the propensity score was estimated in the conventional way by fitting a logistic regression model and estimating the conditional probability of treatment assignment for each record; (2) we checked that cases in one bearing group had comparable counterparts with respect to their covariate distribution in the other bearing group; those that did not were excluded based on a caliper width of 0.2 SD of the logit propensity score; (3) we stratified the sample into six strata based on the estimated logit propensity score; and (4) we calculated the weight for each record based on the number of units in a stratum multiplied by the proportion of units assigned to the treatment group of interest in the data and divided by the number of records assigned to the treatment group of	Adults	Observational study; Kaiser Permanente Total Joint Replacement Registry (TJRR)

		interest in that particular stratum.		
33	All elective nonbilateral primary THAs, in which patients were at least 18 years old at the time of their procedure and had metal-on-conventional polyethylene or metal-on-HXLPE bearing surfaces.	Because bearing surface material was not randomly assigned, we addressed observed confounding using a propensity score approach. (1) the propensity score was estimated in the conventional way by fitting a logistic regression model and estimating the conditional probability of treatment assignment for each record; (2) we checked that cases in one bearing group had comparable counterparts with respect to their covariate distribution in the other bearing group and those that did not were excluded based on a caliper width of 0.2 SD of the logit propensity score; (3) we stratified the sample into six strata based on the estimated logit propensity score; and finally (4) we calculated the weight for each record based on the number of units in a stratum multiplied by the proportion of units assigned to the treatment group of interest in the data and divided by the number of records assigned to the treatment group of interest in that particular stratum.	Adults	Observational study; Kaiser Permanente Total Joint Replacement Registry (TJRR)
34	Patients were included in this study if they were (1) at least 18 years of age at the time of surgery, (2) had a full-thickness tear of the supraspinatus tendon (with or without posterior extension into the infraspinatus tendon) and (3) underwent arthroscopic rotator cuff repair using either a knotted suture-bridging or knotless tape-bridging TOE RCR technique.	Since each technique was used during chronologically distinct periods and in different sized cohorts, a propensity score model was initially built for the purpose of balancing the two treatment groups on 11 covariates that may differ between technique groups. Then, inverse propensity score weighting (IPSW) was used to estimate the causal relationship between the repair technique and outcome score within the multiple regression models.	Adults	Observational study; Author's Institution
35	All UKAs indicated by osteoarthritis in knees without prior surgery.	This study is subjected to confounding due to the non-random<br>assignment of prior UKA or HTO. Therefore, PS-IPTW was<br>utilized to account for confounding by indication. PS were<br>estimated with logistic regression and applied by IPTW with<br>stabilized weights aiming to estimate the average effect of<br>treatment.	Adults	Observational study; Danish Knee Arthroplasty Registry
36	Patients older than 17 years of age, with an acute bimalleolar equivalent, bimalleolar, or trimalleolar ankle fracture, which we considered complex fractures, were treated by AORIF.<br><br>Any patient from the department's ankle fracture database who was treated by ORIF and met the same inclusion and exclusion criteria applied for the AORIF cohort was eligible for the matching process<br><br>Inclusion:<br>- Unimalleolar fracture + syndesmotic disruption = bimalleolar equivalent<br>- Bi- or trimalleolar fracture ± ligamentous injury<br>- >17 years<br>- Date of injury ≤14 days to surgery<br>- Written informed consent	Propensity score matching was conducted between the previously outlined AORIF cohort and a retrospective cohort treated by ORIF only (ORIF cohort). The 7 covariates included in the model were age, sex, American Society of Anesthesiologists (ASA) class, body mass index (BMI), side, number of malleoli fractured, and follow-up. Based on these parameters, the authors aimed to generate pairs of treated (AORIF) and untreated (ORIF) subjects using logistic regression-based propensity scores applying nearest-neighbor matching. A predefined caliper width of 0.1 without case replacement was used.	Adults	Observational study; Author's Institution
37	Patients with a minimum age of 18 years who underwent isolated mowHTO or isolated lcwDFO for symptomatic varus malalignment with a minimum of 24 months of postoperative follow-up.	To compare the isolated effect of the type of osteotomy relatively independent from confounders, baseline variables were equalized between the 2 groups via propensity score matching, a tool for causal inference in nonrandomized studies allowing for adjustment of covariates. For the matching, the following clinically relevant potentially confounding	Adults	Observational study; Author's Institution

		covariates were chosen as previously proposed in the setting of alignment-correcting osteotomies: age at surgery, sex, body mass index (BMI), preoperative mechanical femorotibial axis, and time interval since surgery. Popensity scores were calculated via multiple logistic regression analysis to match the groups for the following covariates: age at surgery, sex, BMI, preoperative mechanical femorotibial axis, and follow-up. A caliper width of 0.2 was set, in accordance with previous studies. Nearest neighbor matching according to the propensity scores (greedy algorithm) was performed with a 1-to-1 match ratio.		
38	OA patients aged 45 less than or equal to enrollment age less than or equal to 80 years old unilateral TKA required able to cooperate in the follow-up with good compliance volunteered to participate in this study and signed written informed consent.<br>Patients with Kellgren–Lawrence grade IV knee osteoarthritis	To reduce the effects of selection bias and potential confounding factors, we used age, sex, left and right, BMI, and preoperative HKA for 1 : 2 propensity score matching	Adults	Observational study; Author's Institution
39	Aged between 45 and 79 years, of all ethnic groups, who had, and those who were at high risk for developing, symptomatic knee osteoarthritis.	The difference in risk for knee replacement surgery between participants who did and those who did not undergo arthroscopic meniscectomy during follow up could be biased by confounding baseline characteristics (confounding by indication). To adjust for these confounding baseline characteristics we matched participants based on their propensity scores. Propensity scores were estimated independently for each imputed dataset, using a logistic regression model with arthroscopic meniscectomy during follow up as the dependent variable in relation to the baseline characteristics. We used a 1:1 matching algorithm without replacement to match exposed and nonexposed individuals on propensity score within a caliper of 0.2 standard deviation of the logit of the propensity score	Adults	Observational study; Osteoarthritis Initiative (OAI) database
40	The study population included individuals who had undergone unicompartmental knee arthroplasty (UKA) or total knee arthroplasty (TKA) for knee osteoarthritis, as documented by primary diagnosis and first additional diagnosis.	The results of the study should be randomly selected to ensure that there is no difference in characteristics. However, case–control study works on a specific group, so there is no random assignment, and selection bias cannot be avoided. To minimize this problem, the propensity score (PS) matching is used. PS-based analyses were used to control for a large number of covariates simultaneously. These analyses provide more robust, less biased estimates when the number of outcome events is low relative to the number of confounders. We fitted the data to a logistic regression model to estimate the probability of treatment with TKA versus UKA and performed one-to-one nearest neighbor matching based on the estimated PS, and adjusted for all covariates including age category, gender, comorbidities, and co-medication. Inverse probability of treatment weighting (IPTW) was calculated as 1/PS for those who were treated with TKA and 1/(1-PS) for those who were treated with UKA. This method weights individual patients based on the inverse of the probability of their treatment allocation, conditional on baseline characteristics, to create a pseudo-dataset in which the distribution of potentially confounding variables is balanced between the treatment and control groups.	Adults	Observational study; South Korean National Health Insurance (NHI) Claims Database
41	Inclusion criteria were cervical disc herniation with radiculopathy or foraminal stenosis with radiculopathy, which had been surgically treated with anterior decompression followed either by insertion of an ADR or by fusion with either a plate with	To select confounders that might give rise to treatment selection bias, we used present knowledge and a directed acyclic graph. A propensity score was constructed to control for the potential covariates. Patients were then matched one to one based on estimated propensity scores limited by the requirement that the difference between scores should be no larger than 0.001,	Adults	Observational study; National Swedish Spine Register

	autograft/allograft, a stand-alone cage, or a cage with plate.	surgery should have taken place within 180 days, and follow-up NDI should be available. Matching was performed using logistic regression models with ADR as the outcome and the confounders as explanatory variables.		
42	Patients who underwent lumbar discectomy (DSC) or arthroscopic anterior cruciate ligament reconstruction (ACLR) were retrospectively identified. The DSC cohort included patients who underwent elective DSC.	We used propensity score matching to generate matched groups of DSC and ACLR patients that had similar distributions of patient's age, sex, race, and number of comorbidities preoperatively. For this, we generated propensity scores using Stata's –pscore- command, we checked for the balance of the scores across the DSC and ACLR cohorts within blocks of the propensity score, and we used the nearest neighbor 1:1 matching without replacement. To check for the robustness of the findings, we used another propensity score method—Inverse Probability of Treatment Weighting (IPTW)—to generate weights that are the inverse probability of being in a particular surgical cohort	Adults and mature adolescen ts (>13y)	Observational study; administrative and medical record data from a tertiary care academic medical center
43	Patients undergoing single-level anterior cervical discectomy and fusion (ACDF) for cervical radiculopathy or cervical spondylotic myelopathy	To test the utility of IONM in preventing postoperative neurological deficits in ACDF surgery, multivariable logistic regression analysis of the entire study population was performed with neurological deficits as the outcome and IONM, age, sex, race, surgical indication, multilevel fusion, CDCI, and EHI as the covariates. Multivariable logistic models were also generated for the secondary outcomes of length of stay (continuous variable), any in-hospital complication, nerve root or cord injury, and any in-hospital complication except nerve root injury and cord injury. As a confirmatory analysis, 1-to-1 greedy nearest-neighbor propensity score matching (PSM) was performed using IONM as the treatment indicator, and age, sex, race, surgical indication, multilevel fusion, myelopathy, radiculopathy, CDCI, EHI, and hospital geography (urban/rural) as covariates. Average treatment effect estimation with inverse probability weighting was performed for neurological deficits in the unmatched population with IONM as the treatment.	Adults	Observational study; Statewide Planning and Research Cooperative System (SPARCS)
44	This study was conducted on 52 consecutive patients over 8-years of-age with CMT who had undergone surgical release of the SCM. We followed up all the patients for more than 12 months after surgery including the medical records, clinical photographs, and radiographs.	To reduce potential selection biases arising from nonrandom allocation of observational studies, the inverse probability of treatment weighting via propensity score was used.The propensity score was generated from logistic regression model based on covariates including age, sex, location, recurrence, preoperative rotation deficit, and preoperative flexion deficit. To examine the association between the use of ADM and the surgical outcomes, we performed multivariate logistic regression and linear regression, after adjusting for age, sex, and several confounders, and also weighted model via propensity score was used.	Patients >8 years of age	Observational study; Author's Institution
45	Patients undergoing revision interbody fusion surgery. We included patients who underwent primary posterior decompression surgery for neurological symptoms and/or neuropathic leg pain. Patients who had recurrent or new-onset neurological symptoms and/or leg pain underwent revision interbody fusion surgery.	In this study, we calculated the PS for the decision to choose between the LLIF and PLIF/TLIF surgical techniques and utilized the score for adjusting potential confounders. Comparisons between categorical variables were performed utilizing Fisher's exact test. Comparisons between continuous variables were performed using the paired t-test or Mann-Whitney U-test. The PS of each patient, defined as the probability of being treated with LLIF or PLIF/TLIF, was calculated using a logistic regression model including all variables. The C-statistic value of the PS regression model was calculated to assess the appropriateness of variable selection. After calculating the PS, logistic regression and linear regression analyses were conducted by setting each outcome measure as a response variable, LLIF or PLIF/TLIF as the predictor variable, and	Adults	Observational study; Author's Institution

		the PS as the single confounding variable. Since the expected numbers of observed events in the logistic regression analyses were small and complete or near-complete separation would occur, we utilized Firth's bias reduction method for maximum likelihood estimation.		
46	Patients with lumbar synovial cysts undergoing laminectomy ± instrumented lumbar fusion	Patients in the LF group were propensity score–matched in a 2:1 ratio (laminectomy:LF) to the laminectomy-only group.<br>The nearest neighbor technique and calipers of the width equal to 0.2 of the pooled standard deviation of the logit of the propensity score were used.<br>The 2-year cumulative incidence of SS for each surgical treatment for the matched cohort was generated using Kaplan-Meier survival analysis and presented as cumulative incidence.	Adults	Observational study; IBM MarketScan Commercial Claims and Encounters Database
47	All adults with the diagnosis of dementia who underwent emergency hip fracture surgery for dFNF, i.e. Garden III and IV, in Sweden were considered for inclusion in the study.	The cohorts were also matched at a 1:1 ratio using propensity score matching. Nearest neighbor matching with a caliper of 0.2 was selected as the matching algorithm. A conditional Poisson regression model and a Cox regression model with shared frailty were used for the matched cohorts when analyzing 30-day and 1-year postoperative mortality, respectively.	Adults	Observational study; the Rikshoft register, the Swedish National Quality Registry for Hip Fracture Patients and Treatment
48	Patients undergoing single-stage lumbar fusions.	K-nearest neighbor propensity score matching was performed for anterior- versus posterior- approach lumbar fusions using the Stata psmatch2 procedure with a logit model, with k = 2. For match balance diagnostics, a threshold of Rubin's B≤ 0.25 and 0.5 ≤Rubin's R≤ 2.0 were used. Covariates were compared before and after matching, and percent bias and bias reduction were calculated, as well as mean and median bias before and after matching. The average treatment effect on the treated of anterior approach fusion on 30-day VTE rate was reported, and is hereafter referred to as the treatment effect. Bootstrapped p-values and 95 % confidence intervals were calculated from Abadie-Imbens heteroskedasticity-consistent analytical standard errors. Propensity score adjusted logistic regression was performed to confirm any association between anterior approach lumbar fusion and VTE, and to determine the strength of association via the calculated odds ratios.	Adults	Observational study; Author's Institution
49	Included were implantations with cemented femoral, tibial, and patellar components (with components designed to be used with cement).	propensity-score-weighted Cox proportional-hazards regression with cluster robust standard errors at the surgeon level was used. Propensity score weights (inverse probability of treatment) were calculated separately with use of a multivariable logistic regression that included all covariates as predictors of treatment assignment. The standardized difference for each covariate before and after applying propensity-score adjustment indicates the balance in covariate distribution between groups; a standardized difference <0.1 indicates that balance is achieved.	Adults	Observational study; Kaiser Permanente Total Joint Replacement Registry (KP TJRR)
50	Patients undergoing primary cemented THA or TKA.	In order to account for potential baseline differences between patients who did and those who did not receive dilute PI irrigation, a propensity score was generated (separate scores were generated for the TKA and THA cases). The propensity score was created using a logistic regression model in which being treated with dilute PI irrigation was the outcome. The score was then used to create inverse probability of treatment weights (IPTWs), which were incorporated into the models evaluating the association of PI with the study outcome.	Adults	Observational study; Registry
51	Patients who were admitted with C2 fracture and further identified odontoid fracture using Japanese disease codes. The inclusion criteria were age of ≥ 65 years and admission for treatment of odontoid	We used a propensity score-based method to account for differences in observed factors that might affect either the treatment assignment or outcome. Covariate selection was prespecified by using both potential confounding factors and variables that can serve as proxies for unknown or	Adults	Observational study; Japanese Diagnosis Procedure Combination database

	fracture by at least one of three procedures (halo-vest immobilization (K1444), ASF(K142-1), or PSF (K142-2)) during hospitalization.	unmeasured confounding variables. The propensity score was estimated using a multinomial logistic model with the procedure received as the dependent variable and the baseline factors as independent variables. To balance the patients' baseline characteristics among the three procedures, a matching weight approach was applied. Matching weights is recommended for comparing outcomes across multiple treatment groups when the covariates' overlaps are relatively limited, outcomes are rare, or exposure distributions are unequal. Each patient was weighted by the inverse probability with the lower propensity score of the three procedures as the numerator. The patients would receive each of the treatments among halo-vest immobilization, ASF, or PSF, allowing average treatment effects to be estimated.		
52	Patients with a diagnosis of lumbar disc herniation were identified. The inclusion criterion was patients who underwent microendoscopic discectomy, microdiscectomy, or open discectomy at a single level for lumbar disc herniation.	To determine which procedure had the higher revision rate, we used a propensity score overlap weighting analysis to adjust for measured confounders such as age, sex, comorbidities, and hospital type between the microendoscopic discectomy and microdiscectomy or open discectomy groups. After propensity score overlap weighting, we used Cox proportional hazards models to estimate HRs for outcomes. The 95% CIs in weighted analyses were calculated using robust variance estimators. Because propensity score overlap weighting accounts for confounding, we performed no further adjustment in the Cox models. Interactions between exposure variables and time were verified by the proportionality assumption. We created a propensity score for receiving microendoscopic discectomy using a logistic regression model.	Adults	Observational study; JMDC claims database
53	Patients aged 18 years or older with computerized tomography (CT) scan confirmed multiple rib fractures (defined as three or more ipsilateral rib fractures) after blunt thoracic trauma were eligible for inclusion.	To control for potential confounding PS matching was performed. The PS was estimated using binary logistic regression analysis. A 1:1 nearest neighbor matching was performed, with a maximum caliper of 0.15 of the SD of the natural logarithm of the PS using the MatchIt algorithm in R. After PS matching, the distributions of the baseline characteristics were compared between the study groups and quantified using SMD. Primary analyses was conducted using a data set of PS-matched subjects. For the primary and secondary outcomes, the relationship between rib fracture fixation and outcomes was assessed using linear regression analysis for continuous outcomes and binary logistic regression analysis for binary outcomes.	Adults	Observational study; Author's Institution
54	patients with a diagnosis of symptomatic DS (slip more than 3 mm, with severe lumbar spinal stenosis (grade C or D on Schizas classification), that were submitted to single-level minimally invasive lumbar interbody fusion with MIDLIF or MIS-TLIF.	The matching was based on 1:1 nearest neighbor matching without replacement, with a caliper. Propensity score was used as distance and estimated with logistic regression, targeting the average treatment effect. The following covariates were used for matching: age, sex, smoker status, BMI (split at 30), previous surgery at the same level, stenosis grade, severe disproportion according to GAP score. Covariates assessment was based on standardized mean difference (SMD). SMD < 0.1 was considered an adequate balance for a variable between the groups, while values of SMD > 0.2 were considered as serious imbalance. Before matching, multiple missing imputation was performed with additive regression, bootstrapping and predictive mean matching.	Adults	Observational study; Author's Institution
55	Patients who were at least 18 years of age and had an MRI diagnosis of a full-thickness rotator cuff tear.	To establish the best possible matching of patients in the surgical repair and nonoperative treatment groups, nearest-neighbor without replacement (caliper = 0.3) propensity score analysis (SPSS, version 25.0; IBM) was employed with patients matched by factors thought to influence the	Adults	Observational study; Michigan Shoulder Registry

		outcome of rotator cuff tears.		
56	Patients who underwent surgical management for grade I lumbar spondylolisthesis in the form of decompression or decompression with fusion. Inclusion criteria included neurogenic claudication without disc herniation and self-reported axial lumbar/lumbosacral back pain. The American Society of Anesthesiologists (ASA) Score grades patients on a scale of I to VI with a higher number indicating increasing severity of systemic disease	As patients were not randomized to treatment arms, two alternative matching methods were employed to minimize selection bias when estimating causal treatment effects: (1) propensity score matching, PSM; and (2) coarsened exact matching, CEM. In PSM, multivariable logistic regression first delineates factors associated with treatment choice, followed by logistic regression to calculate individual propensity scores per covariate. "Case" (fusion) and "control" (decompression alone) patients are then paired 1:1 on these propensity scores via exact matching. Standardized differences are estimated before and after matching to evaluate the balance of covariates; small absolute values (<0.1) indicate balance between treatment groups [24]. CEM, meanwhile, is a nonparametric quasi-experimental matching approach that uses monotonic imbalance bounding to reduce the imbalance in covariates between two groups. CEM uses covariates presumed by the investigator to explain variance in the outcome of interest to create broader categorical bins, and patients are matched within bins; this technique allows for a greater number of successfully matched patients. In some cases, CEM has yielded estimates of causal effects that are lower in variance and bias than PSM.	Adults	Observational study; Author's Institution
57	All patients undergoing neurosurgical procedures.	PSM for the preoperative characteristic of the multilevel ACDF and LAMP groups was performed before further analysis. The paired t-test and McNemar test were used to evaluate significant differences between these groups. Multivariable logistic regression analysis provided odds ratios (OR) and 95% confidence intervals (CI) for complications in these two groups, which were adjusted for	Adults	Observational study; American College of Surgeons National Surgical Quality Improvement Program Participant Use Data Files (ACS NSQIP PUF)
58	Patients of ≥65 years of age who underwent open reduction and internal fixation of the distal radius with a VLP.	To adjust for baseline differences between the two groups, a propensity score algorithm was used to match the two groups in a 4:1 ratio. We repeated the primary analysis of the entire cohort using stabilised inverse probability of treatment weighting (IPTW).	Adults	Observational study; TRON
59	All patients who underwent a primary THA for osteoarthritis via anterior approach or posterior approach were included in the study.	Baseline patient, hospital, and procedural characteristics between THA cohorts were compared using Student's t-tests for continuous variables and Chi-square tests for categorical variables. Potential causal relationships between variables were described in a Directed Acyclic Graph (DAG). Effects of surgical approaches were estimated using both a conventional outcome regression method and a doubly robust estimation method. In the conventional outcome regression model, a multivariable logistic regression of THA surgical approach on PJI was conditionally adjusted for. In the doubly robust estimation method, propensity score-matching (PSM) was first used to balance potential confounding factors between anterior and posterior approaches. Logistic regression was used to estimate the propensity score. Two PSM methods were used, namely 1:1 nearest neighbor-matching without replacement and subclassification (ie, observations were grouped into 500 subclasses based on their propensity score). Covariance balance after matching was assessed using standardized mean difference (SMD). Subsequently, a multivariable logistic regression model incorporating the inverse probability weights from the PSM was constructed to assess the effects of THA approaches on the 90-day PJI rate. The weighted logistic regression model was adjusted for the same covariates as the ones in the conventional outcome regression method.	Adults	Observational study; New South Wales (NSW) Admitted Patient Data Collection (APDC) and the AOANJRR
60	Patients undergoing primary total	Propensity score matching (PSM) was used to	Adults	Observational study;

	knee arthroplasty (TKA) for end-stage osteoarthritis. Inclusion criteria included patients having completed both pre- and postoperative Knee Injury and Osteoarthritis Outcome Score (KOOS) and KOOS for Joint Replacement (KOOS, JR) scores after 12 months from surgery.	establish covariate balance between the two treatment pathways. The method for matching was a 2:1 nearest neighbor logistic regression matching algorithm. Group differences in continuous variables were assessed using paired t-tests. Group differences in categorical variables were assessed using Chi-Square tests. To estimate the treatment effect of the digital physiotherapy application and its standard error, we fit linear regression models to each LOS variable.		Author's Institution
61	The cohort comprised all consecutive patients hospitalized with a primary diagnosis of femoral neck fracture who underwent surgery, either HA or THA.	Propensity score matching was used to identify a cohort of patients with similar baseline characteristics to account for differences in baseline characteristics between the two groups of eligible participants. A propensity score is the conditional probability of having a specific exposure such as HA versus THA on a set of baseline measured covariates. We estimated the propensity score using a multivariable logistic-regression model, with THA as the dependent variable and all the baseline characteristics. Then we matched patients in the HA and THA groups with a 1:1 matching protocol without replacement. We set caliper width for matching equal to 0.2 of the standard deviation of the logit of the propensity score. We estimated standardized differences for all the baseline covariates before and after matching to assess rematch baseline. Standardized differences of less than 10% for a given baseline covariates indicate relatively balanced matching. For the comparative risk of other postoperative complications—anesthesia time, length of hospital stay, and daily average medical cost—we used a Generalized Estimating Equations(GEE)s and calculated percent absolute risk differences (% absolute risk difference (RD), with 95% confidence intervals (CI)). In the matched cohort, we plotted survival curves for revision surgery using the Kaplan–Meier method and compared patients undergoing HA vs. THA using the log-rank test. A Cox regression model with robust variance estimator was used to estimate the time to revision surgery during hospitalization.	Adults	Observational study; Japanese Diagnosis Procedure Combination inpatient database
62	Individuals with traumatic axis fractures.	In order to balance comorbid conditions in the treatment groups, we used propensity scores to control for differences in measured covariates between surgical and nonsurgical cohorts. We estimated the probability of receiving surgery (PS). We then weighted the nonsurgical cohort by SMR weights, defined as the odds of the estimated probability of receiving surgery (PS/[1 − PS]). The surgical cohort received weights of 1. This process leads to a pseudopopulation of nonsurgical patients, whose covariate distribution mimics the one observed in surgery patients. This not only removes confounding by measured covariates but also allows us to estimate the association between surgery and all-cause mortality in patients who received surgery (treatment effect in treated).	Adults	Observational study; Medicare
63	Patients undergoing surgery on the cervical spine. Inclusion criteria were cervical disc herniation or foraminal stenosis with radiculopathy treated surgically with posterior foraminotomy or ACDF. ACDFs utilized plate with autograft/allograft, stand-alone cage, or cage with plate.	To select confounders that might give rise to treatment selection bias, we used present knowledge and a directed acyclic graph (DAG). A propensity score was constructed to control for the covariates. Patients were matched 1-to-1 based on estimated propensity scores limited by the requirement that the difference between scores should be no larger than 0.005 and surgery should have taken place within 180 days. Matching was performed using logistic regression models with posterior foraminotomy as the outcome and the confounders as explanatory variables. The mean and SD for numerical variables and the numbers and percentages for categorical variables, before and after matching, were calculated, together with	Adults	Observational study; Swespine

		a p value for the null hypothesis of equal distributions. Time plots illustrate primary and secondary outcomes after 1, 2, and 5 years of follow-up. Using ANCOVA, we compared the mean 5-year outcome values between the treatment groups, adjusted for the covariates included in the propensity score and also for the baseline values for each outcome. The mean differences of the 5-year values between the groups are presented with 95% CIs and p values. A positive mean difference corresponds to higher values for posterior foraminotomy (compared with ACDF). In addition, all available cases were analyzed, including all patients who had completed each follow-up, with imputation for missing data.		
64	Individuals who underwent either a UKR or TKR were identified. Study participants were required to have data captured over at least the year before surgery.	Propensity score matching was used to minimise confounding by observed characteristics. Propensity scores were generated using a large-scale regularised logistic regression fitted with a Laplace prior (LASSO) and the optimal hyperparameter determined through ten times cross validation to balance baseline covariates while avoiding overfitting. In the primary analyses, patients were matched on the propensity score using variable-ratio matching with a maximum ratio of UKR to TKR of 1:10. The balance of propensity score-matched cohorts was evaluated using standardised mean difference, with values of less than 0·1 taken to indicate negligible group differences. Propensity score distribution plots, normalised to the preference scale, were used to evaluate empirical equipoise. Cox proportional hazards models, with procedure type (UKR or TKR) as the sole explanatory variable and conditioned on the matched sets, were fitted to estimate the average treatment effect among patients undergoing UKR on the outcomes listed above. Participants were censored at the event of interest, end of study time-at-risk (ie, at 5 years for revision), or end of their observation period within the database.	Adults	Observational study; CCAE, MDCR, Optum, PharMetrics, THIN
65	All adult patients with a diagnosis of HPVO. We identified all discharge records that included osteomyelitis of the vertebral disk, pyogenic infection of intervertebral disk, unspecified discitis other infective spondylopathy, and epidural abscess.	We developed a propensity score to adjust for nonrandomized surgery allocation. The purpose of this score was to represent the predictors of surgical treatment as accurately as possible. Each patient who underwent surgery was weighted by the inverse of the probability that he/she would be selected for surgery. Each patient who underwent medical treatment was weighted by the inverse of the probability that he/she would be selected for medical treatment. To identify whether surgery was associated with pain or neurological sequelae at 4 months we used a multivariate logistic regression analysis adjusted for baseline pain and neurological deficit level. Associations were expressed as adjusted odds ratios (aOR) and 95% confidence intervals (95% CI).	Adults	Observational study; Author's Institution
66	Adult patients (aged >18 years) who underwent elective TAA or AA during the study period. Patients diagnosed with primary osteoarthritis.	To mitigate selection bias and confounding inherent in observational studies, propensity score matching (PSM) was implemented to create statistically comparable cohorts by matching patients undergoing TAA and AA on key demographic, hospital-related, and clinical characteristics, thereby simulating the conditions of a randomized controlled trial (RCT) and enhancing the validity of causal inferences. The propensity score was estimated through a logistic regression model incorporating 34 covariates spanning three major domains.	Adults	Observational study; National Inpatient Sample (NIS)
67	All adult patients (≥18 years) who underwent primary TKR for osteoarthritis only were eligible. All computer navigation and patient-specific instrumentation systems	Propensity scores were estimated using a logistic-regression-model approach with Stürmer weight trimming to improve the accuracy and precision of estimates. Propensity score-based weights were generated for the patient groups. Standardized	Adults	Observational study; NJR, NHS, ONS

	were eligible for inclusion.	mean differences were examined prior to and following the construction of weights to assess for covariate imbalance between the groups. These are computed by dividing the difference in the means of the variable in the 2 groups by an estimate of the standard deviation (SD). Larger values indicate that the 2 groups are dissimilar; a commonly recommended threshold value is <0.1.		
68	Patients with tibiotalocalcaneal (TTC) arthrodesis with dynamic or static compression intramedullary nails	Propensity score matching (PSM) was conducted to reduce confounding, creating a 1:1 matched cohort of SC and DC cases. Standardized mean differences of <0.1 were used to confirm balance between groups.	Adults	Observational study; Nationwide Readmissions Database (NRD)
69	(1) patients with a primary ACL partial or complete rupture who underwent subsequent ACLR, (2) patients with a minimum of 90 months (7.5 years) of follow-up, and (3) patients who gave consent for research.	The treatment effect of ACLR on the development of PTOA as well as progression to TKA was then evaluated using TMLE, a method to obtain valid statistical inference using MLMs. We performed TMLE using a Super Learner library that compiles the output from a diverse ensemble of algorithms (random forest, Xtreme gradient boosting, elastic net linear regression, and support vector machines) to generate an estimation of the treatment effect of ACLR on development of symptomatic PTOA.	Adults	Observational study; Rochester Epidemiology Project (REP)
70	Patients were included in this study if they had undergone primary, elective, unilateral DAA THA and had 1 year of follow-up.	To adjust for treatment/group selection bias and to match the cohorts, a logistic regression was used to calculate propensity scores for each patient based on age, sex, body mass index (BMI), femoral cementation, history of spine fusion, and Charlson Co-morbidity Index (CCI). The inverse probability of treatment weight (IPTW) was then calculated as the inverse of the propensity score. The IPTW adjusted demographics, preoperative, and surgical characteristics were reported as mean and standard deviations (SD) for continuous variables, and percentages for categorical variables. The IPTW was also applied to a propensity score-weighted regression model to compare the outcomes of complications and operation time between the groups.	Adults	Observational study; Author's Institution
71	Patients with adult spinal deformity (ASD) with a minimum 2-year follow-up who had undergone lower instrumented vertebrae to the sacrum without sacroiliac fusion were identified.	propensity score matching (PSM) was utilized, using nearest-neighbor and optimal matching algorithms to minimize selection bias when estimating causal treatment effects. Initially, univariate and multivariable logistic regressions were performed to identify independent factors associated with undergoing the 4PvS technique. Subsequently, logistic regression was performed to calculate individual propensity scores for each covariate. Treatment (4PvS) and control (2PvS) patients were matched at a 1:4 ratio according to these propensity scores.<br><br>To validate the generalizability of our findings, a sensitivity analysis using inverse probability of treatment weighting (IPTW) was conducted.	Adults	Observational study; Author's Institution
72	All patients who underwent unilateral primary or revision anterior cruciate ligament reconstruction (ACLR) with or without medial meniscal repair were eligible for enrollment.	To inform the statistical modeling necessary to compare these effects, a directed acyclic graph was created a priori to depict this framework and the temporal relationships among the features. Causal mediation analysis (CMA) addresses provides an analytical approach capable of distinguishing (1) the direct effect of an exposure from (2) the indirect effect of that same exposure through a mediator (or mediating event) on the ultimate outcome of interest. In doing so, CMA provides a more concrete description of the mechanism (or mechanisms) of how a singular intervention on a risk factor affects an outcome. The rationale, approach, and reporting of the current CMA follow the AGReMA statement (A Guideline for Reporting Mediation Analyses). The CMA was performed using R software (Version 4.2.3). A step-by-step approach to the CMA was employed such that step 1 determined whether the medial meniscal procedure significantly affected	Adults	Observational study; MOON database (Cleveland Clinic, The Ohio State University, Hospital for Special Surgery, Washington University at St Louis, University of Iowa, University of Colorado, and Vanderbilt University)

		the likelihood of subsequent surgery. Step 2 determined whether subsequent surgery affected the likelihood of KOOS pain <80 at 10-year follow-up, and step 3 determined if the effect of medial meniscal repair followed subsequent surgery and the direct effect of medial meniscal repair were significant.		
73	Patients between 18 and 80 years old with a diagnosis of HV with constant pain in the area of the first MTH not extending to other metatarsals, having particular discomfort while wearing shoes, and undergoing unilateral RIO or MIIND procedure.	To compare the two techniques, a propensity score matching (PSM) model was implemented. In this way, the impact of the two interventions and their outcomes could be estimated by accounting for differences in baseline characteristics, and other confounders and could be compared directly. PSM was carried out employing the commercial software XLSTAT (version for Windows OS, Lumivero) using an optimal algorithm based on the Euclidean distance, with a one-to-one match in the number of matches, and 0.10 * sigma as caliper size option. The propensity score was estimated using a logistic regression model in which the surgical treatment (RIO vs. MIIND) status was regressed on observed characteristics (covariates and factors).	Adults	Observational study; Author's Institution
74	The study population included patients who underwent a primary isolated ACLR. The population was restricted to patients aged ≤25 years and the use of HA at the index ACLR.	Propensity score weighting was used to balance covariates between comparison groups before outcome assessment. After applying propensity score weighting, the standardized mean difference (SMD) for each covariate was measured. An SMD of <0.2 indicates a balance achieved for the covariate between comparison groups. Propensity scores were calculated using a multivariable logistic regression model, which included all specified covariates as predictors of treatment assignment. Rosenbaum approach was used to address missing covariate values; we created separate levels for nominal variables with missing values and a missing indicator variable for continuous variables with missing data while also imputing the mean.	Adults	Observational study; Kaiser Permanente ACLR registry
75	Patients undergoing primary carpal tunnel release (CTR)	The present analysis incorporated the use of inverse-weighted propensity scores (IWPS) and absolute standardized differences such that a balanced comparison of patient characteristics across outcomes (revision surgery) could be performed. Characteristics identified as unbalanced were regressed upon index CTR type to generate propensity scores.	Adults	Observational study; Author's Institution
76	Patients diagnosed with end-stage hip osteoarthritis were eligible for the study when they fully completed a preoperative intake questionnaire, within 100 days before being placed on the THA waiting list.	To investigate the effect of THA surgery on hip disability and pain, we emulated the target trial using observational real-world data of 2 Dutch hospitals participating in the Santeon Better Together value-based health care (VBHC) program during the COVID-19 pandemic. Additionally, in the emulated trial, we adjusted for potential confounding factors measured at baseline to ensure comparability between the 2 groups.	Adults	Observational study; Author's Institution
77	Patients who underwent primary TKA for the diagnosis of osteoarthritis with a fully cemented Persona / NexGen TKA, a fully cementless Persona TM / NexGen TM (Zimmer Biomet), a fully cementless Triathalon, or fully cementless Triathlon Tritanium (Stryker Howmedica) implant.	Propensity score weighting was used to balance the distribution of covariates between the two study groups before revision risk assessment. Propensity scores were calculated using multivariable logistic regression models that included the covariates specified previously as predictors of treatment assignment. Propensity scores are the conditional probability of the patient receiving the treatment of interest given the covariates specified	Adults	Observational study; Kaiser Permanente Total Joint Replacement Registry (TJRR)
78	Patients undergoing revision total knee arthroplasty (rTKA) with stemmed components	Propensity score weighting was used to balance covariates between the groups before outcome assessment. The SMD for each covariate was measured before (unbalanced) and after (balanced) applying propensity score weighting; an SMD < 0.2 indicates balance between comparison groups for the covariate is achieved. Propensity scores were calculated using a multivariable logistic regression model that included all specified	Adults	Observational study; United States health care system's arthroplasty registry

		covariates as predictors of treatment assignment. Average treatment effect propensity score–weighted Cox proportional hazards regression was used to evaluate the risk of aseptic repeat revision by implant configuration. Robust standard errors were used to account for correlation from TKA performed by the same operating surgeon.		
79	Patients had to be 50 years or older, have unilateral or bilateral neurogenic claudication, with a life expectancy of more than one year, able to provide informed consent, and fluent in German. Patients with lumbar spinal stenosis with additional spondylolisthesis verified by magnetic resonance imaging (MRI) as a slippage of one vertebra over the adjacent one undergoing decompression with or without fusion within 6 months after enrollment in the study.	We described and emulated a hypothetical, pragmatic target trial mimicking a state-of-the-art index RCT comparing decompression with or without fusion for DS—the Norwegian Degenerative Spondylolisthesis and Spinal Stenosis trial (NORDSTEN-DS).<br>We used inverse probability weighting for confounding control and obtained balanced groups at baseline. Individual observations were weighted by the inverse of their probability of receiving decompression plus fusion surgery given a set of confounders.<br>The probability of receiving decompression plus fusion was obtained from a logistic regression model with fusion as dependent variable and the baseline covariates	Adults	Observational study; Author's Institution
80	Patients who were 18 to 80 years old at the time of surgery, diagnosed with femoroacetabular impingement (FAI), undergoing primary hip arthroscopy	The use of a historical cohort of patients whose labral treatment approach was not determined through randomization may give rise to systematic biases in the repair and reconstruction groups that could confound the comparison of outcomes. Thus, a 3-step modeling process, including multiple imputation of missing data, propensity score modeling, and multiple linear (or logistic) regression modeling with inverse propensity score weighting, was employed to estimate the average treatment effect in the treated (ATT)	Adults	Observational study; Registry
81	We identified adults aged ≥ 60 years with a main diagnosis of a femoral neck fracture.	Instrumental variable (IV) analysis was used to adjust for measured and unmeasured confounders to ensure comparability between patients who underwent THA and hemiarthroplasty. The differential distance was used for IV. We estimated the robustness of our findings by re-analyzing the primary outcomes using overlap weighting of the propensity score instead of IV analysis	Adults	Observational study; Diagnosis Procedure Combination database
82	Patients aged 16 years or older with femoral shaft fractures treated in the retrograde approach with either a SIGN Standard Nail or Fin Nail. Inclusion criteria included having a femoral fracture treated with a SIGN nail, either standard or fin nail, as well as a minimum of 6 months follow up.	A propensity score model and inverse probability of treatment weighted (IPTW) analysis were used to assess the relationship between IM nail used and painless weight bearing and radiographical union. Using IPTW weights, an average treatment effect is estimated, defined in our study the effect of Fin Nail usage assuming each patient had the opportunity to be treated with either nail.	Adults (>16)	Observational study; Sign Online Surgical Database (SOSD)
83	Patients who underwent single-level decompression of either L1–L2 or L2–L3 for the treatment of LSS. Inclusion criteria encompassed patients with a confirmed diagnosis of LSS in the upper lumbar spine necessitating single-level decompression.	To further evaluate the differences between the 2 groups, the inverse probability weighted logistic regression model was used. The authors calculated propensity scores based on 7 variables (the operated level, age, sex, body mass index, diabetes, smoking status, and ASA class ≥3), as in the previous studies. Then, the authors treated the inverse of the propensity score as a covariate and calculated the adjusted P values using following the inverse probability weighting method, which enable us to reduce the effect of these 7 variables for assuming the effect of each surgical method.	Adults	Observational study; 12 designated spine centers
84	Patients with symptomatic lumbar spinal stenosis confirmed by MRI	Propensity matching was achieved by utilizing the SPSS Python extension FUZZY version. The extension runs a logistic regression with the case/control group indicator as the dependent variable and the selected confounding variables as predictors. The resulting probabilities of estimated case group membership are then used to match each case from the control group. If an exact match cannot be made, a fuzzy distance algorithm (nearest neighbor) calculates the closest match based on a user defined tolerance level set between 0 (only exact matches) and 1 (any control	Adults	Observational study; Spine Centre of Southern Denmark and Elective Surgery Centre

		is a match). A control is deemed eligible to a match if the difference in the propensity scores is less than or equal to the tolerance level in absolute value.		
85	All patients aged 60 years or older having an elective primary shoulder replacement for osteoarthritis with an intact rotator cuff were eligible for inclusion in the study. Patients were included if they received a TSR or an RTSR.	Propensity scores were generated using logistic regression and represent the probability of a patient receiving an RTSR, as opposed to a TSR. One-to-one propensity score matching using callipers of width equal to 0.2 of the standard deviation of the logit of the propensity score was used to enable estimation of the average treatment effect on the treated population. Inverse probability of treatment weighting for participants on the common support of propensity scores was used for the average treatment effect on the total population.	Adults	Observational study; National Joint Registry of England, NHS Hospital Episode Statistics database

Table I
Evidence source details and characteristics charting table

ID	Aims/Purpose	Participants	Intervention Type	Comparator and Details	Outcomes and Details
1	To test whether an observational study employing propensity score matching could accurately estimate the causal treatment effects of rectus femoris transfer (RFT) as part of single-event multilevel surgery (SEMLS) in ambulatory children with cerebral palsy.	total 258 = 129 treated + 129 control (after matching)<br>129 limbs (81 males, average age 10 years 7 months) in the treated arm, 129 limbs (68 males, average age 10 years 2 months) in the control arm	Rectus femoris transfer (RFT) as part of single-event multilevel surgery (SEMLS)	SEMLS without RFT	The primary outcome measures were change (post - pre) scores in the key parameters related to stiff knee gait.
2	To quantify the causal effects of altered motor control and other impairments on gait, before and after single-event multi-level orthopedic surgery (SEMLS).	total 110 = 55 treated + 55 control<br>55 treated (35 males, age: 10.5 years) + 55 control (30 males, age: 10.0 years)	SEMLS	no SEMLS (i.e., single or no procedure)	Changes in Gait Deviation Index
3	To examine the relationship between surgery type and secondary hip surgery (revision or conversion arthroplasty) at 12 months following the index arthroplasty, and that between surgery type and dislocation at 12 months, among elderly Medicare beneficiaries who underwent THA or HA for a femoral neck fracture, taking into account the potential for selection bias.	total 61,695 = 10,268 THA + 51,427 HA<br>10,268 THA (2,896 males) + 51,427 HA (13,092 males)	Total hip arthroplasty (THA), Hemiarthroplast y (HA)	Total hip arthroplasty (THA), Hemiarthroplast y (HA)	Secondary hip surgery (revision or conversion arthroplasty) at 12 months following the index arthroplasty and dislocation at 12 months following the index arthroplasty.
4	To investigate the incidence rate and risk factors for subsequent revision in patients treated with HTO compared with those managed with TKA.	total 436,557 = 396,703 TKA + 39,854 HTO	High tibial osteotomy (HTO), total knee arthroplasty (TKA)	High tibial osteotomy (HTO), total knee arthroplasty (TKA)	Risk of revision (adjusted hazard ratio) and time to subsequent revision (Kaplan-Meier)
5	To clarify the effectiveness of preoperative physical therapy for older patients after hip fracture in an acute care hospital.	total 681 = 341 preoperative rehabilitation + 340 control	Preoperative physical therapy	No preoperative physical therapy (control group)	The primary outcome was motor Functional Independence Measure (FIM) gain. Secondary outcomes were motor-FIM effectiveness, motor FIM at discharge and discharge home.
6	To determine whether the addition of screws for uncemented acetabular component fixation influences acetabular component survival and THA survival when used with modern bearing surfaces.	total 330,192 = 104,862 THAs had one or more acetabular screws inserted + 225,330 had no screws	THA where the acetabular component was inserted with the addition of one or more screws	THA where the acetabular component was inserted with no screws	Risk of revision (hazard ratio)
7	To investigate the association between PLA and DAA for THA regarding the construct “activity and participation” (ICF model) during the first postoperative year.	total 860 = 238 PLA + 622 DAA	total hip arthroplasty (THA) posterolateral approach (PLA)	total hip arthroplasty (THA) direct anterior approach (DAA)	Questionnaires: Domain “activity and participation” was captured by validated, Dutch versions of the Hip Disability and Osteoarthritis Outcome Score (HOOS) Activities of daily living (ADL) and Sport and Recreation Function subscales
8	To compare the short-term revision risk in alternative surface bearing knees (oxidized zirconium (OZ) femoral implants or highly crosslinked polyethylene (HXLPE) inserts) ith that for traditional bearings (cobalt-chromium (CoCR) on conventional polyethelene (CPE)).	total 57,739 = 49,055 traditional + 7,618 CoCr-HXLPE + 1,066 OZ-CPE	Use of traditional bearings (CoCR-CPE) in total knee arthroplasty	Use of alternative bearings (CoCR-HXLPE or OZ-CPE) in total knee arthroplasty	Revision rates and risk of revision
9	To determine whether the use of	total 6,409 = 1,281	Primary elective	Primary elective	The primary outcome

	ABC in primary TSA is associated with a difference in the risk of deep infections.	ABC + 5,128 plain cement	anatomic TSA for osteoarthritis or reverse TSA (RTSA) for rotator cuff arthropathy with use of commercially prepared antibiotic-loaded bone cement (ABC)	anatomic TSA for osteoarthritis or reverse TSA (RTSA) for rotator cuff arthropathy with use of plain cement	evaluated was revision for deep infection within 5 years of the primary procedure (Hazard ratios).
10	To evaluate the effect of two-staged surgery on perioperative complications following ASD surgery using lateral lumbar interbody fusion (LLIF).	total 289 = 103 two-staged + 186 one-day	two-staged surgery using LLIF for ASD surgery	one-stage surgery	The primary outcome of interest was the occurrence of any systemic perioperative complication. Perioperative systemic complications (e.g., delirium; deep vein thrombosis; pulmonary embolism; cardiovascular events; respiratory, renal, or gastrointestinal complications; and stroke) were defined as events that required medical intervention or treatment within 30 days postoperatively. Secondary outcomes included the occurrence of reoperation within 30 days following ASD surgery due to surgery-related complications and intraoperative complications, operation time, intraoperative blood loss, transfusion, and length of hospital stay.
11	To compare the long-term revision rates of primary THA and HA for femoral neck fracture in order to determine whether unipolar or bipolar HA increases the all-cause risk of revision in patients 50 to 79 years old.	total 36,118 = 14,863 THA + 13,035 unipolar HA + 8,220 bipolar HA	Total hip arthroplasty (THA)	Hemiarthroplasty (HA)	Risk of revision (HR)
12	To compare the effectiveness of posterior lumbar in-terbody fusion (PLIF) using the cortical bone trajectory (CBT)and conventional pedicle screw (PS) techniques.	total 119 = 42 CBT + 77 PS	CBT-PLIF or PS-PLIF	CBT-PLIF or PS-PLIF	Operative time, intraoperative blood loss, and postoperative drainage, invasiveness of surgery, perioperative pain, total amount of diclofenac sodium suppositories given, Japanese Orthopaedic Association Back Pain Evaluation Questionnaire (JOABPEQ), visual analog scale (VAS), fusion status.
13	To compare the effectiveness of percutaneous needle aponeurotomy and limited fasciectomy for treating Dupuytren's contracture.	total 293 = 78 PNA + 215 LF	Percutaneous needle aponeurotomy (PNA)	Limited fasciectomy (LF)	The primary outcome was the degree of total residual extension deficit.<br>The impact of percutaneous needle aponeurotomy and limited fasciectomy on patient-reported hand function was assessed using the Michigan Hand Outcomes Questionnaire
14	To compare the surgical out-comes between bilateral partial laminectomy (BPL) and posterior lumbar interbody fusion (PLIF) in patients with mild degreeof slippage.	total 157 = 51 BPL + 106 PLIF	Between bilateral partial laminectomy (BPL)	Posterior lumbar interbody fusion (PLIF)	Operation time and intraoperative blood loss, Japanese Orthopedic Association (JOA) scoring system, recovery rate (JOA)
15	To compare clinical and radiographic outcomes of	total 29 = 15 D + 14 S	modified Dunn procedure (D) or	modified Dunn procedure (D) or	Nonarthritic Hip Score (NAHS) was used to assess

	modified Dunn procedure (D) and in situ fixation (S) in severe SCFE.		in situ fixation (S) for severe slipped capital femoral epiphysis	in situ fixation (S) for severe slipped capital femoral epiphysis	clinical and functional outcomes of patients at the latest follow-up. The PSA and the alpha angle on a frog-lateral view of the hip were used to assess the degree of correction and the radiographic presence of residual FAI deformity in the 2 groups. Rate of re-operation.
16	To compare long-term outcomes of anterior versus posterior lumbar interbody fusion	total 10,941 = 7,460 PLIF + 3,481 ALIF	anterior lumbar interbody fusion (ALIF) or posterior lumbar interbody fusion (PLIF)	anterior lumbar interbody fusion (ALIF) or posterior lumbar interbody fusion (PLIF)	Primary outcomes of interest were 2 year reoperation rates (presence of either a subsequent anterior or posterior interbodyfusion) and 90 day complication rates Secondary outcomes, any differences in time to reoperation, number of pain medications used in the postoperative period, amount of total postoperative healthcare utilization measured in hospital length of stay, outpatient clinic visits, total medication usage, and associated charges
17	To investigate whether use of a computed tomography (CT)-based navigation system reduce the risk of dislocation after total hip arthroplasty (THA) in patients with osteonecrosis of the femoral head (ONFH).	total 209 = 98 non-navigation + 111 navigation	Use of computed tomography (CT)-based navigation system	No navigation system	Japanese Orthopedic Association (JOA) score. Complications including dislocation, periprosthetic joint infection, and periprosthetic fracture. Radiological outcomes. Revision (Kaplan-Meier)
18	To compare knee laxity and functional scores 2 years after Dynamic intraligamentary stabilization (DIS) between patients with and without hardware removal. It is hypothesized that it does not affect ACL healing.	total 173 = 47 Hardware removal + 126 control	DIS with hardware removal	DIS without hardware removal	The primary outcome was the side-to-side difference in knee laxity measured with the rolimeter at manual maximum force (Δ-Lachman). Secondary outcomes were the pivotshift test and subjective scores.
19	(1) to investigate the independent risk factors for moderate to severe radiographic osteoarthritis, per the International Knee Documentation Committee (IKDC) guidelines, at 12 years of follow-up and (2) to compare the rate of the previously defined endpoint between the PT and HS groups	total 541 = 311 PT and 230 HS	Anterior cruciate ligament (ACL) reconstruction with patellar tendon (PT) or hamstring tendon (HS) graft	Anterior cruciate ligament (ACL) reconstruction with patellar tendon (PT) or hamstring tendon (HS) graft	The primary endpoint was the presence of moderate to severe osteoarthritis at 12 years of follow-up, with use of the worst IKDC score from the radiographs. The secondary endpoints included clinical evaluations by the subjective IKDC score and the KOOS.
20	To clarify if (1) surgery may directly affect overall survival (OS) aside from short-term neurologic outcome, (2) explore whether selected patient subgroups with poor mBs might still benefit from surgery, and (3) gauge putative adverse effects of surgery on short-term oncologic outcomes.	total 398 = 194 surgery + 204 no surgery	Decompressive surgery with dorsal stabilization and subsequent recommendation of radiation	No surgery	Risks of death-from any-cause (overall survival) and progression-free survival were estimated with 1-Kaplan–Meier estimators Risks of radiographic disease progression were estimated with competing risk cumulative incidence estimators
21	To compare the outcomes for salvage-treated patients at 18 months with the outcomes that these patients would have experienced had they undergone early amputation.	total 639 = 488 limb salvage + 151 early amputation	early amputation	limb salvage	The primary outcome was the Short Musculoskeletal Function Assessment (SMFA). Secondary outcomes included pain as measured by the Brief Pain Inventory

					(BPI), participation in vigorous activities as measured by the Paffenbarger Physical Activity Questionnaire (PPAQ), and return to work (by those working prior to injury).
22	To compare patient outcomes and complication rates after different decompression techniques or instrumented fusion (IF) in lumbar spinal stenosis (LSS)	total 1,176 = 642 laminotomy + 196 hemilaminectomy + 230 laminectomy + 108 laminectomy combined with an instrumented fusion (IF)	(1) laminotomy, (2) hemilami-nectomy, (3) laminectomy, (4) laminectomy combined with an instrumented fusion (IF)	(1) laminotomy, (2) hemilami-nectomy, (3) laminectomy, (4) laminectomy combined with an instrumented fusion (IF)	Comparison of pain relief and Core Outcome Measure Index (COMI) score improvement between baseline and follow-up was performed in each group using the Wilcoxon signed-rank test.<br>As clinical dichotomized outcomes, achievement of minimum relevant change (MRC) in (a) COMI back and (b) leg pain and (c) COMI score of 2.2 points on the GPS, (d) surgical and (e) general complications, (f) measures taken due to complications, and (g) reintervention on the index level based on patient information (COMI) were considered.
23	To investigate the subsequent cardiovascular risk of patients with knee osteoarthritis underwent total knee arthroplasty in Taiwan.	total 22,931 = 15,363 TKA + 7568 conservative	total knee arthroplasty (TKA)	conservative treatment	cardiovascular disease (CVD)
24	To compare the changes in radiographs between subjects treated with APM and those treated with PT in the Meniscal Tear.	total 142 = 100 APM + 42 PT	arthroscopic partial meniscectomy (APM)	physical therapy (PT)	Changes between baseline and 60 months in the Kellgren-Lawrence (KL) grade and OARSI radiographic score
25	To compare revision rates following primary hemiarthroplasty for femoral neck fracture to determine if the unipolar hemiarthroplasty design increases the risk of revision arthroplasty for all causes	total 62,875 = 41,158 unipolar + 21,717 bipolar	Unipolar or Bipolar Hemiarthroplast y	Unipolar or Bipolar Hemiarthroplast y	The primary outcome was time to revision for any cause.<br>Secondary analyses were performed on the reason for revision(infection, dislocation, periprosthetic fracture, or acetabularerosion), the use of femoral stemfixation, and the type of stem (polished or matte).
26	To investigate the incidence rate and risk factors for subsequent revision in patients treated with UKA compared with those treated with HTO.	total 72,184 = 22,433 UKA + 49,751 HTO	high tibial osteotomy (HTO) or unicompartment al knee arthroplasty (UKA)	high tibial osteotomy (HTO) or unicompartment al knee arthroplasty (UKA)	Incidence rate and risk factors of newly acquired subsequent revision
27	To compare rates of intraoperative fracture and complications requiring reoperation within 1 year for posterior approach RA-THA, CN-THA, and THA with no technology (Manual-THA)	total 13,802 = 1,770 RA-THAs + 3,155 CN-THAs + 8877 Manual-THA	Robotic-assistance total hip arthroplasty (RA-THA) or computer navigation THA (CN-THA) or no technology (Manual-THA)	Robotic-assistance total hip arthroplasty (RA-THA) or computer navigation THA (CN-THA) or no technology (Manual-THA)	Intraoperative fractures and reoperations within 1 year of the index procedure
28	To compare the postoperative surgical site infection (SSI) rate between PF and VD in open Achilles's tendon repair surgery.	total 60 = 30 PF + 30 VD	Posterior crural fasciotomy (PF)	inserted vacuum suction drainage (VD)	The primary outcome was SSI rate.<br>Secondary outcomes were Achilles tendon total rupture score (ATRS), functional foot index, and visual analogue scale.

29	To determine the association between 30-day mortality and choice of surgical fixation, comparing short IM nail, long IM nail and SHS fixation, for patients aged 50 years and over presenting with intertrochanteric fractures. The secondary aim was to analyse this association over the first year following surgery.	total 16,677 = 7300 IM nail short + 4251 IM nail long + 5126 SHS	Short intramedullary (IM) nail, long IM nail and sliding hip screw (SHS) fixation	Short intramedullary (IM) nail, long IM nail and sliding hip screw (SHS) fixation	The primary outcome was mortality within 30-days of surgery and the secondary outcome was mortality within 1-year of surgery
30	(1) to replicate the Total or Partial Knee Arthroplasty Trial (TOPKAT), a surgical randomised controlled trial comparing unicompartmental knee replacement with total knee replacement using propensity score and instrumental variable methods. (2) to compare the risk benefits and cost- effectiveness of unicompartmental knee replacement with total knee replacement surgery in patients with severe systemic morbidity who would have been ineligible for TOPKAT using the validated methods from (1).	total 127,031 = 125,834 TKR + 1197 UKR	unicompartmental knee replacement surgery (UKR) or total knee replacement surgery (TKR)	unicompartmental knee replacement surgery or total knee replacement surgery	The primary outcome measure was the postoperative Oxford Knee Score. The secondary outcome measures were 90-day postoperative complications (venous thromboembolism, myocardial infarction and prosthetic joint infection) and 5-year revision risk and mortality. The main outcome measures for the health economic analysis were health-related quality of life (EuroQol-5 Dimensions) and NHS hospital costs.
31	To assess pain and functional outcomes in patients undergoing operative versus non-operative treatments for rotator cuff tears.	total 127 = 77 non-operative + 50 operative	Operative versus non-operative treatments for rotator cuff tears	Operative versus non-operative treatments for rotator cuff tears	Shoulder Pain and Disability Index (SPADI), the American Shoulder and Elbow Surgeons Standardized Form (ASES); ratio of the affected shoulder versus the contralateral shoulder strength; MRI readings
32	(1) Do primary TKAs with HXLPE tibial inserts have a lower risk of revision (all-cause, aseptic, and septic) than TKAs with conventional polyethylene tibial inserts? (2) In NexGen TKA (Zimmer Inc, Warsaw, IN, USA) bearings, do HXLPE tibial inserts have a lower risk of revision (all-cause, aseptic, and septic) than conventional polyethylene tibial inserts? (3) In Press-Fit Condylar TKA (PFC or PFC Sigma; DePuy Inc, Warsaw, IN, USA), do HXLPE tibial inserts have a lower risk of revision (all-cause, aseptic, and septic) than procedures performed with conventional polyethylene tibial inserts?	total 71,889 = 11,048 HXLPE + 60,841 CPE	Highly crosslinked polyethylene (HXLPE)	conventional polyethylene (CPE)	Revision risk
33	(1) Do primary THAs with a metal-on-conventional polyethylene bearing surface have a higher risk of revision (all-cause or aseptic) than metal-on-HXLPE? (2) Is the risk of revision (all-cause or aseptic) higher for conventional polyethylene versus HXLPE when the effect of femoral and acetabular components is controlled for in prosthesis-specific analyses.	total 26,823 = 1,815 CPE + 25,008 HXLPE	Highly crosslinked polyethylene (HXLPE)	conventional polyethylene (CPE)	Revision risk
34	To evaluate the outcomes of two commonly used transosseous-equivalent (TOE) arthroscopic rotator cuff repair (RCR)	total 130 = 33 KSB + 97 KTB	Knotted suture bridging (KSB) or a knotless tape bridging	Knotted suture bridging (KSB) or a knotless tape bridging	ASES and SF-12 PCS scores assessed at a minimum of 2 years postoperatively

	techniques for full-thickness supraspinatus tendon tears (FTST) using a robust multi-predictor model.		(KTB)	(KTB)	
35	To compare the survival of total knee arthroplasty (TKA) converted from unicompartmental knee arthroplasties (UKA) with TKA converted from high tibial osteotomies (HTO)	total 2,133 = 978 UKA + 1,155 HTO	TKA following unicompartment al knee arthroplasties (UKA) or TKA following high tibial osteotomies (HTO)	TKA following unicompartment al knee arthroplasties (UKA) or TKA following high tibial osteotomies (HTO)	Revision risk
36	To assess the prospective, longitudinal outcome after arthroscopically assisted open reduction and internal fixation (AORIF) and to compare the results with open reduction and internal fixation (ORIF) in complex ankle fractures.	total 50 = 25 + 25	Assisted open reduction and internal fixation (AORIF)	Open reduction and internal fixation (ORIF)	Olerud and Molander Ankle Score (OMAS), Foot and Ankle Ability Measure (FAAM), ADL and Sports subscales;<br>secondary: Tegner Activity Scale (TAS for AORIF only)
37	To compare clinical outcomes between isolated lateral closing wedge distal femoral osteotomy (lcwDFO) and medial opening wedge high tibial osteotomy (mowHTO), performed according to deformity location	total 50 = 25 + 25	Isolated lcwDFO	Isolated mowHTO	Outcome measures included the Western Ontario and McMaster Universities Osteoarthritis Index (WOMAC), the Lysholm score, the International Knee Documentation Committee (IKDC) Subjective Knee Form, the Tegner Activity Scale, and the visual analog scale (VAS) for pain.
38	To evaluate perioperative outcomes, radiological accuracy of prosthesis placement, and early clinical outcomes of SkyWalker robot-assisted TKA compared to conventional TKA	total 156 = 52 RATKA + 104 TKA	Robot-assisted Total knee arthroplasty (RATKA)	Total knee arthroplasty (TKA)	operation time, tourniquet time, length of hospital stay, intraoperative bleeding, perioperative hemoglobin (Hb) level (before surgery, at 1, 3 days after surgery) and Western Ontario and McMaster Universities (WOMAC) scores (before and three months after surgery), postoperative alignment and components position
39	To assess whether patients with knee osteoarthritis and whom undergo arthroscopic meniscectomy have an increased risk for future knee replacement surgery.	total 670 = 335 + 335	Arthroscopic meniscectomy	Control	Time to knee replacement
40	To investigate the incidence rate and risk factors of subsequent revision in patients with UKA and TKA.	total 418,711 = 397,381 TKA + 21,330 UKA<br>(before matching)	Unicompartment al knee arthroplasty (UKA)	Total knee arthroplasty (TKA)	Revision
41	To investigate the long-term efficacy of artificial disc replacement (ADR) surgery compared with fusion after decompression for the treatment of cervical degenerative disc disease and radiculopathy in a population-based setting.	total 370 = 185 + 185	anterior decompression and insertion of an Artificial disc replacement (ADR) vs anterior decompression combined with fusion surgery.	anterior decompression and insertion of an Artificial disc replacement (ADR) vs anterior decompression combined with fusion surgery.	The primary outcome (i.e., the NDI score) is a 10-item self-administered questionnaire measuring disability in patients with neck pain.<br>Secondary outcomes were the EQ-5D scores (ranging from −0.5 to 1, with higher scores reflecting a better quality of life), EQ-5D health scores (ranging from 0 to 100, with higher scores indicating better health), and VAS scores for neck and arm pain (ranging from 0 to 10, with higher scores indicating more severe pain).
42	To compare the short-term patient-reported outcome	total 168 = 84 + 84	Lumbar discectomy	Lumbar discectomy	The primary outcomes were (1) preoperative PROMIS

	improvements (value) of DSC vs. ACLR using PROMIS scores		(DSC) vs anterior cruciate ligament reconstructions (ACLR)	(DSC) vs anterior cruciate ligament reconstructions (ACLR)	domain scores, (2) scores at a minimum of 40 days postoperatively for DSC patients and 133 days postoperatively for ACLR patients, and (3) the change in scores with surgery.
43	To elucidate trends in the utilization of intraoperative neuromonitoring (IONM) during anterior cervical discectomy and fusion (ACDF) procedures in NY state using the Statewide Planning and Research Cooperative System and to determine if utilization of IONM resulted in a reduction in postoperative neurological deficits.	total 27,008 = 13,504 + 13,504	intraoperative neuromonitoring (IONM) + anterior cervical discectomy and fusion (ACDF)	anterior cervical discectomy and fusion (ACDF)	postoperative neurological deficits<br>secondary outcomes of length of stay (continuous variable), any in-hospital complication, nerve root or cord injury, and any in-hospital complication except nerve root injury and cord injury.
44	To evaluate the effect of acellular dermal matrix (ADM) on surgery of recurred and neglected patients of congenital muscular torticollis.	total 49 = 18 ADM + 31 Non-ADM	unipolar release + Acellular dermal matrix (ADM)	unipolar release	Passive range of motion (PROM) of neck<br>Rotational deficit, Lateral flexion deficit<br>scar outcome of the neck incision at the 1-year follow-up using the Vancouver scar scale (VSS)<br>Residual band<br>Patient satisfaction (cosmetic and functional)<br>Head tilt
45	To compare the clinical results of revision interbody fusion surgery between lateral lumbar interbody fusion (LLIF) and posterior lumbar interbody fusion (PLIF) or transforaminal lumbar interbody fusion (TLIF) with propensity score (PS) adjustments and to investigate the efficacy of indirect decompression with LLIF in previously decompressed segments on the basis of radiological assessment.	total 56 = 21 LLIF + 35 PLIF/TLIF	lateral lumbar interbody fusion (LLIF)	posterior lumbar interbody fusion (PLIF) or transforaminal lumbar interbody fusion (TLIF)	1) the incidence of postoperative complications, including incidental dural tear, and SSI; onset of neurological deficits, including motor and sensory symptoms; endplate injury; and reoperation within 12 months for any reason;<br>2) operative factors such as estimated blood loss (EBL) and operative time;<br>3) postoperative improvement in JOA score for lumbar diseases.
46	To test whether laminectomy + fusion reduces 2-year risk of subsequent surgery (SS) compared with laminectomy alone	total 3,843 = 2,212 laminectomy + 1,631 LF	laminectomy vs laminectomy with instrumented lumbar fusion (LF).	laminectomy vs laminectomy with instrumented lumbar fusion (LF).	Subsequent surgery (SS) within 2 years;<br>Complication-related diagnoses, defined as spinal hematoma, lumbar radiculopathy, spinal fluid leak, postlaminectomy syndrome, and pseudoarthrosis, were counted per group.
47	To investigate how short- and long-term mortality differs between those who have been operated with hemiarthroplasty or pins/screws.	total 2,932 = 1,466 + 1,466	hemiarthroplasty vs pins/screws	hemiarthroplasty vs pins/screws	Mortality (30 day post-OP); secondary: 1-year post-OP mortality, length of stay
48	To quantify the increase in venous thromboembolic events (VTEs) attributable to anterior approach lumbar fusions compared to posterior approach	total 1,058 = 187 anterior + 871 posterior	Anterior lumbar interbody fusion (ALIF) (with or without posterior instrumentation under same anesthesia)	Posterior approach lumbar fusion (TLIF, PLIF, or posterior-only fusion)	Any venous thromboembolic event (VTE), defined as either deep venous thrombosis (DVT) or pulmonary embolism (PE), that was diagnosed within 30 days postoperatively
49	To determine the effectiveness of commercially available ABC in reducing the risk of infection following TKA, both overall and among 3 subgroups of patients with a higher risk of	total 87,018 = 27,539 Antibiotic Cement + 59,479 Regular Cement	Primary Total Knee Arthroplasty + commercially prepared antibiotic-loaded	Primary Total Knee Arthroplasty + cement without antibiotics	The primary outcome was the time to first infection. The date of infection was defined as the date at which a patient was either diagnosed within 90 days postoperatively with

	infection (diabetes, body mass index ≥35 kg/m2, and American Society of Anesthesiologists classification ≥3), and to evaluate the association between the use of ABC and the risks of aseptic revision and revision for aseptic loosening.		bone cement (ABC)		a deep infection or underwent a septic revision procedure, whichever came first.<br><br>Secondary outcomes included the time to aseptic revision and to revision for aseptic loosening. Aseptic revision was defined as any reoperation performed after the index TKA in which an implant was exchanged for aseptic reasons.
50	To compare the rate of any reoperation for infection (both superficial and deep) in primary total hip arthroplasty (THA) and primary total knee arthroplasty (TKA) among patients who did and did not receive PI irrigation prior to wound closure.	total 11,738 patients = 5,534 THA (PI 1,322; no-PI 4,212) + 6,204 TKA (PI 2,410; no-PI 3,794)	THA or TKA + Povidone-iodine (PI) irrigation	THA or TKA	The primary outcome measure was any reoperation for infection, including superficial wound infections and deep PJIs. All outcome variables were evaluated at 3 months and 1 year following the primary TJA.
51	To compare in-hospital mortality of three procedures – halo-vest immobilization, anterior spinal fixation (ASF), and posterior spinal fixation (PSF)– in the treatment of elderly patients with isolated C2 odontoid fracture.	total 891 = 463 halo-vest + 74 ASF + 354 PSF	halo-vest immobilization vs anterior spinal fixation (ASF) vs posterior spinal fixation (PSF)	halo-vest immobilization vs anterior spinal fixation (ASF) vs posterior spinal fixation (PSF)	The primary endpoint was overall in-hospital mortality. The secondary endpoints were at least one complication after admission, post-treatment length of stay (PLOS), and total hospitalization cost in US dollars (USD).
52	To estimate reoperation rate after MED at median and to compare revision rates (long-term and 90-day) between MED and MD/OD.	total 1968 = 646 microendoscopic discectomy + 1,322 microdiscectomy or open discectomy	Microendoscopic discectomy (MED)	Microdiscectomy or open discectomy (MD/OD)	The primary outcome was the incidence of any type of second lumbar spine surgery before the end of the follow-up period.<br>The secondary outcome was the incidence of any lumbar surgery within 90 days after the index date because, in general, the reason for reoperation before versus after 90 days may differ.
53	To compare rib fixation with non-operative treatment in patients with multiple rib fractures without a flail chest.	total 142 = 71 + 71	Nonoperative vs Rib fixation	Nonoperative vs Rib fixation	The primary outcome measure was hospital length of stay (HLOS).<br>Secondary outcomes included intensive care unit length of stay (ILOS), duration of mechanical ventilation (DMV), need for tracheostomy, pneumonia rate and other in-hospital complications, in-hospital mortality rate, and general pain (measured using a numeric rating scale [NRS]).<br>Mid- and long-term outcomes were measured at the outpatient clinic visit at 6 weeks and using telephone interviews after 12 months.
54	To compare midline lumbar interbody fusion (MIDLIF) and minimally invasive transforaminal lumbar interbody fusion (MIS-TLIF) for treatment of patients with severe stenosis and lumbar degenerative spondylolisthesis (DS), focusing on dural tears rates, other complications, clinical and radiological outcomes.	total 72 = 36 + 36	midline lumbar interbody fusion (MIDLIF) vs minimally invasive transforaminal lumbar interbody fusion (MIS-TLIF)	midline lumbar interbody fusion (MIDLIF) vs minimally invasive transforaminal lumbar interbody fusion (MIS-TLIF)	The primary endpoint was the rate of dural tears, in order to study the effect of a more straightforward decompression with MIDLIF.<br>Secondary endpoints were clinical outcome according to Odom's criteria and lumbar lordosis improvement.
55	To compare the clinical outcomes of patients who received operative treatment for	total 214 = 107 + 107	Surgical Repair vs Nonsurgical Treatment	Surgical Repair vs Nonsurgical Treatment	The Normalized Western Ontario Rotator Cuff Index (WORCnorm) was the

	a rotator cuff tear with the outcomes of patients who had nonoperative management.				primary outcome in this study, while the ASES, SANE, and pain VAS were secondary outcomes.
56	To evaluate whether spinopelvic alignment modifies patient-reported and clinical outcomes after decompression vs. decompression and fusion	total 204 = 102 + 102 (low missmatch)<br>total 256 = 128 + 128 (high missmatch)	Lumbar decompression and fusion	Lumbar decompression	The primary outcome was the change in Patient-Reported Outcome Measurement Information System (PROMIS), Global Physical Health (GPH), and Global Mental Health (GMH) scores at baseline and post-operatively at 4-6 and 10-12 months postoperatively. Secondary outcomes included operative parameters (estimated blood loss and operative time), and two-year clinical outcomes including reoperations, duration of postoperative physical therapy, and discharge disposition.
57	To compare the 30-day perioperative complications of multilevel ACDF versus LAMP for the treatment of CSM.	total 1,242 = 621 + 621	Multilevel anterior cervical discectomy and fusion (ACDF)	Laminoplasty	complications, as well as the outcome measures of hospital length of stay (HLOS) and discharge destination
58	To compare clinical outcomes and cost-effectiveness with bone substitutes on volar locking plate (VLP) fixation of unstable distal radial fractures (DRF) in the elderly.	total 485 = 388 VLA + 97 VLS	volar locking plate (VLP) fixation alone (VLA)	volar locking plate (VLP) fixation with bone substitutes (VLS)	modified Mayo wrist score (MMWS), implant failure rate, bone union rate, volar tilt (VT), radial inclination (RI), ulnar variance (UV) and DDD, cost
59	To determine if the anterior approach (AP) influenced the incidence of early PJI in THA compared to posterior approach (PP).	total 25,210 = 12,605 + 12,605	total hip arthroplasty (THA) anterior approach (AP) vs THA posterior approach (PP)	total hip arthroplasty (THA) anterior approach (AP) vs THA posterior approach (PP)	90-day PJI, readmission, revision
60	To determine if a pre- and postoperative physiotherapy program delivered via a digital application could reduce hospital length of stay (LOS) without compromising patient outcomes.	total 186 = 124 DP + 62 CP	Digital Protocol (DP) of postoperative rehabilitation	Conventional Protocol (CP) of postoperative rehabilitation	postoperative Knee Injury and Osteoarthritis Outcome Score (KOOS), KOOS for Joint Replacement (KOOS, JR), and acute, rehabilitation, and total LOS
61	To investigate the perioperative complications of HA and THA in femoral neck fracture, using a large nationwide inpatient database.	total 9,934 = 4,967 + 4,967	Total Hip Arthroplasty (THA)	Hemiarthroplasty (HA)	30 day in-hospital mortality and postoperative complications: Postoperative complications contained both systemic complications and local complications. The following complications—coronary heart disease, heart failure, respiratory disorders, pulmonary embolism, stroke, renal failure, urinary tract infection, sepsis, intensive care unit (ICU) admission, and blood transfusion—were included as systemic complications, and we defined revision surgery, hip dislocation, surgical site infection, and debridement procedure as local complications.<br>Revision surgery as a surgery coded as hemiarthroplasty (K0811) or total hip arthroplasty (K0821) after the HA or THA index.<br>Anesthesia time, length of hospital stay, and daily

					average medical cost.
62	To estimate the effect of surgery on mortality after isolated acute traumatic axis fracture in older adults.	total 2,320 = 223 surgery + 2,097 no surgery	Surgical management performed during index hospitalisation	Nonsurgical management	all-cause 1-year mortality; in-hospital mortaliyt (30, 180, 360 days)
63	To compare the 5-year clinical outcome results in Neck Disability Index (NDI) in patients with cervical degenerative disc disease and radiculopathy who were treated with either posterior foraminotomy or ACDF surgery.	total 1,140 = 570 + 570	posterior foraminotomy (PF) vs anterior cervical decompression and fusion (ACDF)	posterior foraminotomy (PF) vs anterior cervical decompression and fusion (ACDF)	Neck Disability Index (NDI); secondary: EQ-5D, pain (arm, neck), reoperations, surgical complications
64	To emulate the design of the Total or Partial Knee Arthroplasty Trial (TOPKAT) using routinely collected data to assess whether the efficacy results reported in the trial translate into effectiveness in routine practice, and to assess comparative safety.	total 282,756 = 32,379 UKR + 250,377 TKR	unicompartment al knee replacement (UKR) vs total knee replacement (TKR)	unicompartment al knee replacement (UKR) vs total knee replacement (TKR)	The primary outcome in TOPKAT was patient-reported pain and function. As these data were not available in our study, we assessed the effect of type of procedure (UKR or TKR) on opioid use, as a proxy for persistent pain. Secondary outcomes in the TOPKAT trial included postoperative complications and implant survival, and these were also assessed in our study.
65	To compare the improvement of neurological deficits and pain 4 months after treatment between patients with HPVO treated medically alone or treated medically and surgically, using a propensity score. The secondary objective was to evaluate the outcome of the two treatments in terms of infection-related complications such as recurrences and infection-related deaths at 12 months.	total 90 = 62 MT + 28 MST	medical treatment (MT) vs medical and surgical treatment (MST)	medical treatment (MT) vs medical and surgical treatment (MST)	Neurologic deficit (using the ASIA impairment scale) and pain (using the analgesic level required) 4 months later, recurrences and infection-related deaths 12 months later
66	To compare TAA and AA outcomes using a national dataset to inform patient-centered care.	total 13,660 = 6,830 + 6,830	Total ankle arthroplasty (TAA) vs ankle arthrodesis (AA)	Total ankle arthroplasty (TAA) vs ankle arthrodesis (AA)	mortality, LOS, mean total charges, complitcations
67	To investigate Computer navigation and patient-specific instrumentation influence on implant survival, outcomes of the Oxford Knee Score (OKS) and health-related quality of life (EQ-5D-3L), intraoperative complications, and postoperative mortality compared with conventional instrumentation, across a real-world population.	total 1,064,010 = 1,031,950 TKR + 27,161 computer navigation + 4,899 patient-specific	TKR + computer navigation vs TKR + patient-specific instrumentation vs TKR	TKR + computer navigation vs TKR + patient-specific instrumentation vs TKR	Revision for all causes secondary: Oxford knee score, mortality, intraoperative complications
68	To compare nonunion and complication rates between dynamic compression (DC) and static compression (SC) nails in Tibiotalocalcaneal (TTC) arthrodesis using the Nationwide Readmissions Database.	total 311 = 149 DC + 162 SC	dynamic compression (DC) vs static compression (SC)	dynamic compression (DC) vs static compression (SC)	Nonunion, complications (infection, implant-related issues); readmission rates
69	To compare the risk of posttraumatic knee osteoarthritis (PTOA) between patients treated without surgery and with anterior cruciate ligament (ACL) reconstruction (ACLR) after primary ACL disruption using a machine learning causal inference model.	total 1,194 = 974 ACLR + 220 Non-operative	anterior cruciate ligament reconstruction (ACLR)	Non-operative treatment	posttraumatic knee osteoarthritis (PTOA)

70	To compare intraoperative fracture and return to the operating room within 1 year of index direct anterior approach (DAA) total hip arthroplasty (THA) performed with robotic-assistance (RA-THA), computer-navigation (CN-THA), and only fluoroscopy (Manual-THA).	total 3,433 = 226 RA-THA + 1007 CN-THA + 2200 Manual-THA	Robotic-assisted (RA-THA) vs computer navigated (CN-THA) vs Manual-THA	Robotic-assisted (RA-THA) vs computer navigated (CN-THA) vs Manual-THA	Intraoperative fractures: any discharge weight-bearing status other than "weight-bearing as tolerated", any mention in the operative notes of a complication, and any surgical intraoperative time that exceeded 2 standard deviations of the mean.<br><br>Postoperative complications requiring revision: all readmissions and re-operations within 1 year of the index surgery were reviewed to assess if re-operation was related to the index procedure. Only re-operations occurring within 1 year were considered. Readmissions not resulting in re-operation, medical complications, or complications not requiring re-operation were not included.
71	To compare a multiple pelvic screw fixation strategy (dual bilateral 4 pelvic screw fixation [4PvS]) with the use of single bilateral 2 pelvic screw fixation (2PvS), with the aim of addressing lumbosacral junction stability.	total 406 = 349 2PvS + 57 4PvS	posterior spinal fusion (PSF) + 4PvS	posterior spinal fusion (PSF) + 2PvS	Spinal implant-related reoperation: implant displacement events such as screw malposition, loosening, or pullout, as well as implant breakage incidents involving rod fractures (below L4) or pelvic screw fractures. An additional primary outcome was the incidence of pelvic screw breakage, whether asymptomatic or symptomatic.<br><br>Secondary outcomes encompassed various surgical complications and patient-reported outcomes (PROs) using the Scoliosis Research Society (SRS)–22r questionnaire. These included the overall rate of intraoperative complications, massive blood loss, lymphocele, dural tear, vascular injury, perioperative neurological deterioration, perioperative sensory deficit, perioperative motor deficit, and perioperative bowel and bladder deficit.
72	To determine if medial meniscal repair decreases Knee injury and Osteoarthritis Outcome Score (KOOS) pain 10 years after primary anterior cruciate ligament reconstruction (ACLR) and to assess the consequences of subsequent surgery on the development of KOOS pain.	total 776 = 396 excision + 380 repair	ACLR + medial meniscal repair vs ACLR + medial meniscal excision	ACLR + medial meniscal repair vs ACLR + medial meniscal excision	KOOS (knee pain score)
73	To compare the clinical and radiographic outcomes of the Reverdin-Isham osteotomy (RIO) and the Minimally Invasive Intramedullary Nail Device (MIIND) surgical techniques.	total 60 = 30 RIO + 30 MIIND	RIO vs MIIND	RIO vs MIIND	Radiological outcomes were evaluated using the MedStation program (Version 4.9). Hallux valgus angle (HVA), intermetatarsal angle (IMA), distal metatarsal articular angle (DMAA), and the tibial sesamoid position (TSP)

					were measured and categorised with regard to deformity severity. The 100-point hallux metatarsophalangeal-interphalangeal scale by the American Orthopaedic Foot and Ankle Society (AOFAS) was used to assess clinical outcomes. pain was evaluated using the Numeric Rating Scale (NRS-11). Patient satisfaction was evaluated using the Visual Analogue Scale (VAS) for satisfaction, ranging from 0 (not satisfied) to 10 points (excellent result).
74	To evaluate the risk of aseptic revision after Hamstring autograft (HA) for anterior cruciate ligament reconstruction (ACLR) (HA-ACLR) in patients aged ≤25 years, comparing graft diameters of (1) <8 versus ≥8 mm and (2) ≤8 versus >8 mm. A secondary purpose was to determine whether there was a threshold for graft diameter that best identifies patients at a higher risk of aseptic revision.	total 5,972 = 1,397 <8mm + 4,575 ≥8mm OR 3,628 ≤8mm + 2,344 >8mm	(1) <8 versus ≥8 mm and (2) ≤8 versus >8 mm HA diameter	(1) <8 versus ≥8 mm and (2) ≤8 versus >8 mm HA diameter	The primary outcome was aseptic revision surgery, defined as any reoperation performed after the index ACLR where removal and replacement of the original graft for aseptic reasons was required. Within our health care system, patients are continuously monitored for revisions after the index ACLR through surgeon reports and EHR surveillance until either health care plan membership termination or death.
75	To compare rates of revision surgery between primary endoscopic carpal tunnel release (ECTR) and open carpal tunnel release (OCTR). In addition, we aimed to assess the influence of fellowship training on revision rates.	total 4,160 = 787 ECTR + 3,373 OCTR	Endoscopic carpal tunnel release (ECTR) vs Open carpal tunnel release (OCTR)	Endoscopic carpal tunnel release (ECTR) vs Open carpal tunnel release (OCTR)	12-month revision rates for ECTR and OCTR.<br><br>Secondary outcome: overall revision rates for OCTR between fellowship-trained hand surgeons and nonhand surgeons.
76	To assess the causal effects of total hip arthroplasty (THA) compared with nonoperative treatment in reducing patient-reported hip disability at 3 months in patients with osteoarthritis, using real-world data.	total 373 = 146 THA + 227 Non-THA	Elective primary THA	Non-THA	Clinical outcomes on 3 domains as outlined by the OMERACT-OARSI Core Domain Set were measured using validated patient-reported outcome measures (PROMs): physical function using HOOS-PS ranging from 0 to 100 with lower scores reflecting better physical function, pain during weightbearing using the numerical rating scale (NRS), and the patient's global assessment of the target joint using an anchor-based question. The anchor-based question was administered for improvement in hip function and hip pain, consisting of a 7-item Likert scale from "very much improved" as the best option available and "very much deteriorated" as the worst option available.
77	(1) Is there a difference in aseptic survivorship between cementless twin-peg TKA constructs compared with cemented options? (2) Is there a difference in aseptic survivorship between	total 130,242 = 125,414 Twin-peg construc + 4,828 Spikes-and-keel construct	(1) cementless twin-peg TKA vs cemented (2) cementless spikes-and-keel TKA vs cemented	(1) cementless twin-peg TKA vs cemented (2) cementless spikes-and-keel TKA vs cemented	Aseptic revision surgery after the index TKA. This was procedurally based, defined as any operation after the primary TKA in which any (tibia, femur, liner, or patella) implant component was

	cementless spikes-and-keel TKA constructs compared with cemented options? (3) Is there a difference in aseptic survivorship between cementless twin-peg TKA constructs compared with cementless spikes-and-keel TKA constructs?		(3) cementless twin-peg TKA vs cementless spikes-and-keel TKA	(3) cementless twin-peg TKA vs cementless spikes-and-keel TKA	removed and replaced for noninfectious reasons.<br><br>Secondary outcome: revision, specifically of loosening, instability, and periprosthetic fractures.
78	To compare re-revision rates in patients undergoing revision total knee arthroplasty (rTKA) with cones/sleeves + stems to those with just stems.	total 2,882 = 2,264 Stem only + 618 cone/sleeve	Steam only vs Steam + cone/sleeve	Steam only vs Steam + cone/sleeve	Repeat revision for aseptic indications and specifically repeat revision due to aseptic loosening. Repeat revisions were defined as any operation following the index procedure in which a component was replaced.
79	To describe and emulate a target trial evaluating the comparative effectiveness of decompression with and without fusion surgery in Lumbar degenerative spondylolisthesis (DS) patients at 3-year follow-up.	total 215 = 153 Decompression alone + 62 Decompression plus fusion	Decompression alone vs Decompression plus fusion	Decompression alone vs Decompression plus fusion	The primary outcome was change in health-related quality of life at 3-year follow-up, measured by the EuroQol Health-Related Quality of Life 5-Dimension 3-Level questionnaire (EQ-5D-3L)<br>Secondary outcomes at 3-year follow-up included:<br>Change in Numeric Rating Scale (NRS) for back or leg pain intensity. NRS scores range from 0 to 10, with 10 indicating worst pain imaginable.<br>Change in Spinal Stenosis Measure (SSM) satisfaction subscale scores. SSM satisfaction subscale scores range from 1 to 4, with 4 indicating very dissatisfied.<br>Physical therapy utilization—a binary (yes/no) outcome of physical therapy utilization.<br>Oral analgesic use—a binary (yes/no) outcome of oral analgesic intake.
80	To compare the minimum 2-year patient-reported outcomes (PROs) and risk of revision or conversion to arthroplasty between primary labral reconstruction and primary labral repair.	total 853 = 129 labral reconstruction + 724 labral repair	labral repair vs labral reconstruction	labral repair vs labral reconstruction	Hip Outcome Score Activities of Daily Living subscale (HOS-ADL), Hip Outcome Score Sport subscale (HOS-Sport), modified Harris hip score (mHHS), Western Ontario and McMaster Universities Osteoarthritis Index total score (WOMAC), Tegner activity scale, 12-Item Short Form Health Survey Physical Component Summary score (SF-12 PCS), and 12-Item Short Form Health Survey Mental Component Summary score (SF-12 MCS)
81	To compare the early postoperative mortality and morbidity in older patients with a fracture of the femoral neck, between those who underwent total hip arthroplasty (THA) and those who underwent hemiarthroplasty.	total 165,123 = 6,501 THA + 158,622 Hemiarthroplasty"	THA vs Hemiarthroplast y	THA vs Hemiarthroplast y	Mortality and complications while in hospital<br><br>secondary outcomes were readmission and reoperation within one and two years after discharge, and the costs of hospitalization.
82	To compare outcomes between the Surgical Implant Generation Network (SIGN) standard nail and SIGN Fin Nails in adult femoral shaft fractures treated	total 2,912 = 1,932 Standard Nail + 965 Fin Nail	Standard Nail vs Fin Nail	Standard Nail vs Fin Nail	Achieving full painless weight bearing<br><br>secondary outcomes assessed were radiographic healing

	with a retrograde approach.				and infection.
83	To compare surgical outcomes and radiographic evaluations for single-level decompression cases at L1–L2 or L2–L3 between MEL and open laminectomy.	total 80 = 51 MEL + 29 Open	microendoscopic laminectomy (MEL) vs open laminectomy (open)	microendoscopic laminectomy (MEL) vs open laminectomy (open)	EuroQol 5-Dimension (EQ-5D) questionnairw and Oswestry Disability Index (ODI). Additionally, the Numeric Rating Scale (NRS)17 was utilized to assess pain in the back and leg. Preoperative and postoperative scores were compared between the 2 groups, and changes (Δ) from preoperative to postoperative assessments were also analyzed.<br><br>Imaging findings constituted secondary outcome measures.
84	To compare change at 1-year of walking ability, health-related quality of life, leg and back pain in patients with symptomatic Lumbar spinal stenosis (LSS) referred to a spine surgery clinic who opted for surgery and those who did not.	total 298 = 149 + 149	surgical decompression (various types)	non-surgical management	Walking improvement reported by the patient in item 4 of the Oswestry Disability Index (ODI)<br><br>Secondary outcomes were health-related quality of life measured by EQ-5D-3L (EuroQol Group, 1990) and the global assessment (GA) transition question
85	To answer a national research priority by comparing the risk-benefit and costs associated with reverse total shoulder replacement (RTSR) and anatomical total shoulder replacement (TSR) in patients having elective primary shoulder replacement for osteoarthritis.	total 12,986 = 9,410 TSR + 3,576 RTSR	RTSR s TSR	RTSR s TSR	Revision surgery.<br><br>Secondary outcome measures included serious adverse events within 90 days, reoperations within 12 months, prolonged hospital stay (more than three nights), change in Oxford Shoulder Score (preoperative to six month postoperative), and lifetime costs to the healthcare service

Table J

Details/results extracted from source of evidence charting table

ID	Causal Inference Methods Used	Use of Target Trial	Use of Causal Graphs	Centre Type	Rationale for covariate selection	Protocol	Clinical Gait Analysis
1	1-1 caliper 0.25 PSM	No	No	Unicentric	Literature	Not publicly available	Yes
2	Bayesian Additive Regression Trees (BART)	No	Yes	Unicentric	DAG	Not publicly available	Yes
3	IV	No	No	Multicentric	Literature	Not publicly available	No
4	IPTW	No	No	Multicentric	Not mentioned	Not publicly available	No
5	IPTW	No	No	Multicentric	Not mentioned	Not publicly available	No
6	IV	No	No	Multicentric	Literature	Not publicly available	No
7	IV	No	No	Multicentric	Not mentioned	Not publicly available	No
8	IPTW	No	No	Multicentric	Literature	Not publicly available	No
9	IPTW	No	No	Multicentric	Literature	Not publicly available	No
10	stabilized IPTW	No	No	Multicentric	Not mentioned	Not publicly available	No
11	IV	No	No	Multicentric	Literature	Not publicly available	No
12	IPTW	No	No	Unicentric	Not mentioned	Not publicly available	No
13	IPTW	No	No	Multicentric	Literature	Not publicly available	No
14	IPTW	No	No	Unicentric	Not mentioned	Not publicly available	No
15	IPTW	No	No	Unicentric	Not mentioned	Not publicly available	No
16	IPTW	No	No	Multicentric	Not mentioned	Not publicly available	No
17	IPTW	No	No	Unicentric	Not mentioned	Not publicly available	No
18	IPTW	No	No	Unicentric	Not mentioned	Not publicly available	No
19	stabilized IPTW	No	No	Multicentric	Not mentioned	Not publicly available	No
20	IPTW	No	No	Unicentric	Literature	Not publicly available	No
21	potential outcomes simulation (counterfactuals)	No	No	Multicentric	Not mentioned	Not publicly available	No
22	IPTW	No	No	Multicentric	Not mentioned	Not publicly available	No
23	PS stratification, IV	No	No	Multicentric	Not mentioned	Not publicly available	No
24	IPTW	No	No	Multicentric	Not mentioned	Not	No

						publicly available	
25	IV	No	No	Multicentric	Literature	Not publicly available	No
26	1-1 PSM + IPTW	No	No	Multicentric	Not mentioned	Not publicly available	No
27	IPTW	No	No	Unicentric	Not mentioned	Not publicly available	No
28	IPTW	No	No	Unicentric	Literature	Not publicly available	No
29	IV	No	No	Multicentric	Not mentioned	Not publicly available	No
30	1-5 caliper 0.2 PSM, stabilized IPTW, PS stratification, PS adjustement, IV	Yes	No	Multicentric	Not mentioned	Not publicly available	No
31	IPTW	No	No	Multicentric	Not mentioned	Not publicly available	No
32	PS stratification	No	No	Multicentric	Not mentioned	Not publicly available	No
33	PS stratification	No	No	Multicentric	Not mentioned	Not publicly available	No
34	IPTW	No	No	Unicentric	Not mentioned	Not publicly available	No
35	stabilized IPTW	No	Yes	Multicentric	DAG	Not publicly available	No
36	1-1 caliper 0.1 PSM	No	No	Unicentric	Not mentioned	Not publicly available	No
37	1-1 caliper 0.2 PSM	No	No	Unicentric	Literature	Not publicly available	No
38	1-2 PSM	No	No	Unicentric	Literature	Publicly available	No
39	1-1 caliper 0.2 PSM	No	No	Multicentric	Not mentioned	Not publicly available	No
40	1-1 PSM + IPTW	No	No	Multicentric	Not mentioned	Not publicly available	No
41	1-1 PSM	No	Yes	Multicentric	DAG	Not publicly available	No
42	1-1 PSM, IPTW	No	No	Unicentric	Not mentioned	Not publicly available	No
43	1-1 PSM	No	No	Multicentric	Not mentioned	Not publicly available	No
44	IPTW	No	No	Unicentric	Not mentioned	Not publicly available	No
45	PS adjustement	No	No	Multicentric	Not mentioned	Not publicly available	No
46	2-1 caliper 0.2 PSM	No	No	Multicentric	Not mentioned	Not publicly available	No
47	1-1 caliper 0.2 PSM	No	No	Multicentric	Not mentioned	Not publicly available	No
48	1-1 PSM	No	No	Unicentric	Literature	Not publicly	No

						available	
49	IPTW	No	No	Multicentric	Not mentioned	Not publicly available	No
50	IPTW	No	No	Unicentric	Not mentioned	Not publicly available	No
51	IPTW (Matching weights), PSM	No	No	Multicentric	Not mentioned	Not publicly available	No
52	IPTW (overlap weighting)	No	No	Multicentric	Clinical expertise	Not publicly available	No
53	1-1 caliper 0.15 PSM	No	No	Multicentric	Not mentioned	Publicly available	No
54	1-1 PSM	No	No	Unicentric	Not mentioned	Not publicly available	No
55	1-1 caliper 0.3 PSM	No	No	Unicentric	Not mentioned	Not publicly available	No
56	1-1 PSM, CEM	No	No	Unicentric	Literature	Not publicly available	No
57	1-1 PSM	No	No	Multicentric	Not mentioned	Not publicly available	No
58	4-1 PSM, IPTW	No	No	Multicentric	Not mentioned	Not publicly available	No
59	1-1 PSM, IPTW, PS adjustement	No	Yes	Multicentric	DAG	Not publicly available	No
60	2-1 PSM	No	No	Unicentric	Not mentioned	Not publicly available	No
61	1-1 caliper 0.2 PSM, stabilized IPTW	No	No	Multicentric	Not mentioned	Not publicly available	No
62	stabilized IPTW	No	No	Multicentric	Not mentioned	Not publicly available	No
63	1-1 PSM	No	Yes	Multicentric	DAG	Not publicly available	No
64	1-10 PSM	Yes	No	Multicentric	Not mentioned	Publicly available	No
65	IPTW	No	No	Unicentric	Multivariate analysis	Not publicly available	No
66	1-1 PSM	No	No	Multicentric	Not mentioned	Not publicly available	No
67	IPTW	No	No	Multicentric	Not mentioned	Not publicly available	No
68	1-1 PSM	No	No	Multicentric	Not mentioned	Not publicly available	No
69	TMLE	No	No	Multicentric	Not mentioned	Not publicly available	No
70	IPTW	No	No	Unicentric	Not mentioned	Not publicly available	No
71	1-4 PSM, IPTW	No	No	Unicentric	Multivariate analysis	Not publicly available	No
72	CMA	No	Yes	Multicentric	DAG	Not publicly available	No
73	1-1 caliper 0.1 PSM	No	No	Unicentric	Not mentioned	Not publicly available	No

74	IPTW	No	No	Multicentric	Not mentioned	Not publicly available	No
75	IPTW	No	No	Multicentric	Not mentioned	Not publicly available	No
76		Yes	No	Multicentric	Not mentioned	Publicly available	No
77	IPTW	No	No	Multicentric	Not mentioned	Not publicly available	No
78	IPTW	No	No	Multicentric	Not mentioned	Not publicly available	No
79	IPTW	Yes	No	Multicentric	Clinical expertise, Literature	Publicly available	No
80	IPTW	No	No	Unicentric	Not mentioned	Not publicly available	No
81	IV, IPTW (overlap weighting)	No	No	Multicentric	Literature	Not publicly available	No
82	IPTW	No	Yes	Multicentric	DAG	Not publicly available	No
83	IPTW	No	No	Multicentric	Literature	Not publicly available	No
84	1-1 caliper 0.2 PSM	No	No	Multicentric	Not mentioned	Not publicly available	No
85	1-1 caliper 0.2 PSM, IPTW	No	No	Multicentric	Not mentioned	Not publicly available	No

Table K
Covariates used in the propensity score model

ID	Patient risk factors	Disease/injury factors	Treatment/procedure factors	Surgeon factors	Hospital/centre factors	Timing factors
1	Yes	Yes	No	No	No	No
4	Yes	No	No	No	No	No
5	Yes	Yes	No	No	No	No
8	Yes	No	Yes	Yes	Yes	No
9	Yes	Yes	Yes	No	No	No
10	Yes	No	Yes	No	No	Yes
12	Yes	No	No	No	No	No
13	Yes	Yes	Yes	No	No	No
14	Yes	Yes	Yes	No	No	Yes
15	Yes	No	No	No	No	Yes
16	Yes	Yes	No	No	No	No
17	Yes	No	No	No	No	No
18	Yes	No	Yes	No	No	No
19	Yes	Yes	No	No	Yes	Yes
20	Yes	No	No	No	No	Yes
22	Yes	Yes	No	No	No	No
23	Yes	No	No	No	Yes	No
24	Yes	Yes	No	No	No	No
26	Yes	No	No	No	Yes	No
27	Yes	No	Yes	No	No	No
28	Yes	No	No	No	No	Yes
30	Yes	No	No	No	Yes	No
31	Yes	Yes	No	No	No	No
32	Yes	No	Yes	Yes	Yes	No
33	Yes	No	No	Yes	Yes	No
34	Yes	No	Yes	No	No	No
35	Yes	No	Yes	No	Yes	Yes
36	Yes	Yes	No	No	No	No
37	Yes	Yes	No	No	No	No
38	Yes	No	No	No	No	No
39	Yes	No	No	No	Yes	No
40	Yes	No	No	No	Yes	No
41	Yes	Yes	No	No	No	No
42	Yes	No	No	No	No	No
43	Yes	Yes	Yes	No	Yes	No
44	Yes	Yes	No	No	No	No
45	Yes	Yes	No	No	No	Yes
46	Yes	No	No	No	Yes	No
47	Yes	Yes	No	No	No	No
48	Yes	No	Yes	No	No	No
49	Yes	Yes	Yes	No	No	Yes
50	Yes	Yes	No	No	No	Yes
51	Yes	No	No	No	No	No
52	Yes	No	No	No	Yes	No
53	Yes	No	Yes	No	No	No
54	Yes	Yes	No	No	No	No
55	Yes	Yes	No	No	No	No
56	No	Yes	No	No	No	No
57	Yes	No	No	No	Yes	No
58	Yes	Yes	Yes	No	No	No
59	Yes	No	Yes	No	Yes	No
60	Yes	No	No	No	No	Yes
61	Yes	No	No	No	Yes	No
62	Yes	No	No	No	Yes	Yes
63	Yes	Yes	No	No	No	No
64	Yes	No	Yes	No	No	No
65	Yes	Yes	No	No	No	No
66	Yes	No	No	No	Yes	No
67	Yes	Yes	Yes	Yes	Yes	Yes
68	Yes	No	No	No	No	No
70	Yes	No	No	No	No	No
71	Yes	Yes	Yes	No	No	No
73	Yes	No	No	No	No	No
74	Yes	No	No	No	No	No
75	Yes	Yes	No	No	No	Yes
77	Yes	Yes	Yes	No	No	Yes
78	Yes	No	No	No	No	No
79	Yes	Yes	Yes	No	No	Yes

80	Yes	Yes	No	No	No	No
82	Yes	Yes	Yes	No	No	Yes
83	Yes	No	Yes	No	No	No
84	Yes	Yes	No	No	No	No
85	Yes	Yes	No	No	No	No

Note. Only methods using a propensity score are shown: PSM, IPTW, PS stratification, and PS adjustment. Columns represent covariate categories: patient risk factors (demographics, comorbidities, baseline function, treatment history); disease/injury factors (diagnosis severity, fracture classification, revision status); treatment/procedure factors (surgical approach, implant, concomitant procedures); surgeon factors (surgeon experience, surgeon volume); hospital/centre factors (region, centre volume); and timing factors (year, time to surgery, urgent vs elective).

Table L
Assessment of Risk of Bias

ID	Bias due to confounding	Bias in selection of participants into the study	Bias in classification of interventions	Bias due to deviations from intended intervention	Bias due to missing data	Bias in measurement of outcomes	Bias in selection of the reported result
1	(i) Confounding expected, all known important confounding domains appropriately measured and controlled for; and (ii) Reliability and validity of measurement of important domains were sufficient, such that we do not expect serious residual confounding.	Not all patients who are treated are seen both pre- and posttreatment	Intervention status is well defined; and Intervention definition is based solely on information collected at the time of intervention.	Any deviations from intended intervention reflected usual practice	The analysis addressed missing data and is likely to have removed any risk of bias.	(i) The methods of outcome assessment were comparable across intervention groups; and (ii) The outcome measure was unlikely to be influenced by knowledge of the intervention received by study participants (i.e. is objective) or the outcome assessors were unaware of the intervention received by study participants; and (iii) Any error in measuring the outcome is unrelated to intervention status.	Defined a priori in RCT
2	(i) Confounding expected, all known important confounding domains appropriately measured and controlled for; and (ii) Reliability and validity of measurement of important domains were sufficient, such that we do not expect serious residual confounding.	Prospectively recruited intervention group; but They did not recruit a prospective control group. Rather, they identified participants who were evaluated at multiple CGAs without any intervening surgical procedures; and For nine participants they used the six month follow-up visit due to pandemic and other scheduling related disruptions.	Intervention status is well defined; and Intervention definition is based solely on information collected at the time of intervention.	Any deviations from intended intervention reflected usual practice	Data were reasonably complete and Baseline impairments were not available for all participants. Missing data were imputed using multivariate imputation by chained equations (MICE).	(i) The methods of outcome assessment were comparable across intervention groups; and (ii) The outcome measure was unlikely to be influenced by knowledge of the intervention received by study participants (i.e. is objective) or the outcome assessors were unaware of the intervention received by study participants; and (iii) Any error in measuring the outcome is unrelated to intervention status.	The outcome measurements and analyses are consistent with an a priori plan; and There is no indication of selection of the reported analysis from among multiple analyses; and There is no indication of selection of the cohort or subgroups for analysis and reporting on the basis of the results.
3	At least one	(i) All	Intervention	Any deviations	Data were	(i) The methods	(i) The outcome

	important domain was not appropriately measured, e.g.: surgical approach, implant details, body mass index	participants who would have been eligible for the target trial were included in the study; and (ii) For each participant, start of follow up and start of intervention coincided.	status is well defined; and Intervention definition is based solely on information collected at the time of intervention.	from intended intervention reflected usual practice	reasonably complete	of outcome assessment were comparable across intervention groups; and (ii) The outcome measure was unlikely to be influenced by knowledge of the intervention received by study participants (i.e. is objective) or the outcome assessors were unaware of the intervention received by study participants;and (iii) Any error in measuring the outcome is unrelated to intervention status.	measurements and analyses are consistent with an a priori plan; or are clearly defined and both internally and externally consistent; and (ii) There is no indication of selection of the reported analysis from among multiple analyses; and (iii) There is no indication of selection of the cohort or subgroups for analysis and reporting on the basis of the results.
4	At least one known important domain was not appropriately measured: Data regarding American Society of Anesthesiologists scores, disease severity, weight, and occupation were missing from the South Korean NHI claims database and are important criteria in deciding, at a certain patient age, which of the 2 surgeries to recommend.	(i) All participants who would have been eligible for the target trial were included in the study; and (ii) For each participant, start of follow up and start of intervention coincided.	Intervention status is well defined; and Intervention definition is based solely on information collected at the time of intervention.	Any deviations from intended intervention reflected usual practice	Data were reasonably complete	(i) The methods of outcome assessment were comparable across intervention groups; and (ii) The outcome measure was unlikely to be influenced by knowledge of the intervention received by study participants (i.e. is objective) or the outcome assessors were unaware of the intervention received by study participants;and (iii) Any error in measuring the outcome is unrelated to intervention status.	(i) The outcome measurements and analyses are consistent with an a priori plan; or are clearly defined and both internally and externally consistent; and (ii) There is no indication of selection of the reported analysis from among multiple analyses; and (iii) There is no indication of selection of the cohort or subgroups for analysis and reporting on the basis of the results.
5	(i) Confounding expected, all known important confounding domains appropriately measured and controlled for; and (ii) Reliability and validity of	(i) Selection into the study may have been related to intervention and outcome; and The authors used appropriate methods to adjust for the	Intervention status is not well defined; PT (amoutn, duration, type of exercises etc.) is not uniformly defined, only start date of PT is recorded	Any deviations from intended intervention reflected usual practice	Excluded missing data	(i) The methods of outcome assessment were comparable across intervention groups; and (ii) The outcome measure was unlikely to be	(i) The outcome measurements and analyses are consistent with an a priori plan; or are clearly defined and both internally and externally consistent; and (ii) There is no

	measurement of important domains were sufficient, such that we do not expect serious residual confounding.	selection bias;				influenced by knowledge of the intervention received by study participants (i.e. is objective) or the outcome assessors were unaware of the intervention received by study participants;and (iii) Any error in measuring the outcome is unrelated to intervention status.	indication of selection of the reported analysis from among multiple analyses; and (iii) There is no indication of selection of the cohort or subgroups for analysis and reporting on the basis of the results.
6	(i) Confounding expected, all known important confounding domains appropriately measured and controlled for; and (ii) Reliability and validity of measurement of important domains were sufficient, such that we do not expect serious residual confounding.	(i) All participants who would have been eligible for the target trial were included in the study; and (ii) For each participant, start of follow up and start of intervention coincided.	Intervention status is well defined; and Intervention definition is based solely on information collected at the time of intervention.	Any deviations from intended intervention reflected usual practice	Proportions of and reasons for missing participants were similar across intervention groups	(i) The methods of outcome assessment were comparable across intervention groups; and (ii) The outcome measure was unlikely to be influenced by knowledge of the intervention received by study participants (i.e. is objective) or the outcome assessors were unaware of the intervention received by study participants;and (iii) Any error in measuring the outcome is unrelated to intervention status.	(i) The outcome measurements and analyses are consistent with an a priori plan; or are clearly defined and both internally and externally consistent; and (ii) There is no indication of selection of the reported analysis from among multiple analyses; and (iii) There is no indication of selection of the cohort or subgroups for analysis and reporting on the basis of the results.
7	At least one known important domain was not appropriately measured; and instrumental variable analysis was applied to address this bias	(i) All participants who would have been eligible for the target trial were included in the study; and (ii) For each participant, start of follow up and start of intervention coincided.	Intervention status is well defined; and Intervention definition is based solely on information collected at the time of intervention.	Any deviations from intended intervention reflected usual practice	Data were reasonably complete	The outcome measure was subjective (self reported); and The outcome was assessed by assessors aware of the intervention received by study participants;	(i) The outcome measurements and analyses are consistent with an a priori plan; or are clearly defined and both internally and externally consistent; and (ii) There is no indication of selection of the reported analysis from among multiple analyses; and (iii) There is no indication of selection of the cohort or subgroups for

							analysis and reporting on the basis of the results.
8	(i) Confounding expected, all known important confounding domains appropriately measured and controlled for; and (ii) Reliability and validity of measurement of important domains were sufficient, such that we do not expect serious residual confounding.	(i) All participants who would have been eligible for the target trial were included in the study; and (ii) For each participant, start of follow up and start of intervention coincided.	Intervention status is well defined; and Intervention definition is based solely on information collected at the time of intervention.	Any deviations from intended intervention reflected usual practice	Data were reasonably complete and Missing data were handled using multiple imputation.	(i) The methods of outcome assessment were comparable across intervention groups; and (ii) The outcome measure was unlikely to be influenced by knowledge of the intervention received by study participants (i.e. is objective) or the outcome assessors were unaware of the intervention received by study participants;and (iii) Any error in measuring the outcome is unrelated to intervention status.	(i) The outcome measurements and analyses are consistent with an a priori plan; or are clearly defined and both internally and externally consistent; and (ii) There is no indication of selection of the reported analysis from among multiple analyses; and (iii) There is no indication of selection of the cohort or subgroups for analysis and reporting on the basis of the results.
9	(i) Confounding expected, all known important confounding domains appropriately measured and controlled for; and (ii) Reliability and validity of measurement of important domains were sufficient, such that we do not expect serious residual confounding.	(i) All participants who would have been eligible for the target trial were included in the study; and (ii) For each participant, start of follow up and start of intervention coincided.	Intervention status is well defined; and Intervention definition is based solely on information collected at the time of intervention.	Any deviations from intended intervention reflected usual practice	Data were reasonably complete	(i) The methods of outcome assessment were comparable across intervention groups; and (ii) The outcome measure was unlikely to be influenced by knowledge of the intervention received by study participants (i.e. is objective) or the outcome assessors were unaware of the intervention received by study participants;and (iii) Any error in measuring the outcome is unrelated to intervention status.	(i) The outcome measurements and analyses are consistent with an a priori plan; or are clearly defined and both internally and externally consistent; and (ii) There is no indication of selection of the reported analysis from among multiple analyses; and (iii) There is no indication of selection of the cohort or subgroups for analysis and reporting on the basis of the results.
10	At least one important domain was not appropriately measured (severity of deformity)	All participants who would have been eligible for the target trial were included in the study;	Intervention status is well defined; and Intervention definition is based solely on	Any deviations from intended intervention reflected usual practice	Data were reasonably complete	(i) The methods of outcome assessment were comparable across intervention	(i) The outcome measurements and analyses are consistent with an a priori plan; or are clearly defined

		and<br>For each participant, start of follow up and start of intervention coincided.	information collected at the time of intervention.			groups; and<br>(ii) The outcome measure was unlikely to be influenced by knowledge of the intervention received by study participants (i.e. is objective) or the outcome assessors were unaware of the intervention received by study participants;and<br>(iii) Any error in measuring the outcome is unrelated to intervention status.	and both internally and externally consistent; and<br>(ii) There is no indication of selection of the reported analysis from among multiple analyses; and<br>(iii) There is no indication of selection of the cohort or subgroups for analysis and reporting on the basis of the results.
11	At least one known important domain was not appropriately measured: The AOANJRR commenced collection of patient American Society of Anesthesiologists (ASA) scores in 2012 and body mass index (BMI) and surgical approach in 2015; hence, these data are only available for a subset of patients and were not included in the final analysis.	All participants who would have been eligible for the target trial were included in the study;<br>and<br>For each participant, start of follow up and start of intervention coincided.	Intervention status is well defined;<br>and<br>Intervention definition is based solely on information collected at the time of intervention.	Any deviations from intended intervention reflected usual practice	Data were reasonably complete	(i) The methods of outcome assessment were comparable across intervention groups; and<br>(ii) The outcome measure was unlikely to be influenced by knowledge of the intervention received by study participants (i.e. is objective) or the outcome assessors were unaware of the intervention received by study participants;and<br>(iii) Any error in measuring the outcome is unrelated to intervention status.	(i) The outcome measurements and analyses are consistent with an a priori plan; or are clearly defined and both internally and externally consistent; and<br>(ii) There is no indication of selection of the reported analysis from among multiple analyses; and<br>(iii) There is no indication of selection of the cohort or subgroups for analysis and reporting on the basis of the results.
12	(i) Confounding expected, all known important confounding domains appropriately measured and controlled for; and<br>(ii) Reliability and validity of measurement of important domains were sufficient, such that we do not expect serious	(i) All participants who would have been eligible for the target trial were included in the study;<br>and<br>(ii) For each participant, start of follow up and start of intervention coincided.	Intervention status is well defined;<br>and<br>Intervention definition is based solely on information collected at the time of intervention.	Any deviations from intended intervention reflected usual practice	Proportions of and reasons for missing participants were similar across intervention groups	(i) The methods of outcome assessment were comparable across intervention groups; and<br>(ii) The outcome measure was unlikely to be influenced by knowledge of the intervention received by study participants (i.e.	(i) The outcome measurements and analyses are consistent with an a priori plan; or are clearly defined and both internally and externally consistent; and<br>(ii) There is no indication of selection of the reported analysis from among multiple analyses; and

	residual confounding.					is objective) or the outcome assessors were unaware of the intervention received by study participants;and (iii) Any error in measuring the outcome is unrelated to intervention status.<br><br>(some of the outcomes were self reported)	(iii) There is no indication of selection of the cohort or subgroups for analysis and reporting on the basis of the results.
13	(i) Confounding expected, all known important confounding domains appropriately measured and controlled for; and (ii) Reliability and validity of measurement of important domains were sufficient, such that we do not expect serious residual confounding.	(i) All participants who would have been eligible for the target trial were included in the study; and (ii) For each participant, start of follow up and start of intervention coincided.	Intervention status is well defined; and Intervention definition is based solely on information collected at the time of intervention.	Any deviations from intended intervention reflected usual practice	Proportions of and reasons for missing participants were similar across intervention groups<br><br>We restricted our analyses to patients with available preoperative data on the degree of contracture. There were no significant differences in the characteristics of patients with and without data available	(i) The methods of outcome assessment were comparable across intervention groups; and (ii) The outcome measure was unlikely to be influenced by knowledge of the intervention received by study participants (i.e. is objective) or the outcome assessors were unaware of the intervention received by study participants;and (iii) Any error in measuring the outcome is unrelated to intervention status.<br><br>(some of the outcomes were self reported)	(i) The outcome measurements and analyses are consistent with an a priori plan; or are clearly defined and both internally and externally consistent; and (ii) There is no indication of selection of the reported analysis from among multiple analyses; and (iii) There is no indication of selection of the cohort or subgroups for analysis and reporting on the basis of the results.
14	(i) Confounding expected, all known important confounding domains appropriately measured and controlled for; and (ii) Reliability and validity of measurement of important domains were sufficient, such that we do not expect serious residual confounding.	(i) All participants who would have been eligible for the target trial were included in the study; and (ii) For each participant, start of follow up and start of intervention coincided.	Intervention status is well defined; and Intervention definition is based solely on information collected at the time of intervention.	Effect of starting and adhering to intervention: (i) The important co-interventions were not balanced across intervention groups, or there were deviations from the intended interventions (in terms of implementation and/or adherence) that were likely to impact on the	Data were reasonably complete	(i) The methods of outcome assessment were comparable across intervention groups; and (ii) The outcome measure was unlikely to be influenced by knowledge of the intervention received by study participants (i.e. is objective) or the outcome assessors were	(i) The outcome measurements and analyses are consistent with an a priori plan; or are clearly defined and both internally and externally consistent; and (ii) There is no indication of selection of the reported analysis from among multiple analyses; and (iii) There is no indication of selection of the

				outcome (2 patients underwent PLIF at L3/L4 with BPL at L4/L5, and 13 patients underwent PLIF at L4/L5 with BPL at L3/L4); and (ii) The analysis was not appropriate to estimate the effect of starting and adhering to intervention, allowing for deviations (in terms of implementation, adherence and co-intervention) that were likely to impact on the outcome.		unaware of the intervention received by study participants;and (iii) Any error in measuring the outcome is unrelated to intervention status.	cohort or subgroups for analysis and reporting on the basis of the results.
15	(i) Confounding expected, all known important confounding domains appropriately measured and controlled for; and (ii) Reliability and validity of measurement of important domains were sufficient, such that we do not expect serious residual confounding.	(i) All participants who would have been eligible for the target trial were included in the study; and (ii) For each participant, start of follow up and start of intervention coincided.	Intervention status is well defined; and Intervention definition is based solely on information collected at the time of intervention.	Any deviations from intended intervention reflected usual practice	Data were reasonably complete	The methods of outcome assessment were not comparable across intervention groups: The follow-up duration (> 1 year shorter in the D group) may underestimate the rate of re-operation in D.	(i) The outcome measurements and analyses are consistent with an a priori plan; or are clearly defined and both internally and externally consistent; and (ii) There is no indication of selection of the reported analysis from among multiple analyses; and (iii) There is no indication of selection of the cohort or subgroups for analysis and reporting on the basis of the results.
16	(i) Confounding expected, all known important confounding domains appropriately measured and controlled for; and (ii) Reliability and validity of measurement of important domains were sufficient, such that we do not expect serious residual	(i) All participants who would have been eligible for the target trial were included in the study; and (ii) For each participant, start of follow up and start of intervention coincided.	Intervention status is well defined; and Intervention definition is based solely on information collected at the time of intervention.	Any deviations from intended intervention reflected usual practice	The analysis is unlikely to have removed the risk of bias arising from the missing data: missing data on number of fusion levels could have influenced our results.	(i) The methods of outcome assessment were comparable across intervention groups; and (ii) The outcome measure was unlikely to be influenced by knowledge of the intervention received by study participants (i.e. is objective) or	(i) The outcome measurements and analyses are consistent with an a priori plan; or are clearly defined and both internally and externally consistent; and (ii) There is no indication of selection of the reported analysis from among multiple analyses; and (iii) There is no

	confounding.					the outcome assessors were unaware of the intervention received by study participants;and (iii) Any error in measuring the outcome is unrelated to intervention status.	indication of selection of the cohort or subgroups for analysis and reporting on the basis of the results.
17	At least one known important domain was not appropriately controlled for: prospective studies that adjusted factors (e.g., period of surgery, implant, and surgeon) are desired.	(i) All participants who would have been eligible for the target trial were included in the study; and (ii) For each participant, start of follow up and start of intervention coincided.	Intervention status is well defined; and Intervention definition is based solely on information collected at the time of intervention.	Any deviations from intended intervention reflected usual practice	Data were reasonably complete	(i) The methods of outcome assessment were comparable across intervention groups; and (ii) The outcome measure was unlikely to be influenced by knowledge of the intervention received by study participants (i.e. is objective) or the outcome assessors were unaware of the intervention received by study participants;and (iii) Any error in measuring the outcome is unrelated to intervention status.	(i) The outcome measurements and analyses are consistent with an a priori plan; or are clearly defined and both internally and externally consistent; and (ii) There is no indication of selection of the reported analysis from among multiple analyses; and (iii) There is no indication of selection of the cohort or subgroups for analysis and reporting on the basis of the results.
18	(i) Confounding expected, all known important confounding domains appropriately measured and controlled for; and (ii) Reliability and validity of measurement of important domains were sufficient, such that we do not expect serious residual confounding.	(i) All participants who would have been eligible for the target trial were included in the study; and (ii) For each participant, start of follow up and start of intervention coincided.	Intervention status is well defined; and Intervention definition is based solely on information collected at the time of intervention.	Any deviations from intended intervention reflected usual practice	Data were reasonably complete	(i) The methods of outcome assessment were comparable across intervention groups; and (ii) The outcome measure was unlikely to be influenced by knowledge of the intervention received by study participants (i.e. is objective) or the outcome assessors were unaware of the intervention received by study participants;and (iii) Any error in measuring the outcome is unrelated to	(i) The outcome measurements and analyses are consistent with an a priori plan; or are clearly defined and both internally and externally consistent; and (ii) There is no indication of selection of the reported analysis from among multiple analyses; and (iii) There is no indication of selection of the cohort or subgroups for analysis and reporting on the basis of the results.

						intervention status.<br><br>(some of the outcomes were self reported)	
19	(i) Confounding expected, all known important confounding domains appropriately measured and controlled for; and<br>(ii) Reliability and validity of measurement of important domains were sufficient, such that we do not expect serious residual confounding.	(i) All participants who would have been eligible for the target trial were included in the study;<br>and<br>(ii) For each participant, start of follow up and start of intervention coincided.	Intervention status is well defined;<br>and<br>Intervention definition is based solely on information collected at the time of intervention.	Any deviations from intended intervention reflected usual practice	Data were reasonably complete<br>and<br>Missing data for the baseline characteristics were managed by mean and simple imputation for quantitative and qualitative variables, respectively. Missing KOOS data were managed by multiple imputations.	(i) The methods of outcome assessment were comparable across intervention groups; and<br>(ii) The outcome measure was unlikely to be influenced by knowledge of the intervention received by study participants (i.e. is objective) or the outcome assessors were unaware of the intervention received by study participants;and<br>(iii) Any error in measuring the outcome is unrelated to intervention status.<br><br>(some of the outcomes were self reported)	(i) The outcome measurements and analyses are consistent with an a priori plan; or are clearly defined and both internally and externally consistent; and<br>(ii) There is no indication of selection of the reported analysis from among multiple analyses; and<br>(iii) There is no indication of selection of the cohort or subgroups for analysis and reporting on the basis of the results.
20	At least one known important domain was not appropriately controlled for: One main limitation of our study is that in clinical practice, besides mBs, additional factors influence treatment decision whether decompressive surgery is indicated in acute MSCC or not. These factors may be measurable, such as burden of disease, poor performance status, severe comorbidities (e.g., renal/lung/cardiac function,	(i) All participants who would have been eligible for the target trial were included in the study;<br>and<br>(ii) For each participant, start of follow up and start of intervention coincided.	Intervention status is well defined;<br>and<br>Intervention definition is based solely on information collected at the time of intervention.	Any deviations from intended intervention reflected usual practice	Data were reasonably complete<br>and<br>imputed data: chained equations algorithm, 10 imputation datasets, separate imputation models for continu-ous, binary, and ordinal variables	(i) The methods of outcome assessment were comparable across intervention groups; and<br>(ii) The outcome measure was unlikely to be influenced by knowledge of the intervention received by study participants (i.e. is objective) or the outcome assessors were unaware of the intervention received by study participants;and<br>(iii) Any error in measuring the outcome is unrelated to intervention status.	(i) The outcome measurements and analyses are consistent with an a priori plan; or are clearly defined and both internally and externally consistent; and<br>(ii) There is no indication of selection of the reported analysis from among multiple analyses; and<br>(iii) There is no indication of selection of the cohort or subgroups for analysis and reporting on the basis of the results.

	dementia, anticoagulation therapy), and inoperability of a lesion, or unmeasurable, such as “clinical gestalt.”						
21	(i) Confounding expected, all known important confounding domains appropriately measured and controlled for; and<br>(ii) Reliability and validity of measurement of important domains were sufficient, such that we do not expect serious residual confounding.	(i) All participants who would have been eligible for the target trial were included in the study;<br>and<br>(ii) For each participant, start of follow up and start of intervention coincided.	Intervention status is well defined;<br>and<br>Intervention definition is based solely on information collected at the time of intervention.	Any deviations from intended intervention reflected usual practice	Data were reasonably complete<br>and<br>Missing data were handled using multiple imputation.	(i) The methods of outcome assessment were comparable across intervention groups; and<br>(ii) The outcome measure was unlikely to be influenced by knowledge of the intervention received by study participants (i.e. is objective) or the outcome assessors were unaware of the intervention received by study participants;and<br>(iii) Any error in measuring the outcome is unrelated to intervention status.<br><br>(some of the outcomes were self reported)	(i) The outcome measurements and analyses are consistent with an a priori plan; or are clearly defined and both internally and externally consistent; and<br>(ii) There is no indication of selection of the reported analysis from among multiple analyses; and<br>(iii) There is no indication of selection of the cohort or subgroups for analysis and reporting on the basis of the results.
22	At least one important domain was not appropriately measured (BMI, smoking habits, presence of psychosocial factors)	(i) All participants who would have been eligible for the target trial were included in the study;<br>and<br>(ii) For each participant, start of follow up and start of intervention coincided.	Intervention status is well defined;<br>and<br>Intervention definition is based solely on information collected at the time of intervention.	Any deviations from intended intervention reflected usual practice	Data were reasonably complete	(ii) The outcome measure was subjective (i.e. vulnerable to influence by knowledge of the intervention received by study participants);<br>and<br>The outcome was assessed by assessors aware of the intervention received by study	(i) The outcome measurements and analyses are consistent with an a priori plan; or are clearly defined and both internally and externally consistent; and<br>(ii) There is no indication of selection of the reported analysis from among multiple analyses; and<br>(iii) There is no indication of selection of the cohort or subgroups for analysis and reporting on the basis of the results.
23	(i) Confounding expected, all known	(i) All participants who would	Intervention status is well defined;	Any deviations from intended intervention	Data were reasonably complete	(i) The methods of outcome assessment	(i) The outcome measurements and analyses

	important confounding domains appropriately measured and controlled for; and (ii) Reliability and validity of measurement of important domains were sufficient, such that we do not expect serious residual confounding.	have been eligible for the target trial were included in the study; and (ii) For each participant, start of follow up and start of intervention coincided.	and Intervention definition is based solely on information collected at the time of intervention.	reflected usual practice		were comparable across intervention groups; and (ii) The outcome measure was unlikely to be influenced by knowledge of the intervention received by study participants (i.e. is objective) or the outcome assessors were unaware of the intervention received by study participants;and (iii) Any error in measuring the outcome is unrelated to intervention status.	are consistent with an a priori plan; or are clearly defined and both internally and externally consistent; and (ii) There is no indication of selection of the reported analysis from among multiple analyses; and (iii) There is no indication of selection of the cohort or subgroups for analysis and reporting on the basis of the results.
24	(i) Confounding expected, all known important confounding domains appropriately measured and controlled for; and (ii) Reliability and validity of measurement of important domains were sufficient, such that we do not expect serious residual confounding.	(i) All participants who would have been eligible for the target trial were included in the study; and (ii) For each participant, start of follow up and start of intervention coincided.	Intervention status is well defined; and Intervention definition is based solely on information collected at the time of intervention.	Any deviations from intended intervention reflected usual practice	Proportions of and reasons for missing participants were similar across intervention groups	(i) The methods of outcome assessment were comparable across intervention groups; and (ii) The outcome measure was unlikely to be influenced by knowledge of the intervention received by study participants (i.e. is objective) or the outcome assessors were unaware of the intervention received by study participants;and (iii) Any error in measuring the outcome is unrelated to intervention status.	(i) The outcome measurements and analyses are consistent with an a priori plan; or are clearly defined and both internally and externally consistent; and (ii) There is no indication of selection of the reported analysis from among multiple analyses; and (iii) There is no indication of selection of the cohort or subgroups for analysis and reporting on the basis of the results.
25	(i) Confounding expected, all known important confounding domains appropriately measured and controlled for; and (ii) Reliability and validity of measurement of important	(i) All participants who would have been eligible for the target trial were included in the study; and (ii) For each participant, start of follow up and start of intervention	Intervention status is well defined; and Intervention definition is based solely on information collected at the time of intervention.	Any deviations from intended intervention reflected usual practice	Data were reasonably complete	(i) The methods of outcome assessment were comparable across intervention groups; and (ii) The outcome measure was unlikely to be influenced by knowledge of	(i) The outcome measurements and analyses are consistent with an a priori plan; or are clearly defined and both internally and externally consistent; and (ii) There is no indication of selection of the

	domains were sufficient, such that we do not expect serious residual confounding.	coincided.				the intervention received by study participants (i.e. is objective) or the outcome assessors were unaware of the intervention received by study participants;and (iii) Any error in measuring the outcome is unrelated to intervention status.	reported analysis from among multiple analyses; and (iii) There is no indication of selection of the cohort or subgroups for analysis and reporting on the basis of the results.
26	At least one known important domain was not appropriately measured: No information about preoperative radiographs, preoperative activity level, or prior treatment was included in the database. Knee OA severity or patient activity level might affect patient selection and results.	(i) All participants who would have been eligible for the target trial were included in the study; and (ii) For each participant, start of follow up and start of intervention coincided.	Intervention status is well defined; and Intervention definition is based solely on information collected at the time of intervention.	Any deviations from intended intervention reflected usual practice	Data were reasonably complete	(i) The methods of outcome assessment were comparable across intervention groups; and (ii) The outcome measure was unlikely to be influenced by knowledge of the intervention received by study participants (i.e. is objective) or the outcome assessors were unaware of the intervention received by study participants;and (iii) Any error in measuring the outcome is unrelated to intervention status.	(i) The outcome measurements and analyses are consistent with an a priori plan; or are clearly defined and both internally and externally consistent; and (ii) There is no indication of selection of the reported analysis from among multiple analyses; and (iii) There is no indication of selection of the cohort or subgroups for analysis and reporting on the basis of the results.
27	(i) Confounding expected, all known important confounding domains appropriately measured and controlled for; and (ii) Reliability and validity of measurement of important domains were sufficient, such that we do not expect serious residual confounding.	(i) All participants who would have been eligible for the target trial were included in the study; and (ii) For each participant, start of follow up and start of intervention coincided.	Intervention status is well defined; and Intervention definition is based solely on information collected at the time of intervention.	Any deviations from intended intervention reflected usual practice	Data were reasonably complete	(i) The methods of outcome assessment were comparable across intervention groups; and (ii) The outcome measure was unlikely to be influenced by knowledge of the intervention received by study participants (i.e. is objective) or the outcome assessors were unaware of the intervention received by study	(i) The outcome measurements and analyses are consistent with an a priori plan; or are clearly defined and both internally and externally consistent; and (ii) There is no indication of selection of the reported analysis from among multiple analyses; and (iii) There is no indication of selection of the cohort or subgroups for analysis and reporting on the

						participants;and (iii) Any error in measuring the outcome is unrelated to intervention status.	basis of the results.
28	(i) Confounding expected, all known important confounding domains appropriately measured and controlled for; and (ii) Reliability and validity of measurement of important domains were sufficient, such that we do not expect serious residual confounding.	(i) All participants who would have been eligible for the target trial were included in the study; and (ii) For each participant, start of follow up and start of intervention coincided.	Intervention status is well defined; and Intervention definition is based solely on information collected at the time of intervention.	Any deviations from intended intervention reflected usual practice	Data were reasonably complete	(i) The methods of outcome assessment were comparable across intervention groups; and (ii) The outcome measure was unlikely to be influenced by knowledge of the intervention received by study participants (i.e. is objective) or the outcome assessors were unaware of the intervention received by study participants;and (iii) Any error in measuring the outcome is unrelated to intervention status.<br><br>(some of the outcomes were self reported)	(i) The outcome measurements and analyses are consistent with an a priori plan; or are clearly defined and both internally and externally consistent; and (ii) There is no indication of selection of the reported analysis from among multiple analyses; and (iii) There is no indication of selection of the cohort or subgroups for analysis and reporting on the basis of the results.
29	At least one important domain was not appropriately measured (e.g., patient comorbidities, time to surgery from hip fracture event)	(i) All participants who would have been eligible for the target trial were included in the study; and (ii) For each participant, start of follow up and start of intervention coincided.	Intervention status is well defined; and Intervention definition is based solely on information collected at the time of intervention.	Any deviations from intended intervention reflected usual practice	Data were reasonably complete	(i) The methods of outcome assessment were comparable across intervention groups; and (ii) The outcome measure was unlikely to be influenced by knowledge of the intervention received by study participants (i.e. is objective) or the outcome assessors were unaware of the intervention received by study participants;and (iii) Any error in measuring the outcome is unrelated to intervention status.	(i) The outcome measurements and analyses are consistent with an a priori plan; or are clearly defined and both internally and externally consistent; and (ii) There is no indication of selection of the reported analysis from among multiple analyses; and (iii) There is no indication of selection of the cohort or subgroups for analysis and reporting on the basis of the results.

30	(i) Confounding expected, all known important confounding domains appropriately measured and controlled for; and (ii) Reliability and validity of measurement of important domains were sufficient, such that we do not expect serious residual confounding.	(i) All participants who would have been eligible for the target trial were included in the study; and (ii) For each participant, start of follow up and start of intervention coincided.	Intervention status is well defined; and Intervention definition is based solely on information collected at the time of intervention.	Any deviations from intended intervention reflected usual practice	Proportions of and reasons for missing participants differ slightly across intervention groups: Missing Oxford Knee Scores may have led to information bias.	(ii) The outcome measure was subjective (i.e. vulnerable to influence by knowledge of the intervention received by study participants); and The outcome was assessed by assessors aware of the intervention received by study	(i) The outcome measurements and analyses are consistent with an a priori plan; or are clearly defined and both internally and externally consistent; and (ii) There is no indication of selection of the reported analysis from among multiple analyses; and (iii) There is no indication of selection of the cohort or subgroups for analysis and reporting on the basis of the results.
31	(i) Confounding expected, all known important confounding domains appropriately measured and controlled for; and (ii) Reliability and validity of measurement of important domains were sufficient, such that we do not expect serious residual confounding.	(i) All participants who would have been eligible for the target trial were included in the study; and (ii) For each participant, start of follow up and start of intervention coincided.	Intervention status is well defined; and Intervention definition is based solely on information collected at the time of intervention.	Any deviations from intended intervention reflected usual practice	Multiple imputation (MI) with predictive mean matching method was used to impute missing data among all 24 variables.	(ii) The outcome measure was subjective (i.e. vulnerable to influence by knowledge of the intervention received by study participants); and The outcome was assessed by assessors aware of the intervention received by study	(i) The outcome measurements and analyses are consistent with an a priori plan; or are clearly defined and both internally and externally consistent; and (ii) There is no indication of selection of the reported analysis from among multiple analyses; and (iii) There is no indication of selection of the cohort or subgroups for analysis and reporting on the basis of the results.
32	(i) Confounding expected, all known important confounding domains appropriately measured and controlled for; and (ii) Reliability and validity of measurement of important domains were sufficient, such that we do not expect serious residual confounding.	(i) All participants who would have been eligible for the target trial were included in the study; and (ii) For each participant, start of follow up and start of intervention coincided.	Intervention status is well defined; and Intervention definition is based solely on information collected at the time of intervention.	Any deviations from intended intervention reflected usual practice	Data were reasonably complete and Missing data were handled using multiple imputation. Ten imputed data sets were created and Rubin's rules for aggregating parameter estimates and variances were used.	(i) The methods of outcome assessment were comparable across intervention groups; and (ii) The outcome measure was unlikely to be influenced by knowledge of the intervention received by study participants (i.e. is objective) or the outcome assessors were	(i) The outcome measurements and analyses are consistent with an a priori plan; or are clearly defined and both internally and externally consistent; and (ii) There is no indication of selection of the reported analysis from among multiple analyses; and (iii) There is no indication of selection of the

						unaware of the intervention received by study participants;and (iii) Any error in measuring the outcome is unrelated to intervention status.	cohort or subgroups for analysis and reporting on the basis of the results.
33	(i) Confounding expected, all known important confounding domains appropriately measured and controlled for; and (ii) Reliability and validity of measurement of important domains were sufficient, such that we do not expect serious residual confounding.	(i) All participants who would have been eligible for the target trial were included in the study; and (ii) For each participant, start of follow up and start of intervention coincided.	Intervention status is well defined; and Intervention definition is based solely on information collected at the time of intervention.	Any deviations from intended intervention reflected usual practice	Data were reasonably complete and Missing data were handled using multiple imputation. Ten imputed data sets were created and Rubin's rules for aggregating parameter estimates and variances were used.	(i) The methods of outcome assessment were comparable across intervention groups; and (ii) The outcome measure was unlikely to be influenced by knowledge of the intervention received by study participants (i.e. is objective) or the outcome assessors were unaware of the intervention received by study participants;and (iii) Any error in measuring the outcome is unrelated to intervention status.	(i) The outcome measurements and analyses are consistent with an a priori plan; or are clearly defined and both internally and externally consistent; and (ii) There is no indication of selection of the reported analysis from among multiple analyses; and (iii) There is no indication of selection of the cohort or subgroups for analysis and reporting on the basis of the results.
34	At least one important domain was not appropriately measured (e.g., smoking, comorbidities)	(i) All participants who would have been eligible for the target trial were included in the study; and (ii) For each participant, start of follow up and start of intervention coincided.	Intervention status is well defined; and Intervention definition is based solely on information collected at the time of intervention.	Any deviations from intended intervention reflected usual practice	Data were reasonably complete and Multiple imputation was performed combining the modeled results of 50 imputations according to Rubin's rules to account for missing preoperative	(ii) The outcome measure was subjective (i.e. vulnerable to influence by knowledge of the intervention received by study participants); and The outcome was assessed by assessors aware of the intervention received by study	(i) The outcome measurements and analyses are consistent with an a priori plan; or are clearly defined and both internally and externally consistent; and (ii) There is no indication of selection of the reported analysis from among multiple analyses; and (iii) There is no indication of selection of the cohort or subgroups for analysis and reporting on the basis of the results.
35	(i) Confounding expected, all known important confounding	(i) All participants who would have been eligible for the	Intervention status is well defined; and Intervention	Any deviations from intended intervention reflected usual practice	Missing values in height were deemed too high for meaningful	(i) The methods of outcome assessment were comparable	(i) The outcome measurements and analyses are consistent with an a priori

	domains appropriately measured and controlled for; and (ii) Reliability and validity of measurement of important domains were sufficient, such that we do not expect serious residual confounding.	target trial were included in the study; and (ii) For each participant, start of follow up and start of intervention coincided.	definition is based solely on information collected at the time of intervention.		imputation and discarded. The remaining missing values were estimated by multiple imputation with chain equation (MICE), generating 5 datasets under the assumption of missing at random.	across intervention groups; and (ii) The outcome measure was unlikely to be influenced by knowledge of the intervention received by study participants (i.e. is objective) or the outcome assessors were unaware of the intervention received by study participants;and (iii) Any error in measuring the outcome is unrelated to intervention status.	plan; or are clearly defined and both internally and externally consistent; and (ii) There is no indication of selection of the reported analysis from among multiple analyses; and (iii) There is no indication of selection of the cohort or subgroups for analysis and reporting on the basis of the results.
36	At least one important domain was not appropriately measured (e.g. factors associated with fracture severity, such as the number of fragments per fracture or intra-articular lesions in the ORIF cohort)	(i) Selection into the study may have been related to intervention and outcome; and The authors used appropriate methods to adjust for the selection bias;	Intervention status is well defined; and Intervention definition is based solely on information collected at the time of intervention.	Any deviations from intended intervention reflected usual practice	Data were reasonably complete	(ii) The outcome measure was subjective (i.e. vulnerable to influence by knowledge of the intervention received by study participants); and The outcome was assessed by assessors aware of the intervention received by study	(i) The outcome measurements and analyses are consistent with an a priori plan; or are clearly defined and both internally and externally consistent; and (ii) There is no indication of selection of the reported analysis from among multiple analyses; and (iii) There is no indication of selection of the cohort or subgroups for analysis and reporting on the basis of the results.
37	(i) Confounding expected, all known important confounding domains appropriately measured and controlled for; and (ii) Reliability and validity of measurement of important domains were sufficient, such that we do not expect serious residual confounding.	(i) All participants who would have been eligible for the target trial were included in the study; and (ii) For each participant, start of follow up and start of intervention coincided.	Intervention status is well defined; and Intervention definition is based solely on information collected at the time of intervention.	Any deviations from intended intervention reflected usual practice	Data were reasonably complete	(ii) The outcome measure was subjective (i.e. vulnerable to influence by knowledge of the intervention received by study participants); and The outcome was assessed by assessors aware of the intervention received by study	(i) The outcome measurements and analyses are consistent with an a priori plan; or are clearly defined and both internally and externally consistent; and (ii) There is no indication of selection of the reported analysis from among multiple analyses; and (iii) There is no indication of selection of the

							cohort or subgroups for analysis and reporting on the basis of the results.
38	(i) Confounding expected, all known important confounding domains appropriately measured and controlled for; and (ii) Reliability and validity of measurement of important domains were sufficient, such that we do not expect serious residual confounding.	(i) Selection into the study may have been related to intervention and outcome; and The authors used appropriate methods to adjust for the selection bias;	Intervention status is well defined; and Intervention definition is based solely on information collected at the time of intervention.	Any deviations from intended intervention reflected usual practice	Data were reasonably complete	(i) The methods of outcome assessment were comparable across intervention groups; and (ii) The outcome measure was unlikely to be influenced by knowledge of the intervention received by study participants (i.e. is objective) or the outcome assessors were unaware of the intervention received by study participants;and (iii) Any error in measuring the outcome is unrelated to intervention status.	Defined a priori in study protocol
39	At least one important domain was not appropriately measured (e.g. meniscus damage and extrusion, cartilage status and bone marrow lesions)	(i) All participants who would have been eligible for the target trial were included in the study; and (ii) For each participant, start of follow up and start of intervention coincided.	Imprecision in defining arthroscopic meniscectomy and its timing may have influenced the results	Any deviations from intended intervention reflected usual practice	Data were reasonably complete: KOOS Function in Sport and Recreation had 25.8% missing values at baseline whereas other variables had less than 1.5% missing values and Multiple imputation procedures were used for missing values to increase precision and to avoid bias, generating ten independent imputed datasets	(i) The methods of outcome assessment were comparable across intervention groups; and (ii) The outcome measure was unlikely to be influenced by knowledge of the intervention received by study participants (i.e. is objective) or the outcome assessors were unaware of the intervention received by study participants;and (iii) Any error in measuring the outcome is unrelated to intervention status.	(i) The outcome measurements and analyses are consistent with an a priori plan; or are clearly defined and both internally and externally consistent; and (ii) There is no indication of selection of the reported analysis from among multiple analyses; and (iii) There is no indication of selection of the cohort or subgroups for analysis and reporting on the basis of the results.
40	At least one important domain was not appropriately measured (pre-	(i) All participants who would have been eligible for the	Intervention status is well defined; and Intervention	Any deviations from intended intervention reflected usual practice	Data were reasonably complete	(i) The methods of outcome assessment were comparable	(i) The outcome measurements and analyses are consistent with an a priori

	operative radiographs, pre-operative activity level, or surgeon volume)	target trial were included in the study; and (ii) For each participant, start of follow up and start of intervention coincided.	definition is based solely on information collected at the time of intervention.			across intervention groups; and (ii) The outcome measure was unlikely to be influenced by knowledge of the intervention received by study participants (i.e. is objective) or the outcome assessors were unaware of the intervention received by study participants;and (iii) Any error in measuring the outcome is unrelated to intervention status.	plan; or are clearly defined and both internally and externally consistent; and (ii) There is no indication of selection of the reported analysis from among multiple analyses; and (iii) There is no indication of selection of the cohort or subgroups for analysis and reporting on the basis of the results.
41	(i) Confounding expected, all known important confounding domains appropriately measured and controlled for; and (ii) Reliability and validity of measurement of important domains were sufficient, such that we do not expect serious residual confounding.	(i) All participants who would have been eligible for the target trial were included in the study; and (ii) For each participant, start of follow up and start of intervention coincided.	Intervention status is well defined; and Intervention definition is based solely on information collected at the time of intervention.	Any deviations from intended intervention reflected usual practice	Missing values were replaced with multiple imputation performed using chained equations as implemented in the R package MICE, generating 100 imputations. These imputations were analyzed one at a time, pooling the results using Rubin's rules. The following imputation models were used: predictive mean matching for numerical variables, logistic regression for dichotomous variables, and ordinal regression for ordinal variables. The proportion of missing values ranged from 0.1% for "number of degenerative levels" to 16.8% for "attitude towards returning to work after surgery."	(ii) The outcome measure was subjective (i.e. vulnerable to influence by knowledge of the intervention received by study participants); and The outcome was assessed by assessors aware of the intervention received by study	(i) The outcome measurements and analyses are consistent with an a priori plan; or are clearly defined and both internally and externally consistent; and (ii) There is no indication of selection of the reported analysis from among multiple analyses; and (iii) There is no indication of selection of the cohort or subgroups for analysis and reporting on the basis of the results.
42	(i) Confounding	(i) Selection	Intervention	Any deviations	We further	(ii) The	(i) The outcome

	expected, all known important confounding domains appropriately measured and controlled for; and (ii) Reliability and validity of measurement of important domains were sufficient, such that we do not expect serious residual confounding.	into the study may have been related to intervention and outcome; and The authors used appropriate methods to adjust for the selection bias;	status is well defined; and Intervention definition is based solely on information collected at the time of intervention.	from intended intervention reflected usual practice	limited the DSC cohort to patients with complete postoperative scores at a minimum of 40 days after and not later than 1-year after surgery. The ACLR cohort was further limited to patients over the age of 13 years with postoperative scores at a minimum of 133 days after and not later than 1-year after surgery	outcome measure was subjective (i.e. vulnerable to influence by knowledge of the intervention received by study participants); and The outcome was assessed by assessors aware of the intervention received by study	measurements and analyses are consistent with an a priori plan; or are clearly defined and both internally and externally consistent; and (ii) There is no indication of selection of the reported analysis from among multiple analyses; and (iii) There is no indication of selection of the cohort or subgroups for analysis and reporting on the basis of the results.
43	(i) Confounding expected, all known important confounding domains appropriately measured and controlled for; and (ii) Reliability and validity of measurement of important domains were sufficient, such that we do not expect serious residual confounding.	(i) All participants who would have been eligible for the target trial were included in the study; and (ii) For each participant, start of follow up and start of intervention coincided.	Intervention status is well defined; and Intervention definition is based solely on information collected at the time of intervention.	Any deviations from intended intervention reflected usual practice	Data were reasonably complete	(i) The methods of outcome assessment were comparable across intervention groups; and (ii) The outcome measure was unlikely to be influenced by knowledge of the intervention received by study participants (i.e. is objective) or the outcome assessors were unaware of the intervention received by study participants;and (iii) Any error in measuring the outcome is unrelated to intervention status.	(i) The outcome measurements and analyses are consistent with an a priori plan; or are clearly defined and both internally and externally consistent; and (ii) There is no indication of selection of the reported analysis from among multiple analyses; and (iii) There is no indication of selection of the cohort or subgroups for analysis and reporting on the basis of the results.
44	(i) Confounding expected, all known important confounding domains appropriately measured and controlled for; and (ii) Reliability and validity of measurement of important domains were sufficient, such that we do not	(i) Selection into the study may have been related to intervention and outcome; and The authors used appropriate methods to adjust for the selection bias; (Patients (or guardians) self-selected into ADM vs. non-	Intervention status is well defined; and Intervention definition is based solely on information collected at the time of intervention.	Any deviations from intended intervention reflected usual practice	Data were reasonably complete	(i) The methods of outcome assessment were comparable across intervention groups; and (ii) The outcome measure was unlikely to be influenced by knowledge of the intervention received by study	(i) The outcome measurements and analyses are consistent with an a priori plan; or are clearly defined and both internally and externally consistent; and (ii) There is no indication of selection of the reported analysis from among multiple

	expect serious residual confounding.	ADM after being informed about the material.)				participants (i.e. is objective) or the outcome assessors were unaware of the intervention received by study participants;and (iii) Any error in measuring the outcome is unrelated to intervention status.	analyses; and (iii) There is no indication of selection of the cohort or subgroups for analysis and reporting on the basis of the results.
45	At least one important domain was not appropriately measured (e.g. osteoporosis, which might have a significant influence on endplate injury and cage subsidence)	(i) All participants who would have been eligible for the target trial were included in the study; and (ii) For each participant, start of follow up and start of intervention coincided.	Intervention status is well defined; and Intervention definition is based solely on information collected at the time of intervention.	Any deviations from intended intervention reflected usual practice	Data were reasonably complete	(i) The methods of outcome assessment were comparable across intervention groups; and (ii) The outcome measure was unlikely to be influenced by knowledge of the intervention received by study participants (i.e. is objective) or the outcome assessors were unaware of the intervention received by study participants;and (iii) Any error in measuring the outcome is unrelated to intervention status.	(i) The outcome measurements and analyses are consistent with an a priori plan; or are clearly defined and both internally and externally consistent; and (ii) There is no indication of selection of the reported analysis from among multiple analyses; and (iii) There is no indication of selection of the cohort or subgroups for analysis and reporting on the basis of the results.
46	At least one important domain was not appropriately measured (cyst diagnosis, initial surgery)	(i) All participants who would have been eligible for the target trial were included in the study; and (ii) For each participant, start of follow up and start of intervention coincided.	Intervention status is well defined; and Intervention definition is based solely on information collected at the time of intervention.	Any deviations from intended intervention reflected usual practice	Data were reasonably complete	(i) The methods of outcome assessment were comparable across intervention groups; and (ii) The outcome measure was unlikely to be influenced by knowledge of the intervention received by study participants (i.e. is objective) or the outcome assessors were unaware of the intervention received by study participants;and (iii) Any error in measuring	(i) The outcome measurements and analyses are consistent with an a priori plan; or are clearly defined and both internally and externally consistent; and (ii) There is no indication of selection of the reported analysis from among multiple analyses; and (iii) There is no indication of selection of the cohort or subgroups for analysis and reporting on the basis of the results.

						the outcome is unrelated to intervention status.	
47	(i) Confounding expected, all known important confounding domains appropriately measured and controlled for; and (ii) Reliability and validity of measurement of important domains were sufficient, such that we do not expect serious residual confounding.	(i) All participants who would have been eligible for the target trial were included in the study; and (ii) For each participant, start of follow up and start of intervention coincided.	Intervention status is well defined; and Intervention definition is based solely on information collected at the time of intervention.	Any deviations from intended intervention reflected usual practice	Data were reasonably complete	(i) The methods of outcome assessment were comparable across intervention groups; and (ii) The outcome measure was unlikely to be influenced by knowledge of the intervention received by study participants (i.e. is objective) or the outcome assessors were unaware of the intervention received by study participants;and (iii) Any error in measuring the outcome is unrelated to intervention status.	(i) The outcome measurements and analyses are consistent with an a priori plan; or are clearly defined and both internally and externally consistent; and (ii) There is no indication of selection of the reported analysis from among multiple analyses; and (iii) There is no indication of selection of the cohort or subgroups for analysis and reporting on the basis of the results.
48	(i) Confounding expected, all known important confounding domains appropriately measured and controlled for; and (ii) Reliability and validity of measurement of important domains were sufficient, such that we do not expect serious residual confounding.	(i) All participants who would have been eligible for the target trial were included in the study; and (ii) For each participant, start of follow up and start of intervention coincided.	Intervention status is well defined; and Intervention definition is based solely on information collected at the time of intervention.	Any deviations from intended intervention reflected usual practice	Data were reasonably complete	(i) The methods of outcome assessment were comparable across intervention groups; and (ii) The outcome measure was unlikely to be influenced by knowledge of the intervention received by study participants (i.e. is objective) or the outcome assessors were unaware of the intervention received by study participants;and (iii) Any error in measuring the outcome is unrelated to intervention status.	(i) The outcome measurements and analyses are consistent with an a priori plan; or are clearly defined and both internally and externally consistent; and (ii) There is no indication of selection of the reported analysis from among multiple analyses; and (iii) There is no indication of selection of the cohort or subgroups for analysis and reporting on the basis of the results.
49	(i) Confounding expected, all known important confounding domains appropriately	(i) All participants who would have been eligible for the target trial were included in the	Intervention status is well defined; and Intervention definition is based solely on	Any deviations from intended intervention reflected usual practice	Multiple imputation was utilized to account for missing data for covariates. Multiple	(i) The methods of outcome assessment were comparable across intervention	(i) The outcome measurements and analyses are consistent with an a priori plan; or are clearly defined

	measured and controlled for; and (ii) Reliability and validity of measurement of important domains were sufficient, such that we do not expect serious residual confounding.	study; and (ii) For each participant, start of follow up and start of intervention coincided.	information collected at the time of intervention.		imputations were performed with use of chained equations to create 20 imputations, 5 iterations, of the analytic data set. The imputation model comprised the cement group, covariates, an event indicator, and the Nelson-Aalen estimator of the cumulative baseline hazard at the time of the event being modeled or censoring for each case for each distinct outcome. Weighted Cox models were fitted separately to each imputed data set and were aggregated across imputations using the Rubin rules.	groups; and (ii) The outcome measure was unlikely to be influenced by knowledge of the intervention received by study participants (i.e. is objective) or the outcome assessors were unaware of the intervention received by study participants;and (iii) Any error in measuring the outcome is unrelated to intervention status.	and both internally and externally consistent; and (ii) There is no indication of selection of the reported analysis from among multiple analyses; and (iii) There is no indication of selection of the cohort or subgroups for analysis and reporting on the basis of the results.
50	(i) Confounding expected, all known important confounding domains appropriately measured and controlled for; and (ii) Reliability and validity of measurement of important domains were sufficient, such that we do not expect serious residual confounding.	(i) Selection into the study was related (but not very strongly) to intervention and outcome; and This could not be adjusted for in analyses (variability of dilute PI irrigation use among surgeons in this study allows for there to have been potential bias in the selection of patients for PI irrigation use as well as in the evaluation of treatment effect.)	Intervention status is well defined; and Intervention definition is based solely on information collected at the time of intervention.	Any deviations from intended intervention reflected usual practice	Data were reasonably complete	(i) The methods of outcome assessment were comparable across intervention groups; and (ii) The outcome measure was unlikely to be influenced by knowledge of the intervention received by study participants (i.e. is objective) or the outcome assessors were unaware of the intervention received by study participants;and (iii) Any error in measuring the outcome is unrelated to intervention status.	(i) The outcome measurements and analyses are consistent with an a priori plan; or are clearly defined and both internally and externally consistent; and (ii) There is no indication of selection of the reported analysis from among multiple analyses; and (iii) There is no indication of selection of the cohort or subgroups for analysis and reporting on the basis of the results.
51	At least one important domain was not appropriately	(i) All participants who would have been	Intervention status is well defined; and	Any deviations from intended intervention reflected usual	Data were reasonably complete	(i) The methods of outcome assessment were	(i) The outcome measurements and analyses are consistent

	measured (e.g. type of fracture, severity of instability, degree of dislocation, or surgical techniques details)	eligible for the target trial were included in the study; and (ii) For each participant, start of follow up and start of intervention coincided.	Intervention definition is based solely on information collected at the time of intervention.	practice		comparable across intervention groups; and (ii) The outcome measure was unlikely to be influenced by knowledge of the intervention received by study participants (i.e. is objective) or the outcome assessors were unaware of the intervention received by study participants;and (iii) Any error in measuring the outcome is unrelated to intervention status.	with an a priori plan; or are clearly defined and both internally and externally consistent; and (ii) There is no indication of selection of the reported analysis from among multiple analyses; and (iii) There is no indication of selection of the cohort or subgroups for analysis and reporting on the basis of the results.
52	At least one important domain was not appropriately measured (e.g. results might have been affected by unmeasured confounders such as patient occupation, surgeon experience, or herniation type; radiologic information, such as the degree of degenerative change or presence of Modic change (pathological change in the end plate of the neighboring disc); and disc height)	(i) All participants who would have been eligible for the target trial were included in the study; and (ii) For each participant, start of follow up and start of intervention coincided.	Intervention status is well defined; and Intervention definition is based solely on information collected at the time of intervention.	Any deviations from intended intervention reflected usual practice	The analysis is unlikely to have removed the risk of bias arising from the missing data: We did not include BMI because these data were missing for a large proportion of patients.	(i) The methods of outcome assessment were comparable across intervention groups; and (ii) The outcome measure was unlikely to be influenced by knowledge of the intervention received by study participants (i.e. is objective) or the outcome assessors were unaware of the intervention received by study participants;and (iii) Any error in measuring the outcome is unrelated to intervention status.	(i) The outcome measurements and analyses are consistent with an a priori plan; or are clearly defined and both internally and externally consistent; and (ii) There is no indication of selection of the reported analysis from among multiple analyses; and (iii) There is no indication of selection of the cohort or subgroups for analysis and reporting on the basis of the results.
53	(i) Confounding expected, all known important confounding domains appropriately measured and controlled for; and (ii) Reliability and validity of measurement of important domains were	(i) All participants who would have been eligible for the target trial were included in the study; and (ii) For each participant, start of follow up and start of intervention coincided.	Intervention status is well defined; and Intervention definition is based solely on information collected at the time of intervention.	Any deviations from intended intervention reflected usual practice	Data were reasonably complete and Multiple imputation was applied (25 times) to impute missing values for baseline characteristics	(i) The methods of outcome assessment were comparable across intervention groups; and (ii) The outcome measure was unlikely to be influenced by knowledge of the intervention	Defined a priori in study protocol

	sufficient, such that we do not expect serious residual confounding.					received by study participants (i.e. is objective) or the outcome assessors were unaware of the intervention received by study participants;and (iii) Any error in measuring the outcome is unrelated to intervention status.	
54	(i) Confounding expected, all known important confounding domains appropriately measured and controlled for; and (ii) Reliability and validity of measurement of important domains were sufficient, such that we do not expect serious residual confounding.	(i) All participants who would have been eligible for the target trial were included in the study; and (ii) For each participant, start of follow up and start of intervention coincided.	Intervention status is well defined; and Intervention definition is based solely on information collected at the time of intervention.	Any deviations from intended intervention reflected usual practice	Data were reasonably complete and multiple missing imputation was performed with additive regression, bootstrapping and predictive mean matching	(i) The methods of outcome assessment were comparable across intervention groups; and (ii) The outcome measure was unlikely to be influenced by knowledge of the intervention received by study participants (i.e. is objective) or the outcome assessors were unaware of the intervention received by study participants;and (iii) Any error in measuring the outcome is unrelated to intervention status.	(i) The outcome measurements and analyses are consistent with an a priori plan; or are clearly defined and both internally and externally consistent; and (ii) There is no indication of selection of the reported analysis from among multiple analyses; and (iii) There is no indication of selection of the cohort or subgroups for analysis and reporting on the basis of the results.
55	At least one important domain was not appropriately measured (e.g. physical examination findings, biceps tendon pathology, rotator cuff retraction, and psychosocial factors)	(i) All participants who would have been eligible for the target trial were included in the study; and (ii) For each participant, start of follow up and start of intervention coincided.	Intervention status is well defined; and Intervention definition is based solely on information collected at the time of intervention.	Any deviations from intended intervention reflected usual practice	Data were reasonably complete	(ii) The outcome measure was subjective (i.e. vulnerable to influence by knowledge of the intervention received by study participants); and The outcome was assessed by assessors aware of the intervention received by study	(i) The outcome measurements and analyses are consistent with an a priori plan; or are clearly defined and both internally and externally consistent; and (ii) There is no indication of selection of the reported analysis from among multiple analyses; and (iii) There is no indication of selection of the cohort or subgroups for analysis and reporting on the basis of the

							results.
56	At least one important domain was not appropriately measured (e.g. comorbid depression or anxiety which have been associated with worse post-operative outcomes after undergoing spinal surgery)	(i) All participants who would have been eligible for the target trial were included in the study;<br>and<br>(ii) For each participant, start of follow up and start of intervention coincided.	Intervention status is well defined;<br>and<br>Intervention definition is based solely on information collected at the time of intervention.	Any deviations from intended intervention reflected usual practice	Data were reasonably complete	(ii) The outcome measure was subjective (i.e. vulnerable to influence by knowledge of the intervention received by study participants);<br>and<br>The outcome was assessed by assessors aware of the intervention received by study	(i) The outcome measurements and analyses are consistent with an a priori plan; or are clearly defined and both internally and externally consistent; and<br>(ii) There is no indication of selection of the reported analysis from among multiple analyses; and<br>(iii) There is no indication of selection of the cohort or subgroups for analysis and reporting on the basis of the results.
57	At least one important domain was not appropriately measured (e.g. radiological data, cervical alignment, and severity of heart failure)	(i) All participants who would have been eligible for the target trial were included in the study;<br>and<br>(ii) For each participant, start of follow up and start of intervention coincided.	Intervention status is well defined;<br>and<br>Intervention definition is based solely on information collected at the time of intervention.	Any deviations from intended intervention reflected usual practice	The analysis is unlikely to have removed the risk of bias arising from the missing data: Patients with missing data, emergency cases, and elective surgery were excluded from the study (12.4% of the data)	(i) The methods of outcome assessment were comparable across intervention groups; and<br>(ii) The outcome measure was unlikely to be influenced by knowledge of the intervention received by study participants (i.e. is objective) or the outcome assessors were unaware of the intervention received by study participants;and<br>(iii) Any error in measuring the outcome is unrelated to intervention status.	(i) The outcome measurements and analyses are consistent with an a priori plan; or are clearly defined and both internally and externally consistent; and<br>(ii) There is no indication of selection of the reported analysis from among multiple analyses; and<br>(iii) There is no indication of selection of the cohort or subgroups for analysis and reporting on the basis of the results.
58	At least one important domain was not appropriately measured (e.g. bone mineral density (BMD) and the osteoporosis treatment history were not evaluated in this study)	(i) All participants who would have been eligible for the target trial were included in the study;<br>and<br>(ii) For each participant, start of follow up and start of intervention coincided.	Intervention status is well defined;<br>and<br>Intervention definition is based solely on information collected at the time of intervention.	Any deviations from intended intervention reflected usual practice	Data were reasonably complete<br>and<br>multiple imputation was performed to evaluate sensitivity to missing data under the assumption that the data were missing at random	(i) The methods of outcome assessment were comparable across intervention groups; and<br>(ii) The outcome measure was unlikely to be influenced by knowledge of the intervention	(i) The outcome measurements and analyses are consistent with an a priori plan; or are clearly defined and both internally and externally consistent; and<br>(ii) There is no indication of selection of the reported

						received by study participants (i.e. is objective) or the outcome assessors were unaware of the intervention received by study participants;and (iii) Any error in measuring the outcome is unrelated to intervention status.	analysis from among multiple analyses; and (iii) There is no indication of selection of the cohort or subgroups for analysis and reporting on the basis of the results.
59	(i) Confounding expected, all known important confounding domains appropriately measured and controlled for; and (ii) Reliability and validity of measurement of important domains were sufficient, such that we do not expect serious residual confounding.	(i) All participants who would have been eligible for the target trial were included in the study; and (ii) For each participant, start of follow up and start of intervention coincided.	Intervention status is well defined; and Intervention definition is based solely on information collected at the time of intervention.	Any deviations from intended intervention reflected usual practice	Data were reasonably complete	(i) The methods of outcome assessment were comparable across intervention groups; and (ii) The outcome measure was unlikely to be influenced by knowledge of the intervention received by study participants (i.e. is objective) or the outcome assessors were unaware of the intervention received by study participants;and (iii) Any error in measuring the outcome is unrelated to intervention status.	(i) The outcome measurements and analyses are consistent with an a priori plan; or are clearly defined and both internally and externally consistent; and (ii) There is no indication of selection of the reported analysis from among multiple analyses; and (iii) There is no indication of selection of the cohort or subgroups for analysis and reporting on the basis of the results.
60	(i) Confounding expected, all known important confounding domains appropriately measured and controlled for; and (ii) Reliability and validity of measurement of important domains were sufficient, such that we do not expect serious residual confounding.	(i) All participants who would have been eligible for the target trial were included in the study; and (ii) For each participant, start of follow up and start of intervention coincided.	Intervention status is well defined; and Intervention definition is based solely on information collected at the time of intervention.	Effect of assignment to intervention: There were deviations from usual practice that were unbalanced between the intervention groups and likely to have affected the outcome. (hospital financial incentives could influence LOS differently)	The analysis is unlikely to have removed the risk of bias arising from the missing data: Preoperative KOOS Sports was not used as a covariate as several patients declined to answer it and a propensity matching algorithm requires that all covariates be complete for all patients.	(ii) The outcome measure was subjective (i.e. vulnerable to influence by knowledge of the intervention received by study participants); and The outcome was assessed by assessors aware of the intervention received by study participants;	(i) The outcome measurements and analyses are consistent with an a priori plan; or are clearly defined and both internally and externally consistent; and (ii) There is no indication of selection of the reported analysis from among multiple analyses; and (iii) There is no indication of selection of the cohort or subgroups for analysis and reporting on the basis of the

							results.
61	At least one important domain was not appropriately measured (e.g. important factors regarding dislocation, such as type of surgical approach and type and size of the implant, and angle of implant placement were not evaluated)	(i) All participants who would have been eligible for the target trial were included in the study;<br>and<br>(ii) For each participant, start of follow up and start of intervention coincided.	Intervention status is well defined;<br>and<br>Intervention definition is based solely on information collected at the time of intervention.	Any deviations from intended intervention reflected usual practice	Data were reasonably complete	(i) The methods of outcome assessment were comparable across intervention groups; and<br>(ii) The outcome measure was unlikely to be influenced by knowledge of the intervention received by study participants (i.e. is objective) or the outcome assessors were unaware of the intervention received by study participants;and<br>(iii) Any error in measuring the outcome is unrelated to intervention status.	(i) The outcome measurements and analyses are consistent with an a priori plan; or are clearly defined and both internally and externally consistent; and<br>(ii) There is no indication of selection of the reported analysis from among multiple analyses; and<br>(iii) There is no indication of selection of the cohort or subgroups for analysis and reporting on the basis of the results.
62	(i) Confounding expected, all known important confounding domains appropriately measured and controlled for; and<br>(ii) Reliability and validity of measurement of important domains were sufficient, such that we do not expect serious residual confounding.	(i) All participants who would have been eligible for the target trial were included in the study;<br>and<br>(ii) For each participant, start of follow up and start of intervention coincided.	Intervention status is well defined;<br>and<br>Intervention definition is based solely on information collected at the time of intervention.	Any deviations from intended intervention reflected usual practice	Data were reasonably complete	(i) The methods of outcome assessment were comparable across intervention groups; and<br>(ii) The outcome measure was unlikely to be influenced by knowledge of the intervention received by study participants (i.e. is objective) or the outcome assessors were unaware of the intervention received by study participants;and<br>(iii) Any error in measuring the outcome is unrelated to intervention status.	(i) The outcome measurements and analyses are consistent with an a priori plan; or are clearly defined and both internally and externally consistent; and<br>(ii) There is no indication of selection of the reported analysis from among multiple analyses; and<br>(iii) There is no indication of selection of the cohort or subgroups for analysis and reporting on the basis of the results.
63	(i) Confounding expected, all known important confounding domains appropriately measured and controlled for; and	(i) All participants who would have been eligible for the target trial were included in the study;<br>and<br>(ii) For each	Intervention status is well defined;<br>and<br>Intervention definition is based solely on information collected at the time of	Any deviations from intended intervention reflected usual practice	Data were reasonably complete<br>and<br>Missing values were replaced with multiple imputation using chained equations as	(ii) The outcome measure was subjective (i.e. vulnerable to influence by knowledge of the intervention received by study	(i) The outcome measurements and analyses are consistent with an a priori plan; or are clearly defined and both internally and externally

	(ii) Reliability and validity of measurement of important domains were sufficient, such that we do not expect serious residual confounding.	participant, start of follow up and start of intervention coincided.	intervention.		implemented in the R package MICE, generating 100 imputations.	participants); and<br>The outcome was assessed by assessors aware of the intervention received by study	consistent; and<br>(ii) There is no indication of selection of the reported analysis from among multiple analyses; and<br>(iii) There is no indication of selection of the cohort or subgroups for analysis and reporting on the basis of the results.
64	(i) Confounding expected, all known important confounding domains appropriately measured and controlled for; and<br>(ii) Reliability and validity of measurement of important domains were sufficient, such that we do not expect serious residual confounding.	(i) All participants who would have been eligible for the target trial were included in the study;<br>and<br>(ii) For each participant, start of follow up and start of intervention coincided.	Intervention status is well defined;<br>and<br>Intervention definition is based solely on information collected at the time of intervention.	Any deviations from intended intervention reflected usual practice	Data were reasonably complete	(i) The methods of outcome assessment were comparable across intervention groups; and<br>(ii) The outcome measure was unlikely to be influenced by knowledge of the intervention received by study participants (i.e. is objective) or the outcome assessors were unaware of the intervention received by study participants;and<br>(iii) Any error in measuring the outcome is unrelated to intervention status.	There is clear evidence that all reported results correspond to all intended outcomes, analyses and sub-cohorts.
65	(i) Confounding expected, all known important confounding domains appropriately measured and controlled for; and<br>(ii) Reliability and validity of measurement of important domains were sufficient, such that we do not expect serious residual confounding.	(i) All participants who would have been eligible for the target trial were included in the study;<br>and<br>(ii) For each participant, start of follow up and start of intervention coincided.	Intervention status is well defined;<br>and<br>Intervention definition is based solely on information collected at the time of intervention.	Any deviations from intended intervention reflected usual practice	The analysis is unlikely to have removed the risk of bias arising from the missing data.	(i) The methods of outcome assessment were comparable across intervention groups; and<br>(ii) The outcome measure was unlikely to be influenced by knowledge of the intervention received by study participants (i.e. is objective) or the outcome assessors were unaware of the intervention received by study participants;and	(i) The outcome measurements and analyses are consistent with an a priori plan; or are clearly defined and both internally and externally consistent; and<br>(ii) There is no indication of selection of the reported analysis from among multiple analyses; and<br>(iii) There is no indication of selection of the cohort or subgroups for analysis and reporting on the basis of the

						(iii) Any error in measuring the outcome is unrelated to intervention status.	results.
66	At least one important domain was not appropriately measured (e.g. detailed clinical information such as laboratory values or comprehensive medical histories)	(i) All participants who would have been eligible for the target trial were included in the study;<br>and<br>(ii) For each participant, start of follow up and start of intervention coincided.	Intervention status is well defined;<br>and<br>Intervention definition is based solely on information collected at the time of intervention.	Any deviations from intended intervention reflected usual practice	Data were reasonably complete	(i) The methods of outcome assessment were comparable across intervention groups; and<br>(ii) The outcome measure was unlikely to be influenced by knowledge of the intervention received by study participants (i.e. is objective) or the outcome assessors were unaware of the intervention received by study participants;and<br>(iii) Any error in measuring the outcome is unrelated to intervention status.	(i) The outcome measurements and analyses are consistent with an a priori plan; or are clearly defined and both internally and externally consistent; and<br>(ii) There is no indication of selection of the reported analysis from among multiple analyses; and<br>(iii) There is no indication of selection of the cohort or subgroups for analysis and reporting on the basis of the results.
67	(i) Confounding expected, all known important confounding domains appropriately measured and controlled for;<br>and<br>(ii) Reliability and validity of measurement of important domains were sufficient, such that we do not expect serious residual confounding.	(i) All participants who would have been eligible for the target trial were included in the study;<br>and<br>(ii) For each participant, start of follow up and start of intervention coincided.	Intervention status is well defined;<br>and<br>Intervention definition is based solely on information collected at the time of intervention.	Any deviations from intended intervention reflected usual practice	Data were reasonably complete	(i) The methods of outcome assessment were comparable across intervention groups; and<br>(ii) The outcome measure was unlikely to be influenced by knowledge of the intervention received by study participants (i.e. is objective) or the outcome assessors were unaware of the intervention received by study participants;and<br>(iii) Any error in measuring the outcome is unrelated to intervention status.	(i) The outcome measurements and analyses are consistent with an a priori plan; or are clearly defined and both internally and externally consistent; and<br>(ii) There is no indication of selection of the reported analysis from among multiple analyses; and<br>(iii) There is no indication of selection of the cohort or subgroups for analysis and reporting on the basis of the results.
68	(i) Confounding expected, all known important confounding	(i) Selection into the study may have been related to intervention	Intervention status is well defined;<br>and<br>Intervention	Any deviations from intended intervention reflected usual practice	Data were reasonably complete	(i) The methods of outcome assessment were comparable	(i) The outcome measurements and analyses are consistent with an a priori

	domains appropriately measured and controlled for; and<br>(ii) Reliability and validity of measurement of important domains were sufficient, such that we do not expect serious residual confounding.	and outcome; and The authors used appropriate methods to adjust for the selection bias;	definition is based solely on information collected at the time of intervention.			across intervention groups; and<br>(ii) The outcome measure was unlikely to be influenced by knowledge of the intervention received by study participants (i.e. is objective) or the outcome assessors were unaware of the intervention received by study participants;and<br>(iii) Any error in measuring the outcome is unrelated to intervention status.	plan; or are clearly defined and both internally and externally consistent; and<br>(ii) There is no indication of selection of the reported analysis from among multiple analyses; and<br>(iii) There is no indication of selection of the cohort or subgroups for analysis and reporting on the basis of the results.
69	(i) Confounding expected, all known important confounding domains appropriately measured and controlled for; and<br>(ii) Reliability and validity of measurement of important domains were sufficient, such that we do not expect serious residual confounding.	(i) All participants who would have been eligible for the target trial were included in the study;<br>and<br>(ii) For each participant, start of follow up and start of intervention coincided.	Intervention status is well defined;<br>and<br>Intervention definition is based solely on information collected at the time of intervention.	Any deviations from intended intervention reflected usual practice	Data were reasonably complete<br>and<br>missForest multiple imputation method was used to impute variables	(i) The methods of outcome assessment were comparable across intervention groups; and<br>(ii) The outcome measure was unlikely to be influenced by knowledge of the intervention received by study participants (i.e. is objective) or the outcome assessors were unaware of the intervention received by study participants;and<br>(iii) Any error in measuring the outcome is unrelated to intervention status.	(i) The outcome measurements and analyses are consistent with an a priori plan; or are clearly defined and both internally and externally consistent; and<br>(ii) There is no indication of selection of the reported analysis from among multiple analyses; and<br>(iii) There is no indication of selection of the cohort or subgroups for analysis and reporting on the basis of the results.
70	(i) Confounding expected, all known important confounding domains appropriately measured and controlled for; and<br>(ii) Reliability and validity of measurement of important domains were sufficient, such	(i) All participants who would have been eligible for the target trial were included in the study;<br>and<br>(ii) For each participant, start of follow up and start of intervention coincided.	Intervention status is well defined;<br>and<br>Intervention definition is based solely on information collected at the time of intervention.	Any deviations from intended intervention reflected usual practice	Data were reasonably complete	(i) The methods of outcome assessment were comparable across intervention groups; and<br>(ii) The outcome measure was unlikely to be influenced by knowledge of the intervention received by	(i) The outcome measurements and analyses are consistent with an a priori plan; or are clearly defined and both internally and externally consistent; and<br>(ii) There is no indication of selection of the reported analysis from

	that we do not expect serious residual confounding.					study participants (i.e. is objective) or the outcome assessors were unaware of the intervention received by study participants;and (iii) Any error in measuring the outcome is unrelated to intervention status.	among multiple analyses; and (iii) There is no indication of selection of the cohort or subgroups for analysis and reporting on the basis of the results.
71	(i) Confounding expected, all known important confounding domains appropriately measured and controlled for; and (ii) Reliability and validity of measurement of important domains were sufficient, such that we do not expect serious residual confounding.	(i) All participants who would have been eligible for the target trial were included in the study; and (ii) For each participant, start of follow up and start of intervention coincided.	Intervention status is well defined; and Intervention definition is based solely on information collected at the time of intervention.	Any deviations from intended intervention reflected usual practice	Data were reasonably complete	(i) The methods of outcome assessment were comparable across intervention groups; and (ii) The outcome measure was unlikely to be influenced by knowledge of the intervention received by study participants (i.e. is objective) or the outcome assessors were unaware of the intervention received by study participants;and (iii) Any error in measuring the outcome is unrelated to intervention status.	(i) The outcome measurements and analyses are consistent with an a priori plan; or are clearly defined and both internally and externally consistent; and (ii) There is no indication of selection of the reported analysis from among multiple analyses; and (iii) There is no indication of selection of the cohort or subgroups for analysis and reporting on the basis of the results.
72	(i) Confounding expected, all known important confounding domains appropriately measured and controlled for; and (ii) Reliability and validity of measurement of important domains were sufficient, such that we do not expect serious residual confounding.	(i) All participants who would have been eligible for the target trial were included in the study; and (ii) For each participant, start of follow up and start of intervention coincided.	Intervention status is well defined; and Intervention definition is based solely on information collected at the time of intervention.	Any deviations from intended intervention reflected usual practice	Data were reasonably complete and Missing data were multiply imputed using multivariate imputation by chained equations, and the final results were pooled using the results obtained from the 20 imputed data sets	(ii) The outcome measure was subjective (i.e. vulnerable to influence by knowledge of the intervention received by study participants); and The outcome was assessed by assessors aware of the intervention received by study	(i) The outcome measurements and analyses are consistent with an a priori plan; or are clearly defined and both internally and externally consistent; and (ii) There is no indication of selection of the reported analysis from among multiple analyses; and (iii) There is no indication of selection of the cohort or subgroups for analysis and reporting on the basis of the results.

73	(i) Confounding expected, all known important confounding domains appropriately measured and controlled for; and (ii) Reliability and validity of measurement of important domains were sufficient, such that we do not expect serious residual confounding.	(i) All participants who would have been eligible for the target trial were included in the study; and (ii) For each participant, start of follow up and start of intervention coincided.	Intervention status is well defined; and Intervention definition is based solely on information collected at the time of intervention.	Any deviations from intended intervention reflected usual practice	Data were reasonably complete	(i) The methods of outcome assessment were comparable across intervention groups; and (ii) The outcome measure was unlikely to be influenced by knowledge of the intervention received by study participants (i.e. is objective) or the outcome assessors were unaware of the intervention received by study participants;and (iii) Any error in measuring the outcome is unrelated to intervention status. (some of the outcomes were self reported)	(i) The outcome measurements and analyses are consistent with an a priori plan; or are clearly defined and both internally and externally consistent; and (ii) There is no indication of selection of the reported analysis from among multiple analyses; and (iii) There is no indication of selection of the cohort or subgroups for analysis and reporting on the basis of the results.
74	(i) Confounding expected, all known important confounding domains appropriately measured and controlled for; and (ii) Reliability and validity of measurement of important domains were sufficient, such that we do not expect serious residual confounding.	(i) All participants who would have been eligible for the target trial were included in the study; and (ii) For each participant, start of follow up and start of intervention coincided.	Intervention status is well defined; and Intervention definition is based solely on information collected at the time of intervention.	Any deviations from intended intervention reflected usual practice	Data were reasonably complete and Rosenbaum approach was used to address missing covariate values	(i) The methods of outcome assessment were comparable across intervention groups; and (ii) The outcome measure was unlikely to be influenced by knowledge of the intervention received by study participants (i.e. is objective) or the outcome assessors were unaware of the intervention received by study participants;and (iii) Any error in measuring the outcome is unrelated to intervention status.	(i) The outcome measurements and analyses are consistent with an a priori plan; or are clearly defined and both internally and externally consistent; and (ii) There is no indication of selection of the reported analysis from among multiple analyses; and (iii) There is no indication of selection of the cohort or subgroups for analysis and reporting on the basis of the results.
75	(i) Confounding expected, all known important confounding domains appropriately	(i) All participants who would have been eligible for the target trial were included in the	Intervention status is well defined; and Intervention definition is based solely on	Any deviations from intended intervention reflected usual practice	Data were reasonably complete	(i) The methods of outcome assessment were comparable across intervention	Defined in a previous study

	measured and controlled for; and (ii) Reliability and validity of measurement of important domains were sufficient, such that we do not expect serious residual confounding.	study; and (ii) For each participant, start of follow up and start of intervention coincided.	information collected at the time of intervention.			groups; and (ii) The outcome measure was unlikely to be influenced by knowledge of the intervention received by study participants (i.e. is objective) or the outcome assessors were unaware of the intervention received by study participants;and (iii) Any error in measuring the outcome is unrelated to intervention status.	
76	(i) Confounding expected, all known important confounding domains appropriately measured and controlled for; and (ii) Reliability and validity of measurement of important domains were sufficient, such that we do not expect serious residual confounding.	(i) All participants who would have been eligible for the target trial were included in the study; and (ii) For each participant, start of follow up and start of intervention coincided.	Intervention status is well defined; and Intervention definition is based solely on information collected at the time of intervention.	Any deviations from intended intervention reflected usual practice	Data were reasonably complete and Missing data were addressed using the Multiple Imputation by Chained Equations (MICE) method	(ii) The outcome measure was subjective (i.e. vulnerable to influence by knowledge of the intervention received by study participants); and The outcome was assessed by assessors aware of the intervention received by study	Defined a priori in study protocol
77	(i) Confounding expected, all known important confounding domains appropriately measured and controlled for; and (ii) Reliability and validity of measurement of important domains were sufficient, such that we do not expect serious residual confounding.	(i) All participants who would have been eligible for the target trial were included in the study; and (ii) For each participant, start of follow up and start of intervention coincided.	Intervention status is well defined; and Intervention definition is based solely on information collected at the time of intervention.	Any deviations from intended intervention reflected usual practice	(i) Proportions of and reasons for missing participants differ slightly across intervention groups; and (ii) The analysis is unlikely to have removed the risk of bias arising from the missing data.	(i) The methods of outcome assessment were not comparable across intervention groups; different follow-up periods	(i) The outcome measurements and analyses are consistent with an a priori plan; or are clearly defined and both internally and externally consistent; and (ii) There is no indication of selection of the reported analysis from among multiple analyses; and (iii) There is no indication of selection of the cohort or subgroups for analysis and reporting on the basis of the results.
78	(i) Confounding expected, all known	(i) All participants who would	Intervention status is well defined;	Any deviations from intended intervention	Data were reasonably complete	(i) The methods of outcome assessment	(i) The outcome measurements and analyses

	important confounding domains appropriately measured and controlled for; and (ii) Reliability and validity of measurement of important domains were sufficient, such that we do not expect serious residual confounding.	have been eligible for the target trial were included in the study; and (ii) For each participant, start of follow up and start of intervention coincided.	and Intervention definition is based solely on information collected at the time of intervention.	reflected usual practice	and The Rosenbaum approach was used to address missing covariate values	were comparable across intervention groups; and (ii) The outcome measure was unlikely to be influenced by knowledge of the intervention received by study participants (i.e. is objective) or the outcome assessors were unaware of the intervention received by study participants;and (iii) Any error in measuring the outcome is unrelated to intervention status.	are consistent with an a priori plan; or are clearly defined and both internally and externally consistent; and (ii) There is no indication of selection of the reported analysis from among multiple analyses; and (iii) There is no indication of selection of the cohort or subgroups for analysis and reporting on the basis of the results.
79	(i) Confounding expected, all known important confounding domains appropriately measured and controlled for; and (ii) Reliability and validity of measurement of important domains were sufficient, such that we do not expect serious residual confounding.	(i) All participants who would have been eligible for the target trial were included in the study; and (ii) For each participant, start of follow up and start of intervention coincided.	Intervention status is well defined; and Intervention definition is based solely on information collected at the time of intervention.	Any deviations from intended intervention reflected usual practice	Data were reasonably complete and performed multiple imputation by chained equations (MICE) with predictive mean matching, generating 20 imputed datasets for the 258 patients	(ii) The outcome measure was subjective (i.e. vulnerable to influence by knowledge of the intervention received by study participants); and The outcome was assessed by assessors aware of the intervention received by study	Defined a priori in study protocol
80	(i) Confounding expected, all known important confounding domains appropriately measured and controlled for; and (ii) Reliability and validity of measurement of important domains were sufficient, such that we do not expect serious residual confounding.	(i) Selection into the study may have been related to intervention and outcome; and The authors used appropriate methods to adjust for the selection bias;	Intervention status is well defined; and Intervention definition is based solely on information collected at the time of intervention.	Any deviations from intended intervention reflected usual practice	Data were reasonably complete and multiple imputation	(ii) The outcome measure was subjective (i.e. vulnerable to influence by knowledge of the intervention received by study participants); and The outcome was assessed by assessors aware of the intervention received by study	(i) The outcome measurements and analyses are consistent with an a priori plan; or are clearly defined and both internally and externally consistent; and (ii) There is no indication of selection of the reported analysis from among multiple analyses; and (iii) There is no indication of selection of the cohort or subgroups for analysis and reporting on the basis of the

							results.
81	(i) Confounding expected, all known important confounding domains appropriately measured and controlled for; and (ii) Reliability and validity of measurement of important domains were sufficient, such that we do not expect serious residual confounding.	(i) All participants who would have been eligible for the target trial were included in the study; and (ii) For each participant, start of follow up and start of intervention coincided.	Intervention status is well defined; and Intervention definition is based solely on information collected at the time of intervention.	Any deviations from intended intervention reflected usual practice	Data were reasonably complete	(i) The methods of outcome assessment were comparable across intervention groups; and (ii) The outcome measure was unlikely to be influenced by knowledge of the intervention received by study participants (i.e. is objective) or the outcome assessors were unaware of the intervention received by study participants;and (iii) Any error in measuring the outcome is unrelated to intervention status.	(i) The outcome measurements and analyses are consistent with an a priori plan; or are clearly defined and both internally and externally consistent; and (ii) There is no indication of selection of the reported analysis from among multiple analyses; and (iii) There is no indication of selection of the cohort or subgroups for analysis and reporting on the basis of the results.
82	At least one important domain was not appropriately measured (e.g. we could not adjust for any unmeasured confounding such as fracture comminution.)	(i) All participants who would have been eligible for the target trial were included in the study; and (ii) For each participant, start of follow up and start of intervention coincided.	Intervention status is well defined; and Intervention definition is based solely on information collected at the time of intervention.	Any deviations from intended intervention reflected usual practice	The nature of the missing data means that the risk of bias cannot be removed through appropriate analysis. only 14% follow-up rate	(ii) The outcome measure was subjective (i.e. vulnerable to influence by knowledge of the intervention received by study participants); and The outcome was assessed by assessors aware of the intervention received by study	(i) The outcome measurements and analyses are consistent with an a priori plan; or are clearly defined and both internally and externally consistent; and (ii) There is no indication of selection of the reported analysis from among multiple analyses; and (iii) There is no indication of selection of the cohort or subgroups for analysis and reporting on the basis of the results.
83	(i) Confounding expected, all known important confounding domains appropriately measured and controlled for; and (ii) Reliability and validity of measurement of important domains were	(i) All participants who would have been eligible for the target trial were included in the study; and (ii) For each participant, start of follow up and start of intervention coincided.	Intervention status is well defined; and Intervention definition is based solely on information collected at the time of intervention.	Any deviations from intended intervention reflected usual practice	(i) Proportions of and reasons for missing participants differ slightly across intervention groups; and (ii) The analysis is unlikely to have removed the risk of bias arising from the missing data.	(ii) The outcome measure was subjective (i.e. vulnerable to influence by knowledge of the intervention received by study participants); and The outcome was assessed by assessors aware	(i) The outcome measurements and analyses are consistent with an a priori plan; or are clearly defined and both internally and externally consistent; and (ii) There is no indication of selection of the reported

	sufficient, such that we do not expect serious residual confounding.					of the intervention received by study	analysis from among multiple analyses; and (iii) There is no indication of selection of the cohort or subgroups for analysis and reporting on the basis of the results.
84	At least one important domain was not appropriately measured (extent of central canal stenosis)	(i) All participants who would have been eligible for the target trial were included in the study; and (ii) For each participant, start of follow up and start of intervention coincided.	Intervention status is well defined; and Intervention definition is based solely on information collected at the time of intervention.	Any deviations from intended intervention reflected usual practice	(i) Proportions of and reasons for missing participants differ slightly across intervention groups; and (ii) The analysis is unlikely to have removed the risk of bias arising from the missing data.	(ii) The outcome measure was subjective (i.e. vulnerable to influence by knowledge of the intervention received by study participants); and The outcome was assessed by assessors aware of the intervention received by study	(i) The outcome measurements and analyses are consistent with an a priori plan; or are clearly defined and both internally and externally consistent; and (ii) There is no indication of selection of the reported analysis from among multiple analyses; and (iii) There is no indication of selection of the cohort or subgroups for analysis and reporting on the basis of the results.
85	(i) Confounding expected, all known important confounding domains appropriately measured and controlled for; and (ii) Reliability and validity of measurement of important domains were sufficient, such that we do not expect serious residual confounding.	(i) All participants who would have been eligible for the target trial were included in the study; and (ii) For each participant, start of follow up and start of intervention coincided.	Intervention status is well defined; and Intervention definition is based solely on information collected at the time of intervention.	Any deviations from intended intervention reflected usual practice	Data were reasonably complete	(i) The methods of outcome assessment were comparable across intervention groups; and (ii) The outcome measure was unlikely to be influenced by knowledge of the intervention received by study participants (i.e. is objective) or the outcome assessors were unaware of the intervention received by study participants;and (iii) Any error in measuring the outcome is unrelated to intervention status.	(i) The outcome measurements and analyses are consistent with an a priori plan; or are clearly defined and both internally and externally consistent; and (ii) There is no indication of selection of the reported analysis from among multiple analyses; and (iii) There is no indication of selection of the cohort or subgroups for analysis and reporting on the basis of the results.

Table M
Assessment of Risk of Bias Scores

ID	Bias due to confounding	Bias in selection of participants into the study	Bias in classification of interventions	Bias due to deviations from intended intervention	Bias due to missing data	Bias in measurement of outcomes	Bias in selection of the reported result	Overall risk of bias
1	Moderate	Moderate	Low	Low	Low	Low	Low	Moderate
2	Moderate	Moderate	Low	Low	Low	Low	Moderate	Moderate
3	Serious	Low	Low	Low	Low	Low	Moderate	Serious
4	Serious	Low	Low	Low	Low	Low	Moderate	Serious
5	Moderate	Moderate	Serious	Low	Moderate	Low	Moderate	Serious
6	Moderate	Low	Low	Low	Low	Low	Moderate	Moderate
7	Serious	Low	Low	Low	Low	Serious	Moderate	Serious
8	Moderate	Low	Low	Low	Low	Low	Moderate	Moderate
9	Moderate	Low	Low	Low	Low	Low	Moderate	Moderate
10	Serious	Low	Low	Low	Low	Low	Moderate	Serious
11	Serious	Low	Low	Low	Low	Low	Moderate	Serious
12	Moderate	Low	Low	Low	Low	Low	Moderate	Moderate
13	Moderate	Low	Low	Low	Low	Low	Moderate	Moderate
14	Moderate	Low	Low	Serious	Low	Low	Moderate	Serious
15	Moderate	Low	Low	Low	Low	Serious	Moderate	Serious
16	Moderate	Low	Low	Low	Moderate	Low	Moderate	Moderate
17	Serious	Low	Low	Low	Low	Low	Moderate	Serious
18	Moderate	Low	Low	Low	Low	Low	Moderate	Moderate
19	Moderate	Low	Low	Low	Low	Low	Moderate	Moderate
20	Serious	Low	Low	Low	Low	Low	Moderate	Serious
21	Moderate	Low	Low	Low	Low	Low	Moderate	Moderate
22	Serious	Low	Low	Low	Low	Serious	Moderate	Serious
23	Moderate	Low	Low	Low	Low	Low	Moderate	Moderate
24	Moderate	Low	Low	Low	Low	Low	Moderate	Moderate
25	Moderate	Low	Low	Low	Low	Low	Moderate	Moderate
26	Serious	Low	Low	Low	Low	Low	Moderate	Serious
27	Moderate	Low	Low	Low	Low	Low	Moderate	Moderate
28	Moderate	Low	Low	Low	Low	Low	Moderate	Moderate
29	Serious	Low	Low	Low	Low	Low	Moderate	Serious
30	Moderate	Low	Low	Low	Moderate	Serious	Moderate	Serious
31	Moderate	Low	Low	Low	Low	Serious	Moderate	Serious
32	Moderate	Low	Low	Low	Low	Low	Moderate	Moderate
33	Moderate	Low	Low	Low	Low	Low	Moderate	Moderate
34	Serious	Low	Low	Low	Low	Serious	Moderate	Serious
35	Moderate	Low	Low	Low	Low	Low	Moderate	Moderate
36	Serious	Moderate	Low	Low	Low	Serious	Moderate	Serious
37	Moderate	Low	Low	Low	Low	Serious	Moderate	Serious
38	Moderate	Moderate	Low	Low	Low	Low	Low	Moderate
39	Serious	Low	Serious	Low	Low	Low	Moderate	Serious
40	Serious	Low	Low	Low	Low	Low	Moderate	Serious
41	Moderate	Low	Low	Low	Low	Serious	Moderate	Serious
42	Moderate	Moderate	Low	Low	Moderate	Serious	Moderate	Serious
43	Moderate	Low	Low	Low	Low	Low	Moderate	Moderate
44	Moderate	Moderate	Low	Low	Low	Low	Moderate	Moderate
45	Serious	Low	Low	Low	Low	Low	Moderate	Serious
46	Serious	Low	Low	Low	Low	Low	Moderate	Serious
47	Moderate	Low	Low	Low	Low	Low	Moderate	Moderate
48	Moderate	Low	Low	Low	Low	Low	Moderate	Moderate
49	Moderate	Low	Low	Low	Low	Low	Moderate	Moderate
50	Moderate	Serious	Low	Low	Low	Low	Moderate	Serious
51	Serious	Low	Low	Low	Low	Low	Moderate	Serious
52	Serious	Low	Low	Low	Low	Low	Moderate	Serious
53	Moderate	Low	Low	Low	Low	Low	Low	Moderate
54	Moderate	Low	Low	Low	Low	Low	Moderate	Moderate
55	Serious	Low	Low	Low	Low	Serious	Moderate	Serious
56	Serious	Low	Low	Low	Low	Serious	Moderate	Serious
57	Serious	Low	Low	Low	Moderate	Low	Moderate	Serious
58	Serious	Low	Low	Low	Low	Low	Moderate	Serious
59	Moderate	Low	Low	Low	Low	Low	Moderate	Moderate
60	Moderate	Low	Low	Serious	Moderate	Serious	Moderate	Serious
61	Serious	Low	Low	Low	Low	Low	Moderate	Serious
62	Moderate	Low	Low	Low	Low	Low	Moderate	Moderate
63	Moderate	Low	Low	Low	Low	Serious	Moderate	Serious
64	Moderate	Low	Low	Low	Low	Low	Low	Moderate
65	Moderate	Low	Low	Low	Moderate	Low	Moderate	Moderate

66	Serious	Low	Low	Low	Low	Low	Moderate	Serious
67	Moderate	Low	Low	Low	Low	Low	Moderate	Moderate
68	Moderate	Moderate	Low	Low	Low	Low	Moderate	Moderate
69	Moderate	Low	Low	Low	Low	Low	Moderate	Moderate
70	Moderate	Low	Low	Low	Low	Low	Moderate	Moderate
71	Moderate	Low	Low	Low	Low	Low	Moderate	Moderate
72	Moderate	Low	Low	Low	Low	Serious	Moderate	Serious
73	Moderate	Low	Low	Low	Low	Low	Moderate	Moderate
74	Moderate	Low	Low	Low	Low	Low	Moderate	Moderate
75	Moderate	Low	Low	Low	Low	Low	Low	Moderate
76	Moderate	Low	Low	Low	Low	Serious	Low	Serious
77	Moderate	Low	Low	Low	Moderate	Serious	Moderate	Serious
78	Moderate	Low	Low	Low	Low	Low	Moderate	Moderate
79	Moderate	Low	Low	Low	Low	Serious	Low	Serious
80	Moderate	Moderate	Low	Low	Low	Serious	Moderate	Serious
81	Moderate	Low	Low	Low	Low	Low	Moderate	Moderate
82	Serious	Low	Low	Low	Serious	Serious	Moderate	Serious
83	Moderate	Low	Low	Low	Moderate	Serious	Moderate	Serious
84	Serious	Low	Low	Low	Moderate	Serious	Moderate	Serious
85	Moderate	Low	Low	Low	Low	Low	Moderate	Moderate

Supplementary References

1. Friis Pedersen C, Eiskjær S, Østerheden Andersen M, Yacat Carreon L, Doering P. A propensity-matched study of patients with symptomatic lumbar spinal stenosis opting for surgery versus not. Brain and Spine. 2024 Jan 1;4:102802.

2. Nugent M, Campbell DG, Lewis PL, Cuthbert AR, Solomon LB. Acetabular screws do not improve early revision rates in primary total hip arthroplasty. An instrumented registry analysis. International Orthopaedics (SICOT). 2021 Mar 1;45(3):593–604.

3. LATIJNHOUWERS DAJM, LAAS N, VERDEGAAL SHM, NELISSEN RGHH, VLIET VLIELAND TPM, KAPTIJN HH, et al. Activities and participation after primary total hip arthroplasty; posterolateral versus direct anterior approach in 860 patients. Acta Orthop. 2022 July 4;93:613–22.

4. Inacio MCS, Cafri G, Paxton EW, Kurtz SM, Namba RS. Alternative bearings in total knee arthroplasty: risk of early revision compared to traditional bearings: an analysis of 62,177 primary cases. Acta Orthop. 2013 Apr;84(2):145–52.

5. Cloney MB, Hopkins B, Dhillon E, El Tecle N, Swong K, Koski TR, et al. Anterior approach lumbar fusions cause a marked increase in thromboembolic events: Causal inferences from a propensity-matched analysis of 1147 patients. Clin Neurol Neurosurg. 2022 Dec;223:107506.

6. Egbert RC, Chan PH, Chan DP, Prentice HA, McElvany MD, Yian EH. Antibiotic-loaded bone cement vs. plain cement as an infection prophylaxis in primary elective shoulder arthroplasty. Seminars in Arthroplasty: JSES. 2023 Mar 1;33(1):123–31.

7. MacDowall A, Skeppholm M, Lindhagen L, Robinson Y, Löfgren H, Michaëlsson K, et al. Artificial disc replacement versus fusion in patients with cervical degenerative disc disease with radiculopathy: 5-year outcomes from the National Swedish Spine Register. J Neurosurg Spine. 2019 Feb 1;30(2):159–67.

8. Ogawa T, Yoshii T, Moriwaki M, Morishita S, Oh Y, Miyatake K, et al. Association between Hemiarthroplasty vs. Total Hip Arthroplasty and Major Surgical Complications among Patients with Femoral Neck Fracture. Journal of Clinical Medicine. 2020 Oct;9(10):3203.

9. Masuda S, Fujibayashi S, Takemoto M, Ota M, Onishi E, Odate S, et al. Association of two-staged surgery with systemic perioperative complications in lateral lumbar interbody fusion for adult spinal deformity: a propensity score-weighted study. Eur Spine J. 2023 Mar 1;32(3):950–6.

10. Farey JE, Cuthbert AR, Adie S, Harris IA. Bipolar Hemiarthroplasty Does Not Result in a Higher Risk of Revision Compared with Total Hip Arthroplasty for Displaced Femoral Neck Fractures: An Instrumental Variable Analysis of 36,118 Procedures from the Australian Orthopaedic Association National Joint Replacement Registry. JBJS. 2022 May 18;104(10):919.

11. Steele KM, Schwartz MH. Causal Effects of Motor Control on Gait Kinematics After Orthopedic Surgery in Cerebral Palsy: A Machine-Learning Approach. Front Hum Neurosci. 2022 June 3;16:846205.

12. Rupp MC, Lindner F, Winkler PW, Muench LN, Mehl J, Imhoff AB, et al. Clinical Effect of Isolated Lateral Closing Wedge Distal Femoral Osteotomy Compared to Medial Opening Wedge High Tibial Osteotomy for the Correction of Varus Malalignment: A Propensity Score-Matched Analysis. Am J Sports Med. 2023 Feb;51(2):437–45.

13. Yang P, He R, Lei K, Liu L, Yang L, Guo L. Clinical evaluation of the first semi-active total knee arthroplasty assisting robot made in China: a retrospective propensity score-matched cohort study. Int J Surg. 2023 June 1;109(6):1552–60.

14. Takenaka S, Mukai Y, Tateishi K, Hosono N, Fuji T, Kaito T. Clinical Outcomes After Posterior Lumbar Interbody Fusion: Comparison of Cortical Bone Trajectory and Conventional Pedicle Screw Insertion. Clinical Spine Surgery. 2017 Dec;30(10):E1411.

15. Namba RS, Prentice HA, Paxton EW, Hinman AD, Kelly MP. Commercially Prepared Antibiotic-Loaded Bone Cement and Infection Risk Following Cemented Primary Total Knee Arthroplasty. J Bone Joint Surg Am. 2020 Nov 18;102(22):1930–8.

16. Biz C, Bragazzi NL, Di Rita A, Pozzuoli A, Belluzzi E, Rodà MG, et al. Comparative analysis between Reverdin-Isham Osteotomy (RIO) and minimally invasive intramedullary nail device (MIIND) in association with AKIN osteotomy for Hallux valgus correction. Journal of Orthopaedic Surgery and Research. 2025 Feb 20;20(1):185.

17. Yamato Y, Nagata K, Kawamura N, Higashikawa A, Takeshita Y, Tozawa K, et al. Comparative Analysis of Microendoscopic and Open Laminectomy for Single-Level Lumbar Spinal Stenosis at L1–L2 or L2–L3. World Neurosurgery. 2024 Mar 1;183:e408–14.

18. Jain NB, Ayers GD, Fan R, Kuhn JE, Warner JJP, Baumgarten KM, et al. Comparative Effectiveness of Operative Versus Nonoperative Treatment for Rotator Cuff Tears: A Propensity Score Analysis From the ROW Cohort. Am J Sports Med. 2019 Nov;47(13):3065–72.

19. Zhou C, Selles RW, Slijper HP, Feitz R, van Kooij Y, Moojen TM, et al. Comparative Effectiveness of Percutaneous Needle Aponeurotomy and Limited Fasciectomy for Dupuytren's Contracture: A Multicenter Observational Study. Plastic and Reconstructive Surgery. 2016 Oct;138(4):837.

20. Catalino MP, Pate V, Stürmer T, Bhowmick DA. Comparative Propensity-Weighted Mortality After Isolated Acute Traumatic Axis Fractures in Older Adults. Geriatr Orthop Surg Rehabil. 2020 Jan 1;11:2151459320911867.

21. Mahamid A, Laver L, Maman D, Abu Elhija A, Haj Yahya M, Haverkamp D, et al. Comparing Early Outcomes and Complications Between Total Ankle Arthroplasty and Ankle Arthrodesis in Patients with Ankle Osteoarthritis: Big Data Analysis. Journal of Clinical Medicine. 2025 Jan;14(9):2909.

22. Sugiura T, Okuda S, Takenaka S, Nagamoto Y, Matsumoto T, Takahashi Y, et al. Comparing Investigation Between Bilateral Partial Laminectomy and Posterior Lumbar Interbody Fusion for Mild Degenerative Spondylolisthesis. Clinical Spine Surgery. 2021 Aug;34(7):E403.

23. Trisolino G, Stilli S, Gallone G, Leite PS, Pignatti G. Comparison between modified Dunn procedure and in situ fixation for severe stable slipped capital femoral epiphysis. Acta Orthopaedica. 2018 Mar 4;211–6.

24. Jevotovsky DS, Thirukumaran CP, Rubery PT. Creating value in spine surgery: using patient reported outcomes to compare the short-term impact of different orthopedic surgical procedures. Spine J. 2019 Nov;19(11):1850–7.

25. Huang KT, Hazzard M, Thomas S, Chagoya G, Berg RWV, Adogwa O, et al. Differences in the outcomes of anterior versus posterior interbody fusion surgery of the lumbar spine: A propensity score-controlled cohort analysis of 10,941 patients. Journal of Clinical Neuroscience. 2015 May 1;22(5):848–53.

26. Takashima K, Sakai T, Amano S, Hamada H, Ando W, Takao M, et al. Does a computed tomography-based navigation system reduce the risk of dislocation after total hip arthroplasty in patients with osteonecrosis of the femoral head? A propensity score analysis. J Artif Organs. 2020 Sept 1;23(3):247–54.

27. Häberli J, Bieri KS, Aghayev E, Eggli S, Henle P. Dynamic intraligamentary stabilization of anterior cruciate ligament repair: hardware removal has no effect on knee laxity at 2-year follow-up. Arch Orthop Trauma Surg. 2019 May 1;139(5):639–44.

28. Ozdag Y, Koshinski JL, Hayes DS, Cornwell D, Garcia VC, Klena JC, et al. Early Rates of Revision Surgery in Endoscopic and Open Carpal Tunnel Release. The Journal of Hand Surgery. 2025 Jan 1;50(1):60–9.

29. Sawaguchi A, Momosaki R, Hasebe K, Chono M, Kasuga S, Abo M. Effectiveness of preoperative physical therapy for older patients with hip fracture. Geriatrics & Gerontology International. 2018;18(7):1003–8.

30. Klaassen AD, Jorritsma W, Willigenburg NW, Gerritsma CLE, Have BLEFT, Moojen DJF, et al. Effectiveness of total hip arthroplasty versus non-surgery on patient-reported hip function at 3 months: a target trial emulation study of patients with osteoarthritis. Acta Orthopaedica. 2025 Apr 14;96:310–6.

31. Unterfrauner I, Muñoz Laguna J, Serra-Burriel M, Burgstaller JM, Uçkay I, Farshad M, et al. Fusion versus decompression alone for lumbar degenerative spondylolisthesis and spinal stenosis: a target trial emulation with index trial benchmarking. Eur Spine J. 2024 Nov 1;33(11):4281–91.

32. Lecoq FA, Parienti JJ, Murison J, Ruiz N, Bouacida K, Besse J, et al. Graft Choice and the Incidence of Osteoarthritis After Anterior Cruciate Ligament Reconstruction: A Causal Analysis From a Cohort of 541 Patients. Am J Sports Med. 2018 Oct 1;46(12):2842–50.

33. Edelstein AI, Dillingham TR, McGinley EL, Pezzin LE. Hemiarthroplasty Versus Total Hip Arthroplasty for Femoral Neck Fracture in Elderly Patients: Twelve-Month Risk of Revision and Dislocation in an Instrumental Variable Analysis of Medicare Data. J Bone Joint Surg Am. 2023 Nov 1;105(21):1695–702.

34. Hinman AD, Chang RN, Kelly MP, Fasig BH, Paxton EW, Royse KE. Impact of Metaphyseal Sleeves and Porous Cones on Risk Reduction for Revision Total Knee Arthroplasty: Aseptic Re-Revision in a United States Integrated Health Care System. The Journal of Arthroplasty. 2025 Sept 1;40(9):2375-2380.e1.

35. Hatano M, Sasabuchi Y, Isogai T, Ishikura H, Tanaka T, Tanaka S, et al. Increased early complications after total hip arthroplasty compared with hemiarthroplasty in older adults with a femoral neck fracture: a nationwide retrospective cohort study. The Bone & Joint Journal. 2024 Sept 1;106-B(9):986–93.

36. Rongen JJ, Rovers MM, van Tienen TG, Buma P, Hannink G. Increased risk for knee replacement surgery after arthroscopic surgery for degenerative meniscal tears: a multi-center longitudinal observational study using data from the osteoarthritis initiative. Osteoarthritis Cartilage. 2017 Jan;25(1):23–9.

37. Paxton EW, Inacio MCS, Kurtz S, Love R, Cafri G, Namba RS. Is there a difference in total knee arthroplasty risk of revision in highly crosslinked versus conventional polyethylene? Clin Orthop Relat Res. 2015 Mar;473(3):999–1008.

38. Mirzayan R, Chang RN, Royse KE, Reyes CE, Prentice HA, Maletis GB. Is There a Hamstring Autograft Diameter Threshold for Anterior Cruciate Ligament Reconstruction? Orthopaedic Journal of Sports Medicine. 2025 Feb 1;13(2):23259671241305427.

39. Kudo Y, Okano I, Toyone T, Matsuoka A, Maruyama H, Yamamura R, et al. Lateral lumbar interbody fusion in revision surgery for restenosis after posterior decompression. Neurosurg Focus. 2020 Sept;49(3):E11.

40. Gonzalez GA, Corso K, Kothari P, Franco D, Porto G, Miao J, et al. Lumbar Synovial Cysts-Should You Fuse or Not? Neurosurgery. 2023 May 1;92(5):1013–20.

41. Mohanty S, Stephan SR, Mikhail C, Platt A, Bakhsheshian J, Hassan FM, et al. Maintaining stability at the lumbosacral-pelvic region in adult spinal deformity surgery without sacroiliac joint fusion: are 4 pelvic screws superior to 2 pelvic screws? Journal of Neurosurgery: Spine. 2024 Dec 27;42(3):320–30.

42. Paxton EW, Inacio MCS, Namba RS, Love R, Kurtz SM. Metal-on-conventional polyethylene total hip arthroplasty bearing surfaces have a higher risk of revision than metal-on-highly crosslinked polyethylene: results from a US registry. Clin Orthop Relat Res. 2015 Mar;473(3):1011–21.

43. Silva PS, Jardim A, Pereira J, Sousa R, Vaz R, Pereira P. Minimally invasive fusion surgery for patients with degenerative spondylolisthesis and severe lumbar spinal stenosis: a comparative study between MIDLIF and TLIF. Eur Spine J. 2023 Sept;32(9):3210–7.

44. Leitner L, Bratschitsch G, Kostwein A, Sadoghi P, Smolle M, Leithner A, et al. More help than harm: surgery for metastatic spinal cord compression is associated with more favorable overall survival within a propensity score analysis. Eur Spine J. 2023 July 1;32(7):2468–78.

45. Hoepelman RJ, Beeres FJP, Beks RB, Sweet AAR, Ijpma FF, Lansink KWW, et al. Non-operative vs. operative treatment for multiple rib fractures after blunt thoracic trauma: a multicenter prospective cohort study. Eur J Trauma Emerg Surg. 2023 Feb;49(1):461–71.

46. Burn E, Weaver J, Morales D, Prats-Uribe A, Delmestri A, Strauss VY, et al. Opioid use, postoperative complications, and implant survival after unicompartmental versus total knee replacement: a population-based network study. The Lancet Rheumatology. 2019 Dec 1;1(4):e229–36.

47. Major Extremity Trauma Research Consortium (METRC). Outcomes Following Severe Distal Tibial, Ankle, and/or Mid/Hindfoot Trauma: Comparison of Limb Salvage and Transtibial Amputation (OUTLET). J Bone Joint Surg Am. 2021 Sept 1;103(17):1588–97.

48. Munting E, Röder C, Sobottke R, Dietrich D, Aghayev E, on behalf of the Spine Tango Contributors. Patient outcomes after laminotomy, hemilaminectomy, laminectomy and laminectomy with instrumented fusion for spinal canal stenosis: a propensity score-based study from the Spine Tango registry. Eur Spine J. 2015 Feb 1;24(2):358–68.

49. Lin WY, Lee CC, Hsu CW, Huang KY, Lyu SR. Patients with knee osteoarthritis undergoing total knee arthroplasty have a lower risk of subsequent severe cardiovascular events: propensity score and instrumental variable analysis. PLoS One. 2015;10(5):e0127454.

50. MacDowall A, Heary RF, Holy M, Lindhagen L, Olerud C. Posterior foraminotomy versus anterior decompression and fusion in patients with cervical degenerative disc disease with radiculopathy: up to 5 years of outcome from the national Swedish Spine Register. Journal of Neurosurgery: Spine. 2019 Nov 15;32(3):344–52.

51. Chan RWY, Chiang YH, Lin HC, Chang CY, Tsou YS. Postoperative 30-Day Comparative Complications of Multilevel Anterior Cervical Discectomy and Fusion and Laminoplasty for Cervical Spondylotic Myelopathy: An Evidence in Reaching Consensus. Diagnostics. 2023 Jan;13(12):2024.

52. Lu Y, Jurgensmeier K, Lamba A, Yang L, Hevesi M, Camp CL, et al. Posttraumatic Arthritis After Anterior Cruciate Ligament Injury: Machine Learning Comparison Between Surgery and Nonoperative Management. Am J Sports Med. 2025 Apr 1;53(5):1050–60.

53. Hardwick-Morris M, Carlton S, Twiggs J, Miles B, Liu D. Pre- and postoperative physiotherapy using a digital application decreases length of stay without reducing patient outcomes following total knee arthroplasty. Arthroplasty. 2022 Aug 2;4(1):30.

54. Millett PJ, Espinoza C, Horan MP, Ho CP, Warth RJ, Dornan GJ, et al. Predictors of outcomes after arthroscopic transosseous equivalent rotator cuff repair in 155 cases: a propensity score weighted analysis of knotted and knotless self-reinforcing repair techniques at a minimum of 2 years. Arch Orthop Trauma Surg. 2017 Oct;137(10):1399–408.

55. Baumbach SF, Urresti-Gundlach M, Braunstein M, Borgmann L, Böcker W, Vosseller JT, et al. Propensity Score–Matched Analysis of Arthroscopically Assisted Ankle Facture Treatment Versus Conventional Treatment. Foot Ankle Int. 2021 Apr;42(4):400–8.

56. Katz JN, Collins JE, Brophy RH, Cole BJ, Cox CL, Guermazi A, et al. Radiographic Changes Five Years After Treatment of Meniscal Tear and Osteoarthritic Changes. Arthritis Care & Research. 2025;77(3):359–65.

57. Schwartz MH, Ries AJ. Rectus femoris transfer in children with cerebral palsy: comparing a propensity score-matched observational study to a randomized controlled trial. Developmental Medicine & Child Neurology. 2021;63(2):196–203.

58. El-Galaly A, Nielsen PT, Kappel A, Jensen SL. Reduced survival of total knee arthroplasty after previous unicompartmental knee arthroplasty compared with previous high tibial osteotomy: a propensity-score weighted mid-term cohort study based on 2,133 observations from the Danish Knee Arthroplasty Registry. Acta Orthop. 2020 Apr;91(2):177–83.

59. Masuda S, Fukasawa T, Takeuchi M, Fujibayashi S, Otsuki B, Murata K, et al. Reoperation Rates of Microendoscopic Discectomy Compared With Conventional Open Lumbar Discectomy: A Large-database Study. Clin Orthop Relat Res. 2023 Jan 1;481(1):145–54.

60. Subramanian A, Adejuyigbe B, Niknam K, Gomez-Alvarado F, Morshed S, Shearer D. Retrospective cohort study analyzing outcomes of the SIGN Fin Nail in adult femoral fractures using the retrograde approach. Journal of Orthopaedics. 2024 Aug 1;54:103–7.

61. Mori Y, Takegami Y, Tokutake K, Oka Y, Imagama S. Retrospective Comparative Study of Clinical Outcomes and Cost-Effectiveness with Bone Substitutes on Volar Locking Plate Fixation of Unstable Distal Radial Fractures in the Elderly. J Hand Surg Asian-Pac Vol. 2023 Feb;28(01):61–8.

62. Valsamis EM, Prats-Uribe A, Koblbauer I, Cole S, Sayers A, Whitehouse MR, et al. Reverse total shoulder replacement versus anatomical total shoulder replacement for osteoarthritis: population based cohort study using data from the National Joint Registry and Hospital Episode Statistics for England. BMJ. 2024 Apr 30;385:e077939.

63. Farey JE, Cuthbert AR, Adie S, Harris IA. Revision Risk After Unipolar or Bipolar Hemiarthroplasty for Femoral Neck Fractures: An Instrumental Variable Analysis of 62,875 Procedures from the Australian Orthopaedic Association National Joint Replacement Registry. J Bone Joint Surg Am. 2021 Feb 3;103(3):195–204.

64. Honda A, Iizuka Y, Michihata N, Morita K, Mieda T, Takasawa E, et al. Risk factors for early mortality in elderly patients with unstable isolated C2 odontoid fracture treated with halo-vest or surgery. Sci Rep. 2023 Oct 20;13(1):17962.

65. Han SB, Song SY, Shim JH, Shin YS. Risk of a complete exchange or failure in total knee arthroplasty and unicompartmental knee arthroplasty: a nationwide population-based cohort study from South Korea. Arch Orthop Trauma Surg. 2021 Mar;141(3):477–88.

66. Yoon JR, Ko SN, Jung KY, Lee Y, Park JO, Shin YS. Risk of Revision Following Total Knee Arthroplasty or High Tibial Osteotomy: A Nationwide Propensity-Score-Matched Study. J Bone Joint Surg Am. 2019 May 1;101(9):771–8.

67. Yoo JD, Huh MH, Shin YS. Risk of revision in UKA versus HTO: a nationwide propensity score-matched study. Arch Orthop Trauma Surg. 2023 June;143(6):3457–69.

68. Bendich I, Vigdorchik JM, Sharma AK, Mayman DJ, Sculco PK, Anderson C, et al. Robotic Assistance for Posterior Approach Total Hip Arthroplasty Is Associated With Lower Risk of Revision for Dislocation When Compared to Manual Techniques. The Journal of Arthroplasty. 2022 June 1;37(6):1124–9.

69. Bendich I, Chiu YF, Sarpong N, Gonzalez Della Valle A, Su E, McLawhorn A. Robotic-assistance and computer-navigation have similar rates of intraoperative fracture and return to the operating room within 1 year to fluoroscopy-only direct anterior total hip arthroplasty. HIP International. 2025 Mar 1;35(2):116–23.

70. Mohanty S, Barchick S, Kadiyala M, Lad M, Rouhi AD, Vadali C, et al. Should patients with lumbar stenosis and grade I spondylolisthesis be treated differently based on spinopelvic alignment? A retrospective, two-year, propensity matched, comparison of patient-reported outcome measures and clinical outcomes from multiple sites within a single health system. Spine J. 2023 Jan;23(1):92–104.

71. Sheean AJ, Jin Y, Amendola A, Huston LJ, Brophy RH, Cox CL, et al. Successful Medial Meniscal Repair Reduces Knee Pain 10 Years After Anterior Cruciate Ligament Reconstruction: Exploring the Consequences of Subsequent Surgery With Causal Mediation Analysis in the MOON Cohort. Am J Sports Med. 2025 Mar 1;53(4):769–76.

72. Canouï E, Zarrouk V, Canouï-Poitrine F, Desmoulin U, Leflon V, Allaham W, et al. Surgery is safe and effective when indicated in the acute phase of hematogenous pyogenic vertebral osteomyelitis. Infectious Diseases. 2019 Apr 3;51(4):268–76.

73. Ioannidis I, Mohammad Ismail A, Forssten MP, Ahl R, Cao Y, Borg T, et al. Surgical management of displaced femoral neck fractures in patients with dementia: a comparison in mortality between hemiarthroplasty and pins/screws. Eur J Trauma Emerg Surg. 2022 Apr;48(2):1151–8.

74. Ramme AJ, Robbins CB, Patel KA, Carpenter JE, Bedi A, Gagnier JJ, et al. Surgical Versus Nonsurgical Management of Rotator Cuff Tears: A Matched-Pair Analysis. J Bone Joint Surg Am. 2019 Oct 2;101(19):1775–82.

75. Farhan-Alanie MM, Gallacher D, Craig P, Griffin J, Kozdryk J, Mason J, et al. The Effects of Computer Navigation and Patient-Specific Instrumentation on Risk of Revision, PROMs, and Mortality Following Primary TKR: An Analysis of National Joint Registry Data. JBJS. 2025 Apr 16;107(8):829.

76. Reingrittha P, Benjawongsathien K, Visuthisakchai S. The Efficacy of Posterior Fasciotomy Versus Inserted Vacuum Drainage in Reducing Postoperative Surgical Site Infection in Open Achilles Tendon Repair: A Prospective Cohort Study With Inverse Probability Treatment Weight Propensity Score Analysis. The Journal of Foot and Ankle Surgery. 2023 Mar 1;62(2):222–7.

77. Ramsay N, Close JCT, Harris IA, Harvey LA. The impact of fixation type for intertrochanteric femoral fracture on patient survival. ANZ J Surg. 2023;93(7–8):1917–23.

78. Wernecke GC, Jin XZ, Lin JL, Harris IA. The Impact of Surgical Approach on 90-Day Prosthetic Joint Infection After Total Hip Replacement — A Population-Based, Propensity Score-Matched Cohort Study. The Journal of Arthroplasty. 2024 Jan 1;39(1):151–6.

79. Dornan GJ, Ruzbarsky JJ, Comfort SM, Ernat JJ, Martin MD, Briggs KK, et al. Two-Year Outcomes of Primary Arthroscopic Surgery in Patients with Femoroacetabular Impingement: A Comparative Study of Labral Repair and Labral Reconstruction. JBJS. 2024 Oct 2;106(19):1757.

80. Prats-Uribe A, Kolovos S, Berencsi K, Carr A, Judge A, Silman A, et al. Unicompartmental compared with total knee replacement for patients with multimorbidities: a cohort study using propensity score stratification and inverse probability weighting. Health Technol Assess. 2021 Nov;25(66):1–126.

81. Hahn HM, Cook KH, Lee IJ, Park DH, Park MC. Use of Acellular Dermal Matrix in Treatment of Congenital Muscular Torticollis in Patients Over Eight Years of Age. J Craniofac Surg. 2017 May;28(3):610–5.

82. Hernandez NM, Hart A, Taunton MJ, Osmon DR, Mabry TM, Abdel MP, et al. Use of Povidone-Iodine Irrigation Prior to Wound Closure in Primary Total Hip and Knee Arthroplasty: An Analysis of 11,738 Cases. J Bone Joint Surg Am. 2019 July 3;101(13):1144–50.

83. Chundi G, Dawar A, Ahn DB, Chopra AA, Joshi T, Lin SS, et al. Use of Sustained Compression to Mitigate Nonunion in Tibiotalocalcaneal Arthrodesis: A Propensity Score–Matched Nationwide Readmissions Database Analysis. JAAOS - Journal of the American Academy of Orthopaedic Surgeons. 2025 Aug 15;33(16):e956.

84. Zelenty WD, Paek S, Dodo Y, Sarin M, Shue J, Soffin E, et al. Utilization Trends of Intraoperative Neuromonitoring for Anterior Cervical Discectomy and Fusion in New York State. Spine (Phila Pa 1976). 2023 Apr 1;48(7):492–500.

85. Chen F, Chang RN, Prentice HA, Fasig BH, Paxton EW, Hug KT, et al. What Is the Survivorship of TKA With a Twin-peg or Spikes-and-keel Cementless Implant Compared With Cemented? A Registry-based Cohort Study. Clinical Orthopaedics and Related Research®. 2025 July;483(7):1288.
